# Twine virtual patient games as an online resource for undergraduate diabetes acute care education

**DOI:** 10.1186/s12909-023-04231-2

**Published:** 2023-06-07

**Authors:** Nathaniel Patrick Andrew Quail, James Graham Boyle

**Affiliations:** 1 University of Glasgow, Glasgow, G12 8QQ UK; 2 Glasgow Royal Infirmary, Glasgow, G4 0SF UK

**Keywords:** Twine, Virtual patients, Undergraduate, Diabetes

## Abstract

**Background:**

Virtual patients provide a safe way to simulate authentic clinical practice. Twine is an open-source software that can be used to create intricate virtual patient games, including elements like non-linear free text history taking and time-related changes to the game’s narrative. We evaluated the incorporation of Twine virtual patient games into a diabetes acute care online learning package for undergraduate medical students at the University of Glassgow, Scotland.

**Methods:**

Three games were developed using Twine, Wacom Intuous Pro, Autodesk SketchBook, Camtasia Studio, and simulated patients. Online material included three VP games, eight microlectures, and a single best answer multiple choice question quiz. The games were evaluated at Kirkpatrick Level 1 with an acceptability and usability questionnaire. The entire online package was evaluated at Kirkpatrick Level 2 with pre- and post-course multiple choice and confidence questions, with statistical analysis performed using paired t-tests.

**Results:**

122 of approximately 270 eligible students provided information on resource utilisation, with 96% of these students using at least one online resource. 68% of students who returned surveys used at least one VP game. 73 students provided feedback on the VP games they had played, with the majority of median responses being “agree” on positive usability and acceptability statements. The online resources were associated with a mean multiple choice score increase from 4.37 out of 10 to 7.96 out of 10 (p < 0.0001, 95% CI + 2.99 to + 4.20, n = 52) and a mean total confidence score increase from 4.86 out of 10 to 6.70 out of 10 (p < 0.0001, 95% CI + 1.37 to + 2.30, n = 48).

**Conclusions:**

Our VP games were well-received by students and promoted engagement with online material. The package of online material led to statistically significant increases in confidence and knowledge in diabetes acute care outcomes. A blueprint with supporting instructions has now been created to facilitate rapid creation of further games using Twine software.

**Supplementary Information:**

The online version contains supplementary material available at 10.1186/s12909-023-04231-2.

## Background

The COVID-19 pandemic has enforced a paradigm shift in the delivery of medical education [1]. In-person lectures and tutorials have been rapidly replaced with asynchronously accessed online material due to physical distancing measures. Face-to-face contact with patients has also been limited, with reduced clinical exposure for medical students. This rapidly enforced shift to online content occurred at a time that many educators may had greater clinical workloads [2].

Our medical school has taught diabetes acute care to undergraduate medical students using a remote online learning approach for several years. Students are given asynchronous access to online learning material, consisting of eight microlectures and a single based answer multiple choice quiz. Although this approach was generally well-received, there were several factors that prompted an attempt to improve the material. Firstly, we know that a quarter of students did not utilise any online material and therefore aimed to seek ways of improving this [3]. Secondly, the learning material was isolated from what the students would experience in their clinical practice. Situated cognition theory proposes that how individuals think in real world situations depends not only on their acquired knowledge but also the interaction with the environment they are working within [4]. Common factors that will be of importance in a healthcare setting are time limitations and interactions with patients and other healthcare professionals and it would be prudent to immerse the students within this environment as much as possible. Finally, Hickam’s dictum states that patients can have more than one simultaneously occurring pathology. The learning material originally focussed on diabetes acute care as an isolated problem. We were keen to give our senior clinical students a more authentic experience of multi-morbidity and polypharmacy while exploring the management of diabetes emergencies.

Virtual patients (VPs) simulate aspects of clinical practice using methods that can vary from high fidelity software and manikins to simple case presentation [5]. A recent meta-analysis by Kononowicz et al. has suggested VP simulations perform at least as well in conferring knowledge to students compared to traditional teaching methods and may be superior in terms of improving clinical reasoning [6]. VP games may also offer the opportunity to engage in aspects of wider patient care such as professional collaboration and clinical prioritisation [7]. VPs can also incorporate concepts such healthcare resource utilisation and longitudinal patient care [8]. Gamification is a technique that can enhance VP scenarios by using reward theory and swift feedback to encourage user engagement [9].

**Fig. 1:**
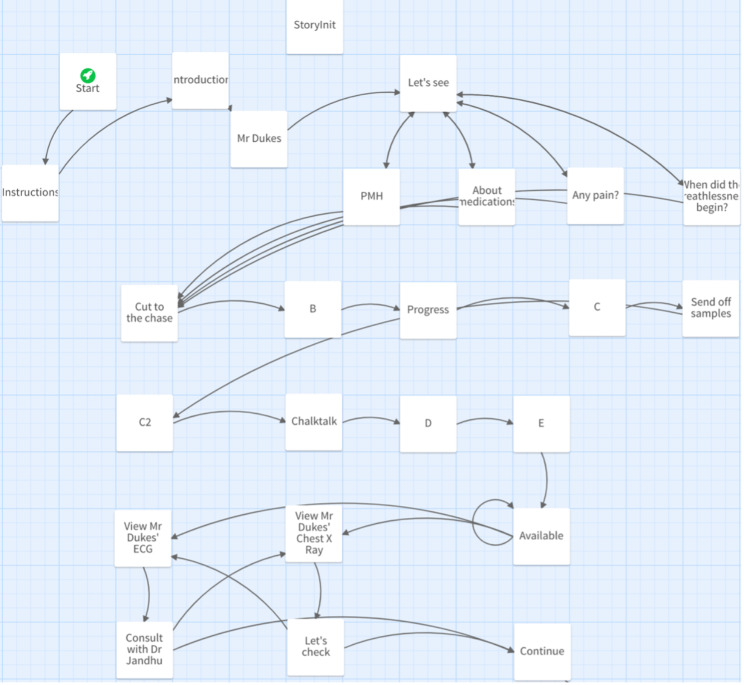
A Twine storyboard demonstrating the ability to create a complex branching network of passages, increasing the freedom of the user to navigate patient management in a pragmatic and individualised fashion.

Currently available commercial software for VP creation can be expensive and inflexible; patient journeys tend to be linear and only tutors skilled in programming can expand on core content. Twine is a free software that translates simple inputs into pre-defined code and enables the inclusion of unique features within VP games. Completed projects are published directly as Hypertext Markup Language (HTML), allowing content to be uploaded universally. Programming knowledge is not necessary to create basic scenarios. Online libraries and an active online community are available to assist in adding intricacy. Originally created as a resource for interactive storytelling, Twine boasts the capability of non-linear scenarios with extensive branching (Fig. 1).

One advantage of Twine over commercially available VP is that random number generation (RNG) can be used to influence scenario branching by, for example, randomising laboratory results or a patient’s prescribed medication. This allows multiple unique playing experiences and practice opportunities. Twine also allows the option to accurately evaluate basic free text responses and branch to a variety of content depending on input, allowing students to take unprompted histories from VPs. Twine scenarios can also utilise in-game timing, allowing students to manage deteriorating patients in a safe environment. Finally, Twine scenarios can support images, audio, and videos. This enables the addition of simulated patient interactions and the inclusion of chalk-talk videos to unpack important topics, repeat important concepts, and use multiple sensory pathways to convey information. These steps help optimise cognitive load; the burden on the working memory when it is overloaded during the process of learning [10].

Published literature on the use of Twine VP games is limited to one recent paper that describes the use of Twine to create VP scenarios for pharmacy students [11]. These scenarios were clearly intricate and well-received by both students and faculty. The authors describe that the outcome of the patient interaction depended on student choice and one of the scenarios touched on the diabetes emergencies of diabetic ketoacidosis (DKA) and hyperosmolar hyperglycaemic state (HHS). The VP games included resource utilisation, only allowing students to request a limited number of investigations, and introduced elements of multi-morbidity and polypharmacy, focusing closely on the latter. Although there were components of non-linearity within these games, including RNG, the information gathering section relied on simply clicking on pre-defined questions and there was no use of multimedia except for cartoon patient images. While timestamps were used to create a log of user choices, it does not appear that these were used to change conditions within the game. Finally, although the outcome of the scenario was given at the end of the game with some text feedback, the VP games did not delve deeper into reward theory with inclusion of unlockable achievements.

We created three VP games using Twine software to enhance the diabetes acute care online learning material for year 4 medical students at the University of Glasgow. Our three VP games cover the patient journey from admission to discharge. Each game focuses on a diabetes emergency – DKA, HHS, and hypoglycaemia – and links to specific intended learning outcomes (ILOs). The scenarios include overarching undergraduate medical themes including multi-morbidity, polypharmacy, medicines reconciliation, and healthcare resource utilisation. Free text history taking facilitates non-linearity, producing an authentic serial cue encounter. Timestamps are used to trigger in-game events, such as patient deterioration. A final score is awarded based on choices made throughout the scenarios. Awards are unlocked for navigating specific sections of the game correctly, mimicking the unlockable rewards on commercial games consoles that encourage repeated gameplay [12]. Chalk-talk videos are integrated to help students grasp difficult concepts and optimise cognitive load [10]. These three VP games were evaluated as part of a diabetes acute care online learning package using Kirkpatrick Methodology [13].

## Methods

Three serious digital games with virtual patients and chalk-talk videos were developed using Twine open-source software, Wacom Intuous Pro, Autodesk SketchBook, Camtasia Studio, and simulated videos using standardised patients [see Supplementary Material 1, 2, and 3 – please note that no multimedia is included here due to lack of consent for publication]. The three games were incorporated into an online learning package and delivered to year four medical students at the University of Glasgow. This package also included eight micro-lectures and a quiz comprised of twenty multiple-choice questions (MCQs). All resources pertained to various aspects of diabetes management and were mapped to ILOs.

Students were initially recruited at an in-person academic day, where they were provided with participant information sheets and a brief verbal description of the study. Baseline knowledge and confidence was evaluated using ten single best answer MCQs and ten confidence questions, with a range of zero (not confident) to one hundred (fully confident) [see Supplementary Material 4]. The confidence ratings were printed in increments of ten. It was decided during analysis to divide each score by ten to give a rating on the scale of zero to ten for the sake of simplicity and presentation alongside MCQ scores. The MCQs and confidence questions were each linked to the ILOs. MCQ and confidence score results were not shared with the students. Answers to the MCQs were not given to students and these questions significantly differed from those available as part of the online package. The online material was released to students directly after baseline data was obtained, via the online learning platform Moodle. Subsequent reminder emails to encourage student engagement were sent at regular intervals.

All students in attendance at the beginning of the next academic day five weeks later were asked to complete a questionnaire regarding their use of the online package [see Supplementary Material 5]. Students who utilised at least one VP game were asked to evaluate the games and their place in the undergraduate curriculum [see Supplementary Material 5]. The same ten MCQs and confidence questionnaires were completed again [see Supplementary Material 4]. Student numbers were used to pair these to baseline scores, if available, and the difference was analysed using a paired t-test. A p value of less than 0.05 was taken as statistically significant. University ethical approval for the study was obtained.

## Results

A total of 122 students provided information on utilisation of online learning resources. 83 (68%) students played a VP game at least once. 112 (92%) students accessed the microlectures, with the median viewing 8 out of 8 of these. 81 (66%) students accessed the quiz at least once. 5 (4%) students did not utilise any resource. Of the students who accessed at least one VP game, 53 (64%) accessed all three individual games, 17 (20%) accessing two individual games, and 13 (16%) accessing one game only. The median time spent using the online resources was over 1 h (IQR 30–60 min to over 1 h).

## Kirkpatrick level 1

**Fig. 2:**
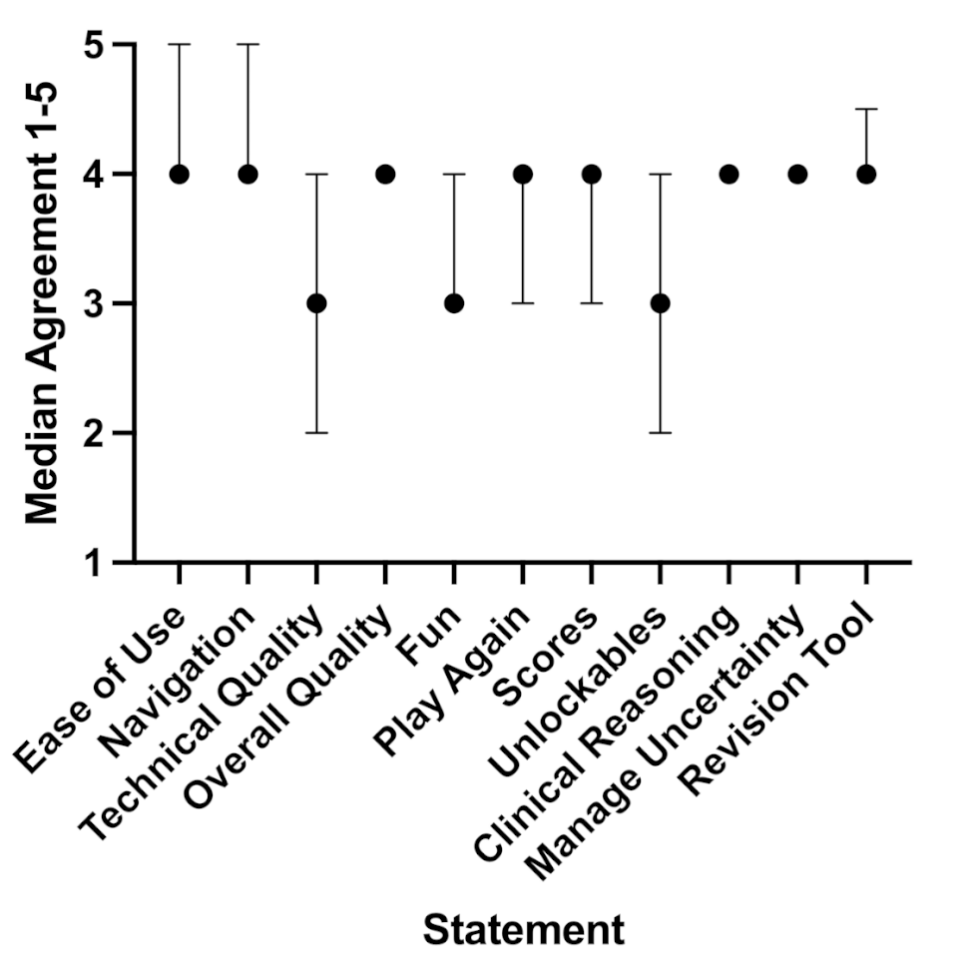
Students who attempted at least one virtual patient game were asked to what extent they agreed with positive evaluation statements on different aspects of the game: ease of use, navigation, lack of technical problems, overall quality, whether the games were fun, whether they would play the games again, whether the use of scores encouraged them to play again, whether the use of unlockables encouraged them to play the games again, whether the games were useful in developing their clinical reasoning, whether the games were useful in helping them manage uncertainty, and whether the games were useful as a revision tool. Responses were converted into a numerical rating: strongly disagree (1), disagree (2), neutral (3), agree (4), strongly agree (5). 77 eligible students completed this evaluation and their median score for each category is shown (error bars indicate IQR)

77 of 83 eligible students provided feedback on various aspects of the virtual patient games. Median ratings are shown in Fig. 2. Median ratings were 4 (agree) for statements regarding ease of use (IQR 4–5), ease of navigation (IQR 4–5), overall quality (IQR 4–4), whether students would play the games again (IQR 3–4), whether the use of scores encouraged them to play again (IQR 3–4), whether the games were useful in developing their clinical reasoning (IQR 4–4), whether the games were useful in helping them manage uncertainty (IQR 4–4), and whether the games were useful as a revision tool (IQR 4–5). The remaining statements had a median rating of 3 (neither agree nor disagree). These included there being a lack of technical problems (IQR 2–4), whether games were fun (IQR 3–4), and whether the use of unlockables encouraged repeated play-throughs (IQR 2–4).

**Fig. 3:**
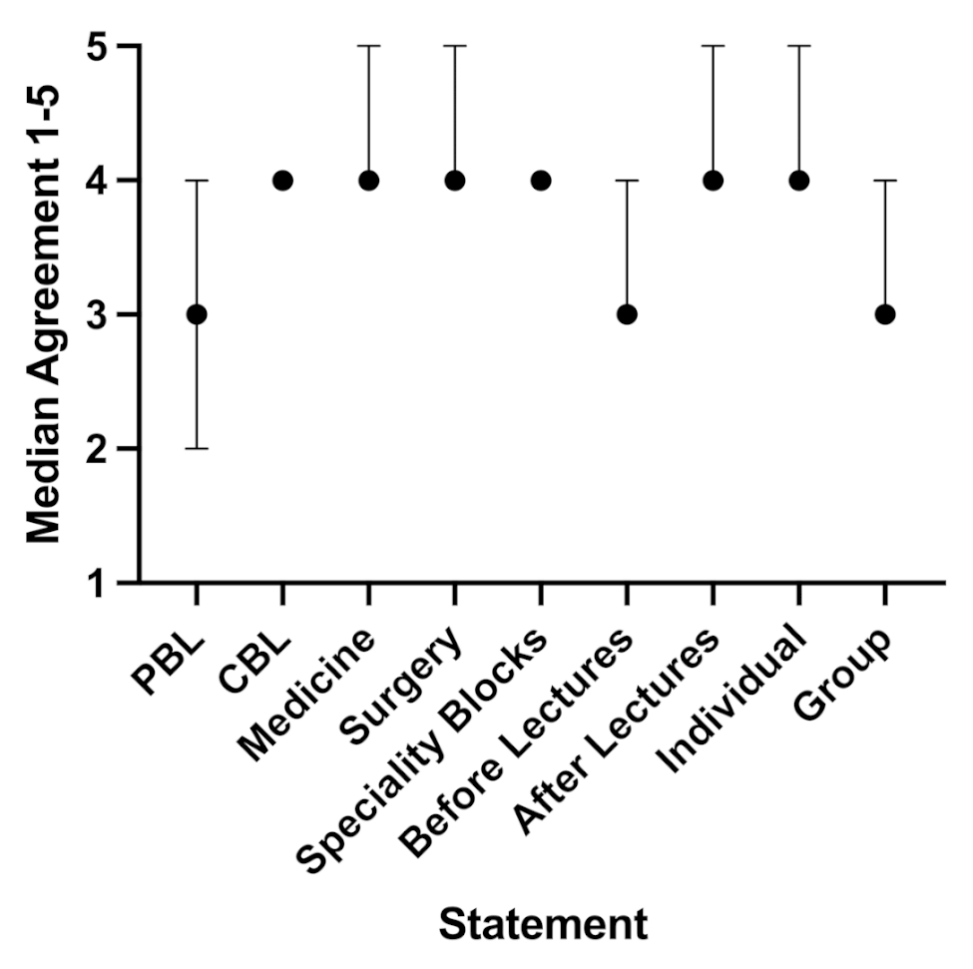
Students who attempted at least one virtual patient game were asked whether they thought virtual patient games would be useful: as part of PBL, as part of CBL, integrated into a general medicine block, integrated into a general surgery block, integrated into speciality blocks, before lectures, and after lectures. Responses were converted into a numerical rating: strongly disagree (1), disagree (2), neutral (3), agree (4), strongly agree (5). 80 eligible students completed this evaluation and their median score for each category is shown (error bars indicate IQR).

80 eligible students provided feedback on where they would prefer to see virtual patients introduced within the curriculum, and how they would like to play through the games. Median ratings are shown in Fig. 3. Median ratings were 4 (agree) for VP games being used as part of case-based learning (CBL) (IQR 4–4), integrated into a general medicine block (IQR 4–5), integrated into a general surgery block (IQR 4–5), integrated into speciality blocks (IQR 4–4), used after lectures (IQR 4–5), and worked through as an individual (IQR 4–5). A median rating of 3 was given to using VP games as part of problem-based learning (PBL) (IQR 2–4), before lectures (IQR 3–4), and working through them as a group (IQR 3–4). Finally, assuming small groups would be used, students were asked whether they would prefer to work through virtual patient games in groups of 2, in groups of 3–5, or in groups of 6–10. The median and mode was groups of 3–5 (IQR groups of 2 to groups of 3–5).

Qualitative feedback was obtained from the free text comments on the evaluation sheets provided. Students appreciated that games felt relevant to Glasgow-based encounters, enjoyed their interactive nature, and appreciated free text boxes when taking a history. The only constructive comments highlighted videos within the games not loading when accessed.

## Kirkpatrick level 2

52 students were eligible for paired analysis, with 47 students providing information on course material usage. All 47 students reported accessing at least one aspect of the online material. 38 (81%) students utilised a VP game at least once. 45 (96%) students accessed the microlectures, with the median viewing 8 out of 8 of these. 36 (77%) students accessed the quiz at least once. Of the students who accessed at least one VP game, 22 (58%) accessed all three individual games, 9 (24%) accessing two individual games, and 7 (18%) accessing one game only. The overall median time using the online resources was over 1 h (IQR over 1 h).

**Fig. 4:**
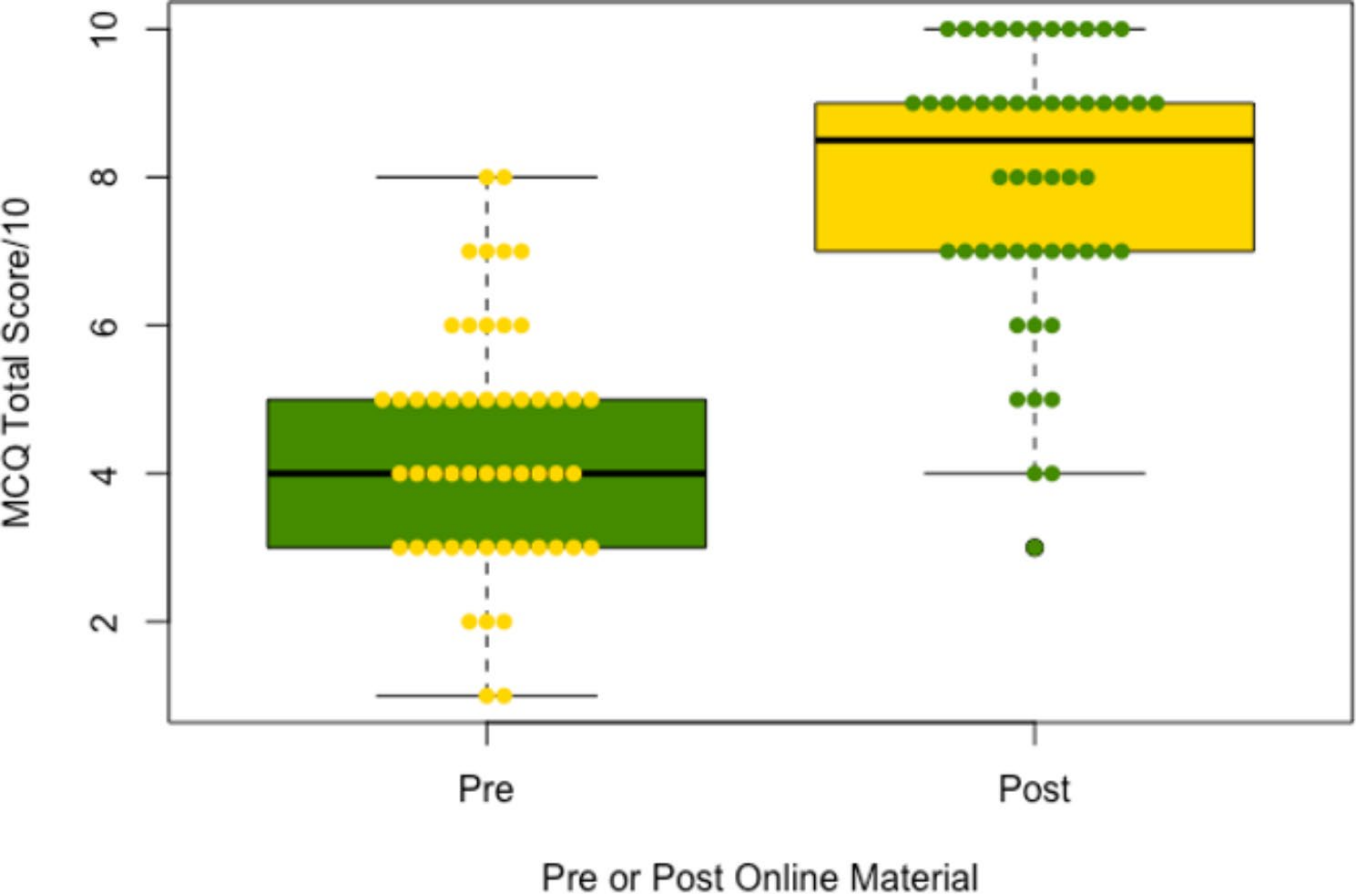
A boxplot diagram of mean total MCQ score pre and post online material. Individual student mean scores (n = 52) are shown as circles. A mean score increase from 4.37 out of 10 to 7.96 out of 10 was observed (p < 0.0001, 95% CI + 2.99 to + 4.20).

**Fig. 5:**
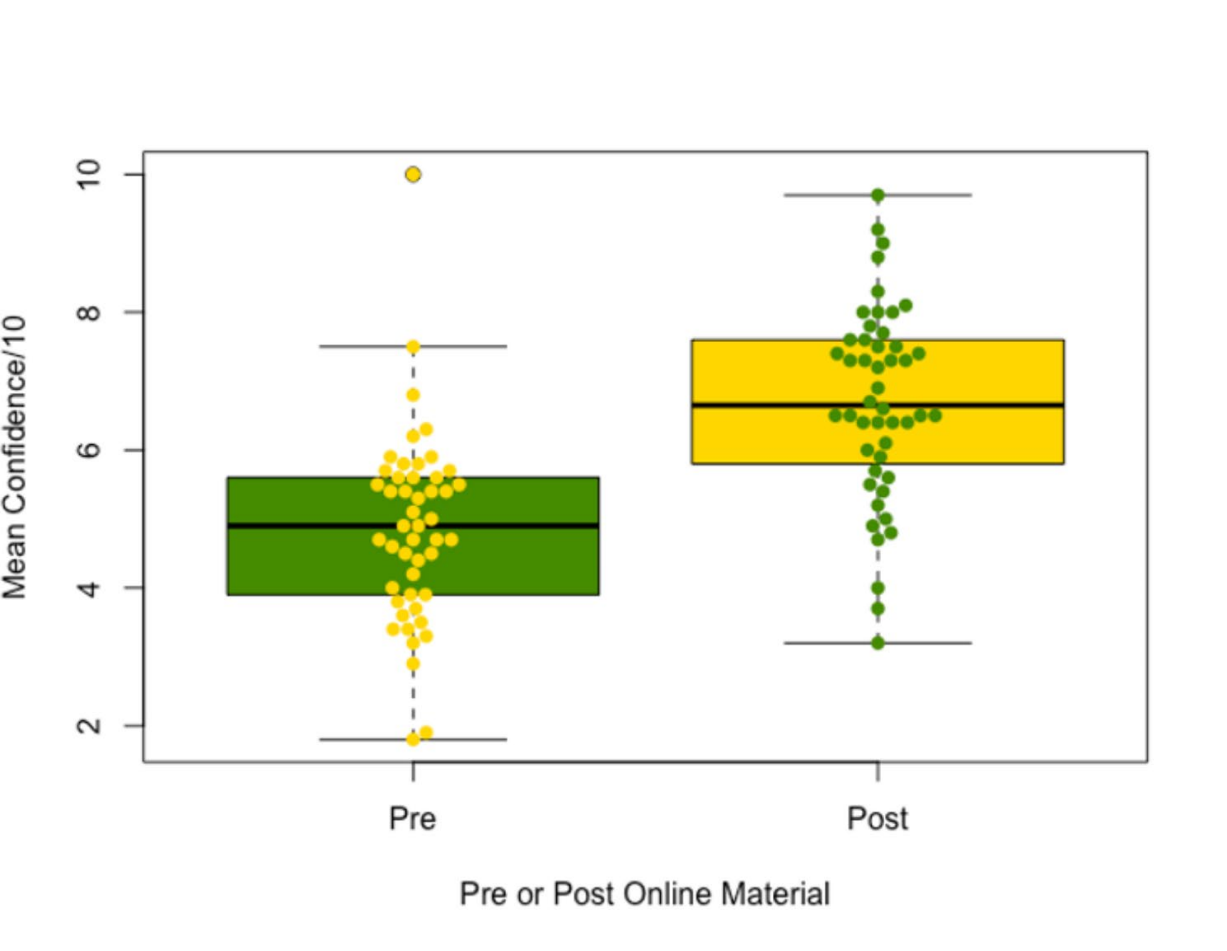
A boxplot diagram of mean total confidence scores pre and post online material. Individual student mean scores (n = 48) are shown as circles. Confidence ranges were from zero (no confidence) to ten (very confident). A mean total confidence score increase from 4.86 out of 10 to 6.70 out of 10 was observed (p < 0.0001, 95% CI + 1.37 to + 2.30, n = 48).

52 students completed both MCQs for paired analysis. The mean total MCQ score increased by 3.59 post-access, from 4.37 to 7.96 (95% confidence interval + 2.99 to + 4.20, p < 0.0001) (Fig. 4). 48 students fully completed both pre and post access confidence questionnaires. The mean total confidence score increased by 1.84 post-access, from 4.86 to 6.70 (95% confidence interval + 1.37 to + 2.30, p < 0.0001) (Fig. 5).

## Discussion

Baseline mean confidence across all ILOs was low at 4.86 out of 10. This is similar to a score of 4.69 from a previous cohort [14]. A statistically significant increase in mean confidence and knowledge was observed post-intervention. This data suggests that our online learning package of microlectures, a quiz, and VP games is effective in improving student knowledge and confidence in diabetes management. The number of students who submitted paired data was relatively low in comparison to the year total (approximately 270). This was largely due to the first academic day being poorly attended, stemming from a combination of poor weather and impending examinations.

Only 4% of responding students did not access any online resource. Although subject to self-selection bias, this suggests improved engagement compared to 25% not accessing any resource in previous years [3]. More students utilised microlectures (92%) than VP games (68%) or the quiz (66%). This may indicate that didactic teaching is perceived as being more useful for initial learning than VP games or quizzes, with our students agreeing that they would prefer to have VP games after lectures rather than before. This is in keeping with the findings of Marei et al. that VP use before formal lectures increases cognitive load [15]. Furthermore, our student feedback on the placement of VP games within the curriculum suggests they would like it in later years, after baseline clinical knowledge has been gained.

Interestingly, students indicated they would prefer to use VP games as an individual rather than as a group, at odds with findings by Marei et al. [15]. Due to feedback fatigue, we opted not to ask students to expand on their reasons behind their responses. It may depend on the design of the VP game itself as to whether students would prefer to play through as individuals or as part of a group.

A median score of 4 (agree) was achieved for every response to the gameplay except for whether it lacked technical difficulties, was fun, and whether unlockables encouraged repeated play-throughs. These all achieved a median score of 3 (neither agree nor disagree). We therefore conclude that our students found our VP games easy to use, useful for developing clinical reasoning, useful as a revision tool, and useful for managing uncertainty. Positive qualitative feedback demonstrated an appreciation of free text boxes in history gathering.

Qualitative feedback stated that videos were slow to load, potentially leading to a score of 3 for technical issues. This may have been due to mass access of the content the night before time-limited access ended, as has been observed previously [3]. The games have subsequently been deployed as non-time-limited resources, with no issues reported. As a result of this feedback, we will either look to host the content in a different manner in future or promote a more staggered resource use where possible.

Although the median score for fun was 3, the range of response from 1 (strongly disagree) to 5 (strongly agree) may represent the fact that VP games simply appeal more to certain individuals. Future research might ascertain how often students play video games and link this to their feedback.

Curiously, there was a notable difference in opinion between the utility of unlockables and a final score in encouraging repeated play-throughs, with median scores of 3 and 4 respectively. Though unlockables are given for specific choices, a score is a global measure against which students can compare their subsequent attempts, which may explain some discrepancy between ratings. Furthermore, unlockables in our VP games were contained within the game, in contrast to the trophies or achievements associated with PlayStation or XBOX respectively, which exist as permanent attachments to the gamer profile [12]. Future studies could examine the effect of permanently linking these achievements to the student’s online learning profile.

Free Twine software can utilise RNG, in-game timing, and keyword recognition in the context of multiple potential branching points to enhance the VP quality and reflect genuine clinical encounters. Overarching medical themes such as polypharmacy, multi-morbidity, and healthcare resource utilisation can be covered over and above the core curriculum. Our VP games were deemed by students to be easy to use, useful for developing clinical reasoning, and useful as a revision tool. Addressing hosting issues may improve the technical experience and utilisation of reward systems more effectively may improve engagement and repeated play-throughs.

In terms of legacy, our serious games have recently been incorporated into a Moodle virtual ward in response to the COVID-19 pandemic. We have subsequently created a simple blueprint game [see Supplementary Material 6] with associated documentation [see Supplementary Material 7] to enable rapid creation of games for use across the curriculum, which is of particular benefit in the current educational climate. This blueprint contains the key elements from the diabetes VP games, including free text history taking, RNG, and in-game achievement but uses a new presentation of COVID-19 as an exemplar so that it is accessible and relevant to a wider audience of medical educators. The blueprint has been included in this publication to be freely used and adapted by educators who are keen to incorporate Twine into their educational material. We have recently used this blueprint to rapidly develop games for a postgraduate, multidisciplinary antimicrobial ward round across a local health board.

## Conclusions

Our Twine VP games were well-received by students and, as part of a package of online resources, were associated with significant increases in confidence and knowledge in diabetes acute care and may increase engagement in asynchronously accessed online material. The VP games would appeal to our students later in the curriculum and after lectures, after baseline clinical knowledge has been obtained. This is consistent with cognitive load theory, and it may be sensible to advise on microlecture viewing before VP game utilisation. A blueprint is now available [Supplementary Material 6 and 7] to enable educators to utilise and further evaluate this resource within their own teaching settings.

## Electronic supplementary material

Below is the link to the electronic supplementary material.

Supplementary Material 1: Virtual Patient Game 1

Supplementary Material 2: Virtual Patient Game 2

Supplementary Material 3: Virtual Patient Game 3

Supplementary Material 4: Multiple Choice Questions and Confidence Questionnaires

Supplementary Material 5: Resources Questionnaires

Supplementary Material 6: Virtual Patient Blueprint Game

Supplementary Material 7: Virtual Patient Blueprint Instructions

Supplementary Material 8: Creative Commons/permissions for Twine Virtual Patient Games

## Data Availability

The datasets used during the current study are available from the corresponding author on reasonable request.
