# Correction: Twine virtual patient games as an online resource for undergraduate diabetes acute care education

**DOI:** 10.1186/s12909-023-04540-6

**Published:** 2023-07-31

**Authors:** Nathaniel Patrick Andrew Quail, James Graham Boyle

**Affiliations:** 1 University of Glasgow, Glasgow, G12 8QQ UK; 2 Glasgow Royal Infirmary, Glasgow, G4 0SF UK

**Correction: BMC Medical Education (2023) 23:417**

10.1186/s12909-023-04231-2

Following publication of the original article [1], the authors are aware of missing CSS and JavaScript sections related to the patient examination section in the blueprint game (Supplementary Material 6) and associated instructions (Supplementary Material 7). Please contact the corresponding author for updated materials or any other queries relating to the blueprint game and associated documentation.
