# Supplementary material for: Twine virtual patient games as an online resource for undergraduate diabetes acute care education
Source: BMC Med Educ. 2023 Jun 7;23:417. doi: 10.1186/s12909-023-04231-2 (PMC10244842; doi:10.1186/s12909-023-04231-2)
Supplement: Supplementary file 1 — Supplementary Material 1: Virtual Patient Game 1 [file 12909_2023_4231_MOESM1_ESM.html]

Virtual Patient One


JavaScript must be enabled to play.

Browser lacks capabilities required to play.

Upgrade or switch to another browser.

Loading…

 Just a few quick notes before you start:
\*If the question is multi-choice, make sure you finalise your answers before ticking the box. Some multi-choice questions require more than one answer.
\*For most questions you will be scored on how you answer. If you pick more choices than are necessary (for example, requesting unnecessary blood tests) then you may lose points.
\*Click on the coloured links within passages to continue
\*''Watch out for your web-browser trying to autocorrect free-text answers''
[[Introduction]]
<<set $now to new Date()>>
<<set $start = $now>>
You are an FY2 working in a busy medical receiving department. It's so busy in fact that your name has been forgotten...
Remind me again, Doctor...<<textbox "$doctor" "Surname">>
Ok, ready for your [[next case->John]]
<<audio "backgroundhospitalhypo" volume 0.05 loop play>><<if $history1 eq 0 and $history2 eq 0 and $history3 eq 0>>Dr $doctor, this is John Irvine. He was brought in by his date because after he started to act "strange". \_\_A fairly strong smell of alcohol is noted\_\_.
You ask him why he's been brought in:
<video src="videos/presentingcomplaint2.mp4" width="640" height="480" controls></video>
Nurse Boyle begins routine observations while you take a quick history:<</if>>
<<if $history1 eq 0>>[[Ask about any prescribed medication he is taking]] <</if>>
<<if $history2 eq 0>>[[Ask if he has taken any drugs/alcohol]] <</if>>
<<if $history3 eq 0>>[[Ask if you can speak to whoever brought him in, to take a collateral history]] <</if>>
<<if $history1 eq 1 and $history2 eq 1 and $history3 eq 1>> [[Ask about his past medical history]] <</if>><video src="videos/outwithsomeoneangry.mp4" width="640" height="480" controls></video>
[[Continue history->John]]
<<set $history3 += 1>><video src="videos/nodrugsangry.mp4" width="640" height="480" controls></video>
[[Continue history->John]]
<<set $history2 += 1>><video src="videos/medication.mp4" width="640" height="480" controls></video>
[[Continue history->John]]
<<set $history1 =+ 1>>
You assess John in an A-E manner.
\_\_A\_\_
\* Patent
\_\_B\_\_
\* Respiratory rate - 12 breaths per minute
\* Sats - 99% on air
\* No abnormalities on examination of B
\_\_C\_\_
\* Heart rate - 117 BPM
\* Pulse - regular
\* Blood pressure - 117/76 mm/Hg
\* Heart sounds I+II+0
\_\_D\_\_
With more firm prompting, John's best GCS responses are recorded as follows:
\* John will open his eyes to speech
\* John is able to follow commands
\* John is confused
\_\_E\_\_
\* Nil of note
What are your differential diagnoses?
<<textbox "$differentials1" "">>
<<textbox "$differentials2" "">>
<<textbox "$differentials3" "">>
What is John's GCS?
1 <<radiobutton "$GCS" "wrong">> 2 <<radiobutton "$GCS" "wrong">> 3 <<radiobutton "$GCS" "wrong">>
4 <<radiobutton "$GCS" "wrong">> 5 <<radiobutton "$GCS" "wrong">> 6 <<radiobutton "$GCS" "wrong">>
7 <<radiobutton "$GCS" "wrong">> 8 <<radiobutton "$GCS" "wrong">> 9 <<radiobutton "$GCS" "wrong">>
10 <<radiobutton "$GCS" "wrong">> 11 <<radiobutton "$GCS" "wrong">> 12 <<radiobutton "$GCS" "wrong">>
13 <<radiobutton "$GCS" "correct">> 14 <<radiobutton "$GCS" "wrong">> 15 <<radiobutton "$GCS" "wrong">>
[[Check]]
<<if $GCS eq "wrong">> Incorrect - John opened his eyes to speech (E - 3/4), is confused (V - 4/5), and is able to follow motor commands (M - 6/6) => GCS = 13 <</if>> <<if $GCS eq "correct">> Well done Dr $doctor - John opened his eyes to speech (E - 3/4), is confused (V - 4/5), and is able to follow motor commands (M - 6/6) => GCS = 13 <<set $GCSscore +=1>> <</if>>
<img src="pictures/GCS.png" width="600" height="500" alt="GCS">
The emergency department is extremely busy and Christopher, your fellow FY2, requests your help to clear a backlog of patients from minors.
[[Help your colleague in minors, allowing John time to sober up before reassessing->no]]
OR
[[Further assess John's condition]]
<<set $BM to $BM.toLowerCase()>>
<<if $BM .includes ("bm") or $BM .includes ("gluc") or $BM .includes ("sugar") >> Correct - Blood glucose! John's reading is <span class="yellowtext">2.5mmol/L</span>. His medical notes have been brought up by a colleague - he was diagnosed with type 1 diabetes several years ago. <<set $BMscore = 2>>
John's gag reflex is clearly intact. He has refused glucose tablets. What would be the next most appropriate option to try for John's hypoglycaemia, assuming there are no concerns for staff safety?
100ml Lucozade original orally <<radiobutton "$treatment" "wrong">>
4-5 Glucotabs orally <<radiobutton "$treatment" "wrong">>
1.5 - 2 tubes of Glucogel buccally <<radiobutton "$treatment" "correct">>
1mg IM glucagon <<radiobutton "$treatment" "wrong">>
100ml 5% glucose IV <<radiobutton "$treatment" "wrong">>
[[Treat]]
<<else>>
Not quite - Nurse Boyle gives you several options:
Urine dipstick <<radiobutton "$investigation" "wrong">>
Capillary glucose <<radiobutton "$investigation" "correct">>
Alcohol level <<radiobutton "$investigation" "wrong">>
Arterial blood gas <<radiobutton "$investigation" "wrong">>
Venous blood gas <<radiobutton "$investigation" "wrong">>
[[Correct?]] <</if>>
<<nobr>>
<<if $investigation neq "correct">>
<<set $end to new Date()>>
<<set $duration = (($end-$start)/1000)>>
<<if ($duration < 300)>>
[[try again->Ask Nurse Boyle]] <<set $BMscore = 0>>
<<else>>
You have spent too much time over this and John has [[deteriorated further->no]] <<set $timedeterioration = 1>>
<</if>>
<</if>>
<</nobr>>
<<if $investigation eq "correct">> Correct - Blood glucose! John's reading is <span class="yellowtext">2.5mmol/L</span>. His medical notes have been brought up by a colleague - he has type 1 diabetes.
John's gag reflex is clearly intact. He has refused glucose tablets. What would be the next most appropriate option to treat John's hypoglycaemia?
100ml Lucozade original orally <<radiobutton "$treatment" "wrong">>
A piece of toast <<radiobutton "$treatment" "wrong">>
1.5 - 2 tubes of Glucogel buccally <<radiobutton "$treatment" "correct">>
5mg IM glucagon <<radiobutton "$treatment" "wrong">>
100ml 5% glucose IV <<radiobutton "$treatment" "wrong">>
[[Treat]]
<<set $BMscore = 1>>
<<set $checkedbm +=1>>
<</if>>
<<if $treatment eq "correct">> Well done! <<set $treatmentscore += 1>><</if>> <<if $treatment neq "correct">> Not quite! <</if>> This is a moderate hypoglycaemic episode (cognitive impairment but still able to safely swallow). If the patient is cooperative, they can be encouraged to have 20g of fast-acting carbohydrate (for example, Glucotabs). If they are refusing tablets, the next step to try would be Glucogel. This could be squeezed directly into their mouth. If staff are struggling with this, then more invasive methods, such as Glucagon, would be considered. If this was a case of severe hypoglycaemia (unconscious patient/fitting/nil-by-mouth) then IV glucose (with the aim of giving approximately 20g of glucose in the bag - for example 100ml of 20% glucose) or 1mg IM glucagon would be given. Glucogel and other forms of oral glucose would not be given when there is an unsafe swallow due to the risk of aspiration.
<video src="videos/hypoupdated.mp4" width="640" height="480" controls></video>
When should the capillary glucose be checked again?
Immediately on commencing treatment <<radiobutton "$check" "immediate">>
After 10-15 minutes <<radiobutton "$check" "correct">>
After 30-45 minutes <<radiobutton "$check" "wrong">>
After 1-2 hours <<radiobutton "$check" "wrong">>
After 4-6 hours <<radiobutton "$check" "wrong">>
[[Check blood glucose after specified period of time]]
<<if $check eq "immediate">> There is no need to check the blood glucose immediately after treatment as there will be a negligible change in measurement. Nurse Boyle suggests you check after 10-15 minutes instead to ensure the treatment has worked and John is not still hypoglycaemic.<</if>> <<if $check eq "wrong">> Nurse Boyle suggests you check after 10-15 minutes instead to ensure the treatment has worked and John is not still hypoglycaemic. <</if>> <<if $check eq "correct">> Well done Dr $doctor, correct! <<set $timescore += 1>> <</if>> John's capillary glucose reading 15 minutes after treatment is 4.1 mmol/L.
What should be done now?
Commence an infusion of 100ml 20% glucose <<radiobutton "$carbs" "wrong">>
Encourage John to eat 20g of fast-acting carbohydrate and do not omit his next insulin dose <<radiobutton "$carbs" "wrong">>
Encourage John to eat 20g of fast-acting carbohydrate and omit his next dose of insulin <<radiobutton "$carbs" "wrong">>
Encourage John to eat 20g of long-acting carbohydrate and do not omit his next insulin dose <<radiobutton "$carbs" "correct">>
Encourage John to eat 20g of long-acting carbohydrate and omit his next insulin dose <<radiobutton "$carbs" "wrong">>
[[Continue]]Christopher is becoming impatient and wants to know if there is really anything more to assess...
[[There isn't, help out in minors->no]]
OR
[[Further assess John's condition->2]]
Name the one test that is necessary to perform at this point:
<<textbox "$BM" "">>
[[Ask Nurse Boyle]]<<if $timedeterioration eq "0">>You spend a while helping to clear the backlog in minors. You are then called back to see John, his condition having deteriorated.<</if>>
<video src="videos/GCS3.mp4" width="640" height="480" controls></video>
You assess him in an A-E fashion:
\_\_A\_\_
\* Snoring noise - you correct this with an airway adjunct
\_\_B\_\_
\* Respiratory rate - 10 breaths per minute
\* Sats - 98% on air
\* No abnormalities on examination of B
\_\_C\_\_
\* Heart rate - 131 BPM
\* Pulse - regular
\* Blood pressure - 114/70 mm/Hg
\* Heart sounds I+II+0
\_\_D\_\_
\* John will not open his eyes to pain
\* John will withdraw from pain
\* John has no verbal response
\_\_E\_\_
\* Nothing of note
Nurse Boyle performs a capillary glucose test, which reads <span class="redtext">1.7mmol/L</span>. How should you proceed?
100ml Lucozade original orally <<radiobutton "$treatment2" "wrong">>
4-5 Glucotabs orally <<radiobutton "$treatment2" "wrong">>
1.5 - 2 tubes of Glucogel orally <<radiobutton "$treatment2" "wrong">>
1mg IM glucagon <<radiobutton "$treatment2" "correct">>
100ml 5% glucose IV <<radiobutton "$treatment2" "wrong">>
[[treat?->treatment 2]]<<if $treatment2 eq "correct">> Well done Dr $doctor - 1mg IM glucagon is the best option.
\*\*Glucogel and other forms of oral glucose tend not to be used in unconscious patients due to the risk of aspiration.
\*\*The option of IV glucose suggested would only provide 5g of glucose (5% of 100). Normally 15-20g is given (for example, ''100ml of 20% glucose'').
<<set $treatmentscore += 1>> <</if>> <<if $treatment2 neq "correct">> Not quite Dr $doctor - 1mg IM glucagon is the correct treatment in this instance.
\*\*Glucogel and other forms of oral glucose tend not to be used in unconscious patients due to the risk of aspiration.
\*\*The option of IV glucose suggested would only provide 5g of glucose (5% of 100). Normally 15-20g is given (for example, ''100ml of 20% glucose'').
<</if>> Below is a short video that explains the current treatment algorithm for hypoglycaemia, and how patients tend to present:
<video src="videos/hypoupdated.mp4" width="640" height="480" controls></video>
When should the capillary glucose be checked again?
Immediately on commencing treatment <<radiobutton "$check2" "immediate">>
After 10-15 minutes <<radiobutton "$check2" "correct">>
After 30-45 minutes <<radiobutton "$check2" "wrong">>
After 1-2 hours <<radiobutton "$check2" "wrong">>
After 4-6 hours <<radiobutton "$check2" "wrong">>
[[check?->check2]]<<if $check2 eq "immediate">> There is no need to check the blood glucose immediately after treatment as there will be a negligible change in measurement. Nurse Boyle suggests you check after 10-15 minutes instead to ensure the treatment has worked and John is not still hypoglycaemic.<</if>> <<if $check2 neq "correct">> Nurse Boyle suggests you check after 10-15 minutes instead to ensure the treatment has worked and John is not still hypoglycaemic. <</if>> <<if $check2 eq "correct">> Well done Dr $doctor, correct! <<set $timescore += 1>> <</if>> John's capillary glucose reading 10 minutes after treatment is 5.1 mmol/L and his GCS has improved to 14 (he is still slightly confused but you expect he just needs time to re-orientate himself).
What should be done now?
Commence an infusion of 100ml 20% glucose <<radiobutton "$carbs" "wrong">>
Encourage John to eat 20g of fast-acting carbohydrate and do not omit his next insulin dose <<radiobutton "$carbs" "wrong">>
Encourage John to eat 40g of fast-acting carbohydrate and omit his next dose of insulin <<radiobutton "$carbs" "wrong">>
Encourage John to eat 40g of long-acting carbohydrate and do not omit his next insulin dose <<radiobutton "$carbs" "correct">>
Encourage John to eat 20g of long-acting carbohydrate and omit his next insulin dose <<radiobutton "$carbs" "wrong">>
[[Continue]]<<if $carbs eq "correct">> <<set $carbscore += 1>> Well done Dr $doctor - ''20g'' of long-acting carbohydrate (e.g. a slice of bread) should normally be given after blood glucose is >4mmol/L in order to maintain this level. When glucagon is given, ''40g'' is needed in order to replenish glycogen reserves. Subsequent insulin doses may be altered, but should not be omitted altogether. <</if>> <<if $carbs neq "correct">> Not quite, Dr $doctor - ''20g'' of long-acting carbohydrate (e.g. a slice of bread) should normally be given after blood glucose is >4mmol/L in order to maintain this level. When glucagon is given, ''40g'' is needed in order to replenish glycogen reserves. Subsequent insulin doses may be altered, but should not be omitted altogether. <</if>>
You ask John about the events of the evening:
<video src="videos/reasonforhypo.mp4" width="640" height="480" controls></video>
He admits he had a bit to drink. Which process might alcohol metabolism inhibit, preventing the liver from transforming non-carbohydrate substrates to glucose?
<<textbox "$gluconeogenesis" "">>
[[Check a reputable internet source]]<<set $gluconeogenesis to $gluconeogenesis.toLowerCase()>>
<<if $gluconeogenesis .includes("neogen")>> Well done Dr $doctor - the metabolism of alcohol influences the intracellular redox state of hepatocytes, inhibiting key stages of gluconeogensis. This increases the chance, and severity of, hypoglycaemia. <<set $gluconeogenesisanswer += 1>> <<else>> Not quite, Dr $doctor - the metabolism of alcohol influences the intracellular redox state of hepatocytes, inhibiting key stages of gluconeogenesis. This increases the chance, and severity of, hypoglycaemia. <</if>>
You ask John about the symptoms he experiences when he is hypoglycaemic, and if he can recognise an episode coming on:
<video src="videos/hypoawareness.mp4" width="640" height="480" controls></video>
If a pattern of hypoglycaemia is noticed, it is important to adjust the insulin dose that \*\*leads to\*\* the episodes. For example, if John had problems with frequent hypoglycaemic episodes in the morning, then his evening insulin should be reviewed to stop this.
This is John's ''first hypoglycaemic episode'' requiring medical intervention in several years. He is worried that you are going to tell him he must stop driving, as he uses his car to get to and from work. What is your advice? https://www.gov.uk/guidance/diabetes-mellitus-assessing-fitness-to-drive
He must inform the DVLA and stop driving immediately <<radiobutton "$driving" "wrong">>
He must inform the DVLA but can continue to drive, as long as he has no more episodes of severe hypoglycaemia in the next year <<radiobutton "$driving" "correct">>
He does not need to inform the DVLA and can continue to drive, as long as he has no more episodes of severe hypoglycaemia in the next year <<radiobutton "$driving" "wrong">>
[[Give advice ->You double check the DVLA guidelines]]<video src="videos/seendiabeticteamdrivingetc.mp4" width="640" height="480" controls></video>
A follow-up appointment is arranged with the diabetic team and John is on his way home!
Your clinical supervisor would like to discuss the case with you - let's see [[how you did]]!\_\_What we've covered\_\_
\*Recognising hypoglycaemia
\*Initial treatment of hypoglycaemia
\*Follow-up management of hypoglycaemia - for example, asking about warning signs of hypoglycaemia and assessing fitness to drive
Breakdown of scores:
GCS - $GCSscore / 1
Diagnosis - $BMscore / 2
Treatment - $treatmentscore / 1
Re-assessing Blood Glucose - $timescore / 1
Follow-up treatment - $carbscore / 1
Role of alcohol in hypoglycaemia - $gluconeogenesisanswer / 1
Driving Advice - $drivingscore / 1
Total - <<print ($GCSscore + $BMscore + $treatmentscore + $timescore + $carbscore + $gluconeogenesisanswer + $drivingscore)>> / 8
[[Continue to awards...]]
<<audio "backgroundhospitalhypo" stop>>
<<audio "sugarhypo" volume 0.5 loop play>><<if $GCSscore eq "1">> <img src="pictures/trophyhypo.png" width="60" height="80" alt="trophyhypo"> Congratulations - you've earned the ''GCS Award''. That's the ''Glasgow'' Coma Scale. Glasgow's awesome. We're the filling of an Oxbridge sandwich for medical school rankings you know (correct as of March 2018 - probably fallen dramatically since - I'm not going to update this). <</if>> <<if $GCSscore neq "1">> <img src="pictures/notrophyhypo.png" width="60" height="80" alt="trophy"> Try playing through again to unlock this award. <</if>>
<<if $BMscore eq "2">> <img src="pictures/trophyhypo.png" width="60" height="80" alt="trophyhypo"> Congratulations - you've earned the ''Dr House Award'' for diagnostic skills! Ok, it might not have been lupus, but you've made an important diagnosis so well done you! Remember, don't ever forget glucose! <</if>> <<if $BMscore neq "2">> <img src="pictures/notrophyhypo.png" width="60" height="80" alt="trophy"> Try playing through again to unlock this award. <</if>>
<<if $gluconeogenesisanswer eq "1">> <img src="pictures/trophyhypo.png" width="60" height="80" alt="trophyhypo"> Congratulations - you've earned the ''MSN Messenger Award'' for excellence in biochemistry! ...what? It's the MSN Messenger Award because the process of gluconeogenesis used to be an important part of everyone's life, but now only a couple of keen beans - like yourself - really still think about it. You're nostalgic for those carefree pre-clinical years, aren't you?<</if>> <<if $gluconeogenesisanswer neq "1">> <img src="pictures/notrophyhypo.png" width="60" height="80" alt="trophy"> Try playing through again to unlock this award. <</if>>
Thanks for playing the game!
[[Restart]]<video src="videos/ivehad-.mp4" width="640" height="480" controls></video>
[[You assess John from A-E]]<<cacheaudio "backgroundhospitalhypo" "music/backgroundhospitalhypo.mp3">>
<<cacheaudio "sugarhypo" "music/sugarhypo.mp3">>
<<set $checkedbm = 0>>
<<set $history1 = 0>>
<<set $history2 = 0>>
<<set $history3 = 0>>
<<set $BMscore = 0>>
<<set $gluconeogenesisanswer = 0>>
<<set $GCSscore = 0>>
<<set $treatmentscore = 0>>
<<set $timescore = 0>>
<<set $carbscore = 0>>
<<set $checkedbm = 0>>
<<set $now to new Date()>>
<<set $start = $now>>
<<set $duration = 0>>
<<set $drivingscore = 0>>
<<set $timedeterioration = 0>><<if $driving eq "correct">> Well done Dr $doctor! <<set $drivingscore +=1>> <<else>> Not quite Dr $doctor!<</if>> John has good hypoglycaemic awareness, practises appropriate blood glucose monitoring and (we assume) meets the legal visual requirement. Therefore, a single episode of severe hypoglycaemia, while awake, in the past 12 months should not prevent John from driving his ''car'' (correct as of 2023 - this can change!). He should, however, inform the DVLA of this episode. Always make sure to double check with latest DVLA guidance before discussing with patients.
John is just about ready for discharge, but you would like a member of the diabetes team to go over points on driving and diabetic management in more detail with him [[before he leaves]].Welcome to the <span class="greentext"> Acute Care Virtual Patient Simulation - Scenario 1</span>!
This was developed by Dr Nat Quail under the close supervision of Dr James Boyle\*. We would like to acknowledge the generous grant received from the Learning and Teaching Development Fund, which enabled us to pay for actors and equipment.
We hope that this game will enable you to learn more about the management of specific conditions and acutely ill patients in a fun\*\* and safe manner.
[[Instructions]]
So it's his fault if anything goes rwong\*
May not be fun\*\*<<script>>
state.restart();
<<endscript>>
