# Supplementary material for: Twine virtual patient games as an online resource for undergraduate diabetes acute care education
Source: BMC Med Educ. 2023 Jun 7;23:417. doi: 10.1186/s12909-023-04231-2 (PMC10244842; doi:10.1186/s12909-023-04231-2)
Supplement: Supplementary file 2 — Supplementary Material 2: Virtual Patient Game 2 [file 12909_2023_4231_MOESM2_ESM.html]

Virtual Patient Two


JavaScript must be enabled to play.

Browser lacks capabilities required to play.

Upgrade or switch to another browser.

Loading…

 You are an FY2 working in a busy medical receiving department. It's busy in fact that your name has been forgotten...
Remind me again, Doctor...<<textbox "$doctor" "name">>
[[Continue->Brenda Rodgers]]
<<audio "backgroundhospital" volume 0.05 loop play>>Dr <<print$doctor>>, this is Brenda Rodgers. She is a 19-year-old female who has presented to A&E feeling generally unwell.
<video src="videos/dob1.mp4" width="640" height="480" controls></video>
You ask her why she’s been brought in:
<video src="videos/presentingcomplaint.mp4" width="640" height="480" controls></video>
You ask Nurse Boyle to run a set of obs while you enquire more about [[Brenda's pain->Pain assessment]]<<if $pastmedicalhistory eq 0 and $socialhistory eq 0 and $familyhistory eq 0 and $bbq eq 0 and $gisym eq 0>> <video src="videos/pain.mp4" width="640" height="480" controls> </video>
Nurse Boyle has finished his obs:
"Ok Doctor <<print $doctor>>, Brenda’s blood pressure is 105/70mmHg, heart rate is 121BPM, respiratory rate is 20 breaths per minute, and her oxygen saturations are 100% on room air. She is apyrexial."
You think she is quite unwell but would like a bit more history before you continue with your physical assessment. <</if>>
Would you like to:
<<actions
[[Ask Brenda about GI symptoms]]
[[Ask about past medical history/medications]]
[[Ask about family history]]
[[Ask about social history]]>>
<<if $pastmedicalhistory gt 0 and $socialhistory gt 0 and $familyhistory gt 0>>[[Consider your differentials?->What are your initial differentials?]]<<endif>>
<video src="videos/bbq.mp4" width="640" height="480" controls> </video>
Brenda tells you she has been sick but has not thrown up bile or blood. You might have time to ask about a couple of other specific GI symptoms to give you a better idea if this is more of a ''gastroenteritis'' presentation...
<<textbox "$blood" "">>
<<textbox "$diarrhoea" "">>
[[Ask away...]]
<<set $gisym = 1>><video src="videos/thrush.mp4" width="640" height="480" controls> </video>
[[Back to questions->Pain assessment]]
<<remember $pastmedicalhistory += 1>><video src="videos/fh.mp4" width="640" height="480" controls> </video>
Interesting [[Back to questions->Pain assessment]]
<<remember $familyhistory += 1>><video src="videos/liveathome.mp4" width="640" height="480" controls> </video>
Brenda also states that she does not smoke and drinks socially. She does not have a part-time job.
[[Back to questions->Pain assessment]]
<<remember $socialhistory += 1>>First time cannulation! You're getting good at this...
What bloods do you want and why? Remember to tick the box and give a reason - try not to order any unnecessary tests!
FBC <<checkbox "$FBC" false true unchecked>> <<textbox "$FBCreason" "reason?">>
U&E's <<checkbox "$UE" false true unchecked>> <<textbox "$UEreason" "reason?">>
LFT's <<checkbox "$LFT" false true unchecked>> <<textbox "$LFTreason" "reason?">>
Formal glucose <<checkbox "$labglucose" false true unchecked>> <<textbox "$labglucosereason" "reason?">>
Insulin <<checkbox "$Insulin" false true unchecked>> <<textbox "$Insulinreason" "reason?">>
Blood cultures <<checkbox "$bloodcultures" false true unchecked>> <<textbox "$bloodculturesreason" "reason?">>
CRP <<checkbox "$CRP" false true unchecked>> <<textbox "$CRPreason" "reason?">>
Arterial blood gas <<checkbox "$ABG" false true unchecked>> <<textbox "$ABGreason" "reason?">>
Venous blood gas <<checkbox "$VBG" false true unchecked>> <<textbox "$VBGreason" "reason?">>
[[Send off samples]]
<<audio "heartsounds" stop>>
<<audio "backgroundhospital" volume 0.05 loop play>>Your senior, Dr Martin, explains what [[bloods he expected]] you to perform and why - let's compare your reasoning and add on any tests that you missed!
Here is your latest list of differentials:
<span class="greentext"> $differentials21 </span>
<span class="greentext"> $differentials22 </span>
What is your diagnosis? <<textbox "$answer" "">>
Why? <<textbox "$reasondiagnosis" "">>
[[Answer]]<<if $answer eq "DKA" or $answer eq "Diabetic ketoacidosis" or $answer eq "Dka" or $answer eq "diabetic ketoacidosis" or $answer eq "Diabetic Ketoacidosis" or $answer eq "ska" or $answer eq "aka" or $answer eq "dka" or $answer eq "diabetic keto-acidosis" or $answer eq "Diabetic Keto-acidosis" or $answer eq "Diabetic Keto-Acidosis">><span class="greentext">Correct!</span> [[your next steps]]... <<set $total += 2>> <<set $rightdiagnosis += 2>> <</if>>
<<if $answer neq "DKA" and $answer neq "Diabetic ketoacidosis" and $answer neq "Dka" and $answer neq "diabetic ketoacidosis" and $answer neq "aka" and $answer neq "ska" and $answer neq "Diabetic Ketoacidosis" and $answer neq "dka" and $answer neq "diabetic keto-acidosis" and $answer neq "Diabetic Keto-acidosis" and $answer neq "Diabetic Keto-Acidosis">> Not quite...[[Multiple Choice]]? <</if>>Pick one:
Hyperosmolar Hyperglycaemic State <<radiobutton "$dkamc" "wrong">>
Gastroenteritis <<radiobutton "$dkamc" "wrong">>
Ectopic Pregnancy <<radiobutton "$dkamc" "wrong">>
Diabetic Ketoacidosis <<radiobutton "$dkamc" "correct">>
Starvation Ketosis <<radiobutton "$dkamc" "wrong">>
[[Right answer?]]
Dr Martin explains that Brenda is likely to be in DKA because of her <span class="redtext">hyperglycaemia, high levels of ketones, and acidosis</span>.
Your reason was: <span class="greentext"> $reasondiagnosis </span>
Brenda could have gastroenteritis, which may have precipitated this, but this is not the main diagnosis.
Brenda initially informed us that she had lost weight, was being sick, and had crampy abdominal pain.<<if ($polyuria + $polydipsia + $nocturia + $polyphagia + $weight) gt 0>> In relation to a new presentation of diabetes, you also asked her specifically about:
<<if $polyuria neq 0>>\*Polyuria<</if>>
<<if $polydipsia neq 0>>\*Polydipsia<</if>>
<<if $polyphagia neq 0>>\*Polyphagia<</if>>
<<if $nocturia neq 0>>\*Nocturia <</if>>
<<if $weight neq 0>>\*Weight loss <</if>> <</if>>
<<if ($polyuria + $polydipsia + $nocturia + $polyphagia + $weight) lt 5>> The following symptoms might also have given you a clue that this was a new presentation of diabetes:
<<if $polyuria eq 0>>\*Polyuria<</if>>
<<if $polydipsia eq 0>>\*Polydipsia<</if>>
<<if $polyphagia eq 0>>\*Polyphagia<</if>>
<<if $nocturia eq 0>>\*Nocturia <</if>>
<<if $weight eq 0>>\*Weight loss <</if>> <</if>>
Brenda presented with <span class="greentext">weight loss, nausea and vomiting, and cramping abdominal pain</span>. Weight loss can be a sign of new onset type 1 diabetes. Nausea and vomiting, and cramping abdominal pain, can importantly be symptoms of <span class="redtext">diabetic ketoacidosis</span>. <span class="greentext">Polyuria, polydipsia, polyphagia, and nocturia</span> are further symptoms that might indicate diabetes when found in the history. On examination, deep breathing - <span class="greentext">Kussmaul breathing</span> - indicates the patient is trying to compensate for their metabolic acidosis by blowing off CO2. Other findings might also include <span class="greentext"> a fruity breath (due to ketones), dehydration, tachycardia, and hypotension</span>.
Ectopic pregnancy is always a differential to consider in a young woman presenting with abdominal pain but the HCG test was negative and cramping abdominal pain can be present in DKA. Hyperosmolar hyperglycaemic state tends to occur in older people with type 2 diabetes, and is usually associated with even higher blood glucose readings than in Brenda's case (and usually occurs without acidosis due to a relative rather than absolute insulin defficieny). Other differentials, such as starvation ketosis, are less likely as she is hyperglycaemic.
Here is a short chalk-talk video on the difference between DKA and HHS, two similar diabetic emergencies:
<video src="videos/dkatwine18\_12.mp4" width="640" height="480" controls></video>
Dr Martin asks you what has happened to the patient's anion gap with this metabolic acidosis.
<span class="greentext"> Sodium 146mmol/L, Potassium 5.1mmol/L, Chloride 101mmol/L, Bicarbonate 11mmol/L, (assume normal albumin) </span>
The anion gap is reduced overall due to the net potassium efflux from cells <<radiobutton "$aniongap" "wrong">>
The anion gap is reduced overall due to the presence of ketones <<radiobutton "$aniongap" "wrong">>
The anion gap is unchanged since Brenda's albumin is within a normal range <<radiobutton "$aniongap" "wrong">>
The anion gap is increased overall due to the net potassium efflux from cells <<radiobutton "$aniongap" "wrong">>
The anion gap is increased overall due to the presence of ketones <<radiobutton "$aniongap" "correct">>
[[Ready?->findout]]
<<audio "backgroundhospital" stop>>
You now intend on assessing Brenda in an A-E manner. Nurse Boyle is arranging an ECG and asks you what the 3 most important other bedside tests he should now perform are, given Brenda's presentation?
''Use a single word or an acronym in each box and do not use punctuation or quotation marks''
<<textbox "$BM" "">>
<<textbox "$DS" "">>
<<textbox "$HCG" "">>
[[Let's see...]]<<set $BM to $BM.toLowerCase()>> <<set $DS to $DS.toLowerCase()>> <<set $HCG to $HCG.toLowerCase()>> <<set $BM to $BM.trim()>> <<set $DS to $DS.trim()>> <<set $HCG to $HCG.trim()>>
<<if $BM .includes ("bm") or $BM .includes ("glucose")>>Correct - Blood glucose! Brenda's is 26mmol/L.<<set $BManswer = 1>> <</if>> <<if $HCG .includes ("bm") or $HCG .includes ("glucose")>>Correct - Blood glucose! Brenda's is 26mmol/L.<<set $BManswer = 1>> <</if>> <<if $DS .includes ("bm") or $DS .includes ("glucose")>>Correct - Blood glucose! Brenda's is 26mmol/L. <<set $BManswer = 1>> <</if>> <<if $BM .includes ("dip") or $BM .includes ("urin")>>Correct - Urine dipstick! Brenda's is 4+ glucose and 3+ ketones.<<set $DSanswer = 1>> <</if>> <<if $HCG .includes ("dip") or $HCG .includes ("urin")>>Correct - Urine dipstick! Brenda's is 4+ glucose and 3+ ketones.<<set $DSanswer = 1>> <</if>> <<if $DS .includes ("dip") or $DS .includes ("urin")>>Correct - Urine dipstick! Brenda's is 4+ glucose and 3+ ketones.<<set $DSanswer = 1>> <</if>> <<if $BM .includes ("hcg") or $BM .includes ("human") or $BM .includes ("pregnan")>> Correct - HCG! Brenda's is negative.<<set $HCGanswer = 1>> <</if>> <<if $HCG .includes ("hcg") or $HCG .includes ("human") or $HCG .includes ("pregnan")>> Correct - HCG! Brenda's is negative. <<set $HCGanswer = 1>> <</if>> <<if $DS .includes ("hcg") or $DS .includes ("human") or $DS .includes ("pregnan")>> Correct - HCG! Brenda's is negative. <<set $HCGanswer = 1>> <</if>> <<if $BM .includes ("ketone")>>Correct - Capillary ketones! The result is 4.1mmol/L.<<set $ketoneanswer = 1>> <</if>> <<if $DS .includes ("ketone")>>Correct - Capillary ketones! The result is 4.1mmol/L.<<set $ketoneanswer = 1>> <</if>> <<if $HCG .includes ("ketone")>>Correct - Capillary ketones! The result is 4.1mmol/L.<<set $ketoneanswer += 1>> <</if>>
<<if $BManswer eq 1>> <<set $pointofcare + 1>> <</if>>
[[Continue->Feedback]]
<<if ($HCGanswer + $BManswer + $DSanswer + $ketoneanswer) eq 3>> Congratulations Doctor <<print$doctor>>, you thought of 3 important bedside tests!<</if>> <<if $BManswer eq 0>> Nurse Boyle would also like to perform a capillary blood glucose. It comes back as 26mmol/L.<</if>> <<if $DSanswer eq 0>> Nurse Boyle would also like to perform a urinary dipstick. The result is 4+ glucose and 3+ ketones.<</if>> <<if $HCGanswer eq 0>> Nurse Boyle would also like to perform a urinary HCG (with Brenda's consent) due to her age and abdominal pain. It is negative.<</if>> <<if $ketoneanswer eq 0>> Nurse Boyle would also like to check for capillary ketones - the result is 4.1mmol/L.<</if>>
Your initial differentials were:
<span class="greentext"> $differentials1 </span>
<span class="greentext"> $differentials2 </span>
<span class="greentext"> $differentials3 </span>
What are your differentials now?
Most likely? Why? <<textbox "$differentials21" "">>
Second most likely? Why? <<textbox "$differentials22" "">>
Continue to [[A to E assessment->Breathing]]
<<if $DSanswer eq 1>> <<set $pointofcare += 1>> <</if>>
<<if $BManswer eq 1>> <<set $pointofcare += 1>> <</if>>
<<if $HCGanswer eq 1>> <<set $pointofcare += 1>> <</if>>
<<if $ketoneanswer eq 1>> <<set $pointofcare += 1>> <</if>><<if $dkamc eq "correct">> Correct! [[your next steps]] <<set $total += 1>> <<set $rightdiagnosis += 1>> <</if>>
<<if $dkamc neq "correct">> [[Try again!->Multiple Choice]]<</if>>
<<cacheaudio "breathsounds" "music/breathsounds.mp3">>
<<cacheaudio "heartsounds" "music/heartsounds.mp3">>
<<cacheaudio "backgroundhospital" "music/backgroundhospital.mp3">>
<<cacheaudio "alarm" "music/alarm.mp3">>
<<cacheaudio "sugar" "music/sugar.mp3">>
<<set $triad = 0>>
<<set $pointofcare = 0>>
<<set $goodblood = 0>>
<<set $badblood = 0>>
<<set $rightdiagnosis = 0>>
<<set $anionquestion = 0>>
<<set $firstbag = 0>>
<<set $initialinsulin = 0>>
<<set $thrombo = 0>>
<<set $threehours = 0>>
<<set $subcut = 0>>
<<set $kreplacement = 0>>
<<set $polydipsia = 0>>
<<set $polyuria = 0>>
<<set $polyphagia = 0>>
<<set $nocturia = 0>>
<<set $weight = 0>>
<<set $bloodanswer = 0>>
<<set $diarrhoeaanswer = 0>>
<<set $pastmedicalhistory = 0>>
<<set $bbq = 0>>
<<set $gisym = 0>>
<<set $familyhistory = 0>>
<<set $socialhistory = 0>>
<<set $total = 0>>
<<set $BManswer = 0>>
<<set $DSanswer = 0>>
<<set $HCGanswer = 0>>
<<set $ketoneanswer = 0>>
<<set $hypo = 0>>You listen to Brenda's chest and hear these breath sounds.
Inspection, expansion, and percusion are all normal. Sats are 100%.
<<audio "breathsounds" loop play>>
You now have a quick listen to Brenda's [[heart sounds->Heart Sounds]]
<<audio "backgroundhospital" stop>>Here are Brenda's heart sounds.
An ECG has also been performed:
<img src="pictures/ecgbrenda.png" width="400" height="200" alt="ecg">
You then have a quick examination of her abdomen, which is unremarkable, before obtaining [[IV access]].
<<audio "breathsounds" stop>>
<<audio "heartsounds" volume 3 loop play>>Given the history, you worry that this might be a first presentation of type 1 diabetes and related complications (let's be honest, it's a Diabetes Acute Care Day Virtual Patient...)
Please write down any symptoms of type 1 diabetes that you would ask Brenda about. ''Keep coming back to this page to ask about as many relevant symptoms as you can think of.'' Since you're writing in the notes, make sure you use the ''medical term''!
<<textbox "$symptom" "">>
[[Ask about symptom]]
<<set $symptomcheck = 0>><<nobr>>
<<set $symptom to $symptom.toLowerCase()>>
<<set $symptom to $symptom.trim()>>
<<if $symptom .includes("polyd") or $symptom .includes("thirst") or $symptom .includes("drink")>><video src="videos/polydipsia.mp4" width="640" height="480" controls> </video>
<<set $polydipsia = 1>>
<<set $symptomcheck = 1>><</if>>
<<if $symptom .includes("polyph") or $symptom .includes("hung") or $symptom .includes("eat") or $symptom .includes("appet")>><video src="videos/polyphagia.mp4" width="640" height="480" controls> </video> <<set $polyphagia = 1>> <<set $symptomcheck = 1>> <</if>>
<<if $symptom .includes("polyur") or $symptom .includes("frequen") or $symptom .includes ("toilet") or $symptom .includes("urin")>><video src="videos/polyuria.mp4" width="640" height="480" controls> </video> <<set $polyuria = 1>> <<set $symptomcheck = 1>> <</if>>
<<if $symptom .includes ("vomit") or $symptom .includes ("naus") or $symptom .includes ("sick")>><video src="videos/nandv.mp4" width="640" height="480" controls> </video> <<set $symptomcheck = 1>> <</if>>
<<if $symptom .includes ("diar") or $symptom .includes ("constipation") or $symptom .includes ("stool")>><video src="videos/diarrhoea.mp4" width="640" height="480" controls> </video> <<set $symptomcheck = 1>> <</if>>
<<if $symptom .includes ("noctu") or $symptom .includes ("night") or $symptom .includes ("bed")>> <video src="videos/nocturia.mp4" width="640" height="480" controls> </video> <<set $nocturia = 1>> <<set $symptomcheck = 1>>
<</if>> <<if $symptom .includes ("weight") or $symptom .includes ("anorexi")>><video src="videos/weight.mp4" width="640" height="480" controls> </video> <<set $weight = 1>> <<set $symptomcheck = 1>> <</if>>
<<if $symptomcheck eq "0">> You aren't asking the right questions Dr $doctor.<</if>>
<</nobr>>
Remember to ask Brenda as much as you can.
[[Ask something else->What specific symptoms do you want to ask about?]]
''Once you have finished taking your history,'' Nurse Boyle has a [[question->Point of Care Tests]] for you...You mentally take note of your initial differential diagnoses in these handy boxes:
Most likely and why? <<textbox "$differentials1" "">>
Second most likely and why? <<textbox "$differentials2" "">>
Third most likely and why? <<textbox "$differentials3" "">>
[[Continue->What specific symptoms do you want to ask about?]]
\_\_ <span class="greentext"> Bloods he wanted </span> \_\_
<<timed 1s>> "I wanted a full blood count in case of anaemia or infection."
<<if $FBC>>Your reason was: <span class="greentext"> \_\_ $FBCreason \_\_</span><</if>>
<<next>> “CRP - ?Infection."
<<if $CRP>>Your reason was: <span class="greentext"> \_\_ $CRPreason \_\_</span><</if>>
<<next>> "VBG - For acid-base balance."
<<if $VBG>>Your reason was: <span class="greentext"> \_\_ $VBGreason \_\_</span><</if>>
<<next>> "U&Es - For electrolytes (especially potassium) and renal function. In some hospitals this will include a formal bicarbonate. If not, you should request this as well."
<<if $UE>>Your reason was: <span class="greentext"> \_\_ $UEreason \_\_</span><</if>>
<<next>> "LFTs - an albumin will be useful if we need to assess Brenda's anion gap."
<<if $LFT>>Your reason was: <span class="greentext"> \_\_ $LFTreason \_\_</span><</if>>
<<next>> "Glucose - point of care glucose only gives up to certain value."
<<if $labglucose>>Your reason was: <span class="greentext"> \_\_ $labglucosereason \_\_</span><</if>>
\_\_ <span class="yellowtext"> Bloods he did not want, but would not be unreasonable to order </span> \_\_
<<next>> "I may have considered blood cultures - ?infectious cause - but the patient is apyrexial and she is only SIRS 1/3 (don't know white cell count) at this point."
<<if $bloodcultures>>Your reason was: <span class="greentext"> \_\_ $bloodculturesreason \_\_</span><</if>>
\_\_ <span class="redtext"> Bloods he did not want </span> \_\_
<<next>> "Insulin levels are not routinely checked in hospitals. C-peptide could be checked as a marker of endogenous insulin production but this would not be requested on admission bloods."
<<next>> "Although an arterial blood gas would give pH and bicarbonate, it is unnecessary as we do not suspect Brenda is hypoxic or hypercapnic (this is much more painful than a VBG!).
<<next>> You add on any bloods you might have missed. Your venous blood gas results are [[as follows->VBG]] <</timed>>
<<if $aniongap eq "correct">> Correct! <<set $anionquestion += 1>> <</if>>
<<if $aniongap neq "correct">> Not quite! <</if>>
Below is a quick chalk-talk video on the anion gap:
<video src="videos/chalktalk2.mp4" width="640" height="480" controls></video>
''Note'' - the reference range for anion gap may vary between source and differ if including or excluding potassium in the calculation. Often, if including potassium, the lower end of the reference is quoted as 12mmol/L rather than 8mmol/L.
In Brenda's case: <span class="greentext"> (5 + 145) - (101 + 11) = 38mmol/L </span> - this is clearly abnormal.
<span class="greentext"> A couple of notes: </span>
-As mentioned, sometimes potassium is ommitted from the equation due to its small value
-Hypoalbuminaemia can mask a high anion gap and so an albumin-corrected anion gap should be calculated
Now let's start [[treating Brenda's DKA]]
''Remember, you are treating Brenda for DKA because she is acidotic. If her H+ and bicarbonate was normal, she would simply have a likely diagnosis of new type 1 diabetic and need subcutaenous insulin''
You don't have a DKA protocol to hand and Dr Martin has told you to start treating Brenda's DKA. Which of the below is the most sensible?
1L NaCl (0.9%) over 1 hour <<radiobutton "$nacl" "correct">>
1L NaCl (0.9%) over 2 hours <<radiobutton "$nacl" "wrong">>
1L dextrose (10%) over 1 hour <<radiobutton "$nacl" "wrong">>
1L dextrose (10%) over 2 hours <<radiobutton "$nacl" "wrong">>
[[Happy?->fluid find out]]
<<audio "backgroundhospital" volume 0.05 loop play>>
<<if $nacl eq "correct">> Dr Martin congratulates you on knowing the DKA pathway like the back of your hand! <<set $total += 1>> <<set $firstbag += 1>> <</if>>
<<if $nacl neq "correct">> Dr Martin suggests running 1L of 0.9% NaCl over an hour. <</if>>
Audits have shown that one of the main errors in managing DKA is the delay in starting initial fluids - although the protocol is normally easily accessible it is useful to know the initial steps for speedy management.
Now Dr Martin asks you to fill out the chart to commence Brenda on IV insulin. The Greater Glasgow and Clyde DKA Pathway has the insulin prescription on the form itself, however let's assume you're working somewhere where this is not the case...
<img src="pictures/insulin scale rubbed.png" width="500" height="300" alt="insulin">
What is the appropriate dilutent and initial infusion rate?
50ml NaCl (0.9%), 6 units per hour <<radiobutton "$naclinsulin" "correct">>
50ml dextrose, 6 units per hour <<radiobutton "$naclinsulin" "wrong">>
50ml NaCl (0.9%), 12 units per hour <<radiobutton "$naclinsulin" "wrong">>
50ml dextrose, 12 units per hour <<radiobutton "$naclinsulin" "wrong">>
Let's [[fill in the prescription chart]]Dr Martin checks over the prescription for a [[counter-signature]]...<<if $naclinsulin eq "correct">> "Well done Doctor <<print$doctor>>" ! Correct! <<set $total += 1>> <<set $initialinsulin += 1>> <</if>>
<<if $naclinsulin neq "correct">> Not quite...Dr Martin corrects the prescription for you. <</if>>
<img src="pictures/insulin scale complete.png" width="500" height="200" alt="insulincomplete">
Dr Martin informs you that if Brenda was already on treatment for her diabetes, then long-acting insulin analogues such as glargine (Lantus) and detemir (Levemir) <span class="greentext">should be continued</span> to help prevent transitional hyperglycaemia when the intravenousinfusion is eventually stopped.
Dr Martin, priding himself on being an excellent teacher, shows you more of the initial DKA pathway but covers up a section. He asks you what management you think he has covered up is and why it is important (one word)...
<img src="pictures/dka management.png" width="500" height="200" alt="dkamanagement">
<<textbox "$management" "">>
You listen intently to his [[answer->dka management]]...<img src="pictures/DKA management 2.png" width="500" height="150" alt="dkamanagement2">
<<set $management to $management.toLowerCase()>> <<if $management .includes ("thromboprophylaxis") or $management .includes ("dvt") or $management .includes ("prophylaxis") or $management .includes ("clexane") or $management .includes ("lmwh") or $management .includes ("enoxaparin")>> <<set $thrombo += 1>> Correct! <<else>> Not quite!<</if>>The management option was <span class="greentext"> thromboprophylaxis </span>. Dr Martin explains that Brenda is at risk of a thrombotic event due to her dehydration and stasis of blood flow. If she had a pre-existing diabetes diagnosis then endothelial dysfunction from advanced glycation end products would further increase her risk of thrombosis.
The other investigations/managements are also important. For example, it is vital to regularly monitor the GCS of children and adolescents as they are at risk of cerebral oedema from treatment.
Thanking Dr Martin for imparting his wisdom, you proceed through the rest of the [[DKA pathway]]...<<set $potassium to random(3)>>
Some of Brenda's blood results are back from the lab. As expected, Brenda is hyperglycaemic with a glucose of 26 mmol/L. Of note, the potassium is: <<if $potassium is 0>>3.7 mmol/L (3.5–5.3)<</if>>
<<if $potassium is 1>>4.2 mmol/L (3.5–5.3)<</if>>
<<if $potassium is 2>>4.6 mmol/L (3.5–5.3)<</if>>
<<if $potassium is 3>>5.1 mmol/L (3.5–5.3)<</if>>
How do these results inform your management?
[[Add potassium to fluids->potassiumadded]]
[[Omit any additional potassium->nopotassium]]
[[Commence calcium gluconate and seek senior advice->gluconate]]<<nobr>>
<<set $blood to $blood.toLowerCase()>>
<<set $diarrhoea to $diarrhoea.toLowerCase()>>
<<if $blood .includes ("blood") or $blood .includes ("hematoch") or $blood .includes ("haematoch") or $blood .includes ("bleed")>><video src="videos/blood.mp4" width="640" height="480" controls> </video> <<set $bloodanswer += 1>> <</if>>
<<if $blood .includes ("diar") or $blood .includes ("constipa") or $blood .includes ("mucous") or $blood .includes ("mucus") or $blood .includes ("loose") or $blood .includes ("change")>><video src="videos/diarrhoea.mp4" width="640" height="480" controls> </video> <<set $diarrhoeaanswer += 1>> <</if>>
<<if $diarrhoea .includes ("diar") or $diarrhoea .includes ("mucus") or $diarrhoea .includes ("mucous") or $diarrhoea .includes ("constipat") or $diarrhoea .includes ("loose") or $diarrhoea .includes ("change")>><video src="videos/diarrhoea.mp4" width="640" height="480" controls> </video> <<set $diarrhoeaanswer += 1>> <</if>>
<<if $diarrhoea .includes ("blood") or $diarrhoea .includes ("hemato") or $diarrhoea .includes ("haemato") or $diarrhoea .includes ("bleed")>><video src="videos/blood.mp4" width="640" height="480" controls> </video> <<set $bloodanswer += 1>><</if>>
<</nobr>>
<<if $diarrhoeaanswer eq 0>> You also ask whether Brenda has been experiencing any diarrhoea - she has not. <</if>>
<<if $bloodanswer eq 0>> You also ask whether Brenda has been passing any blood - she has not. <</if>>
Brenda isn't sure whether anyone else in attendance at the BBQ is ill. Let's ask some more [[history->Pain assessment]]<<if $potassium lt 3>> Correct Doctor <<print $doctor>>! <<set $kreplacement += 1>> <<set $total += 1>> <<else>> Not quite Doctor <<print $doctor>> ! <</if>>
Dr Martin explains that, as with any guideline, it is not important to learn the DKA pathway by heart. However, having a rough understanding of the concepts behind the guideline helps in the pragmatic management of the acutely unwell patient. For example, as previously discussed dextrose should not be used as a resuscitation fluid.
<img src="pictures/potassiumpic.png" width="500" height="300" alt="potassium">
The aim in initial management of DKA is to keep the measured potassium level relatively high (but not too high!). This is because total body stores are actually decreased due to the diuresis - the number appears high because potassium is extracellularised with decreased insulin and the total blood volume is also decreased due to the osmotic diuresis. When treatment is given - fluids and insulin - these factors will be reversed and so hypokalaemia can easily develop. This may in turn have consequences related to cardiac arrhythmias.
[[Some time passes...->glucose<14]]<<if $potassium is 3>> Correct Doctor <<print$doctor>> ! See the explanation below. <<set $kreplacement += 1>> <<else>> Not quite Doctor. <<print$doctor>> !>> <</if>>
Dr Martin explains that, as with any guideline, it is not important to learn the DKA pathway by heart. However, having a rough understanding of the concepts behind the guideline helps in the pragmatic management of the acutely unwell patient.
<img src="pictures/potassiumpic.png" width="500" height="300" alt="potassium">
The aim in initial management of DKA is to keep the measured potassium level relatively high (but not too high!). This is because total body stores are actually decreased due to the diuresis - the number appears high because potassium is extracellularised with decreased insulin and the total blood volume is also decreased due to the diuresis. When treatment is given - fluids and insulin - these factors will be reversed and so hypokalaemia can easily develop. This may lead to cardiac arrhythmias.
[[Some time passes...->glucose<14]]Not quite Doctor <<print $doctor>> !
Dr Martin explains that, as with any guideline, it is not important to learn the DKA pathway by heart. However, having a rough understanding of the concepts behind the guideline helps in the pragmatic management of the acutely unwell patient. For example, as previously discussed dextrose should not be used as a resuscitation fluid.
<img src="pictures/potassiumpic.png" width="500" height="300" alt="potassium">
The aim in initial management of DKA is to keep the measured potassium level relatively high (but not too high!). This is because total body stores are actually decreased due to the diuresis - the number appears high because potassium is extracellularised with decreased insulin and the total blood volume is also decreased due to the diuresis. When treatment is given - fluids and insulin - these factors will be reversed and so hypokalaemia can easily develop. This may in turn have consequences related to cardiac arrhythmias.
[[Some time passes...->glucose<14]]Brenda's glucose is now 13mmol/L, 3 hours after admission. Ailsa, a healthcare assistant who has checked Brenda's capillary glucose, is worried about her becoming hypoglycaemic. Brena's potassium was last measured as 4.1mmol/L (3.5–4.9). How do you proceed?
[[Do nothing, Brenda is still hyperglycaemic->1]]
[[Reduce her insulin and add potassium to her fluids->2]]
[[Stop her insulin and add potassium to her fluids->3]]
[[Prescribe dextrose with potassium ->4]]
[[Prescribe dextrose with potassium and reduce her insulin->5]]
[[Prescribe dextrose with potassium and stop her insulin temporarily->6]]After a busy morning you now feel like you've earned your lunch. You have a leisurely hour eating food that could only be rivalled by Wishaw's cafeteria.
<img src="pictures/lunch.png" width="500" height="300" alt="lunch">
<<set $hypo to 1>>
[[Return to the ward]]After a busy morning you now feel like you've earned your lunch. You have a leisurely hour eating food that could only be rivalled by Wishaw's cafeteria.
<img src="pictures/lunch.png" width="500" height="300" alt="lunch">
<<set $hypo to 2>>
[[Return to the ward]]<<nobr>>
<<if $hypo eq 1>> After having a fabled hour long lunch, you return to find that Brenda's blood sugar is 5mmol/L. You consult Dr Martin, who informs you that you should have decreased Brenda's insulin when her blood glucose dropped below 14mmol/L, prescribing dextrose simultaneously. He reinforces that the acidosis, and not the hyperglycaemia, is the main target of treatment in DKA and so insulin should not be stopped when blood glucose is normalising. <</if>> <<if $hypo eq 2>> After having a fabled hour long lunch, you return to find that Brenda's blood sugar is 7mmol/L. You consult Dr Martin, who informs you that you that you were correct to decrease Brenda's insulin when her blood glucose dropped below 14mmol/L, but you should have prescribing dextrose simultaneously in order to avoid hypoglycaemia. He reinforces that the acidosis, and not the hyperglycaemia, is the main target of treatment in DKA and so insulin should not be stopped when blood glucose is normalising. <</if>> <<if $hypo eq 3>> After having a fabled hour long lunch, you return to find that Brenda's blood sugar is 14mmol/L and Dr Martin has started the insulin you stopped. He informs you that you should have decreased (but not stopped) Brenda's insulin when her blood glucose dropped below 14mmol/L, prescribing dextrose simultaneously. He reinforces that the acidosis, and not the hyperglycaemia, is the main target of treatment in DKA and so insulin should not be stopped when blood glucose is normalising. <</if>> <<if $hypo eq 4>> After having a fabled hour long lunch, you return to find that Brenda's blood sugar is 7mmol/L. You consult Dr Martin, who informs you that you should have decreased Brenda's insulin when her blood glucose dropped below 14mmol/L, as well as prescribing the dextrose. He reinforces that the acidosis, and not the hyperglycaemia, is the main target of treatment in DKA and so insulin should be decreased but not be stopped when blood glucose is normalising. You decrease her insulin to 3 units per hour as per guidelines. <</if>> <<if $hypo eq 5>> After having a fabled hour long lunch, you return to find that Brenda's blood sugar is 10mmol/L. You consult Dr Martin, who congratulates you on managing Brenda appropriately. He reinforces that the acidosis, and not the hyperglycaemia, is the main target of treatment in DKA and so insulin should not be stopped when blood glucose is normalising. <<set $threehours += 1>> <</if>> <<if $hypo eq 6>> After having a fabled hour long lunch, you return to find that Brenda's blood sugar is 19mmol/L. You consult Dr Martin, who informs you that you should have decreased Brenda's insulin, not stopped it, when her blood glucose dropped below 14mmol/L (prescribing dextrose simultaneously to prevent hypoglycaemia). He reinforces that the acidosis, and not the hyperglycaemia, is the main target of treatment in DKA and so insulin should not be stopped when blood glucose is normalising. <</if>>
<</nobr>>
Brenda continues to make good progress until the [[end of your shift]].
After a busy morning you now feel like you've earned your lunch. You have a leisurely hour eating food that could only be rivalled by Wishaw's cafeteria.
<img src="pictures/lunch.png" width="500" height="300" alt="lunch">
<<set $hypo to 3>>
[[Return to the ward]]After a busy morning you now feel like you've earned your lunch. You have a leisurely hour eating food that could only be rivalled by Wishaw's cafeteria.
<img src="pictures/lunch.png" width="500" height="300" alt="lunch">
<<set $hypo to 4>>
[[Return to the ward]]After a busy morning you now feel like you've earned your lunch. You have a leisurely hour eating food that could only be rivalled by Wishaw's cafeteria.
<img src="pictures/lunch.png" width="500" height="300" alt="lunch">
<<set $hypo to 5>>
[[Return to the ward]]After a busy morning you now feel like you've earned your lunch. You have a leisurely hour eating food that could only be rivalled by Wishaw's cafeteria.
<img src="pictures/lunch.png" width="500" height="300" alt="lunch">
<<set $hypo to 6>>
[[Return to the ward]]You head home after a long day and catch up on some well earned [[sleep]]...
<<audio "backgroundhospital" stop>>Is that your alarm going off already? You get ready, curse the day you decided to study medicine, and head back to [[work]].
<<audio "alarm" volume 0.5 loop play>>You decide to stop in on Brenda before your shift to see how she's getting on...
Dr Dolittle, your friend from medical school, has taken over Brenda's care since she's been transferred. Having skipped the Diabetes Acute Care Day, he does not know when to switch Brenda from IV to subcutaneous insulin only. Her most recent BM was 7.8mmol/L. Should he and his team:
[[Wait until she is eating and her biochemistry has normalised. Ensure basal subcutaneous insulin is on board before stopping IV insulin->a]]
[[Wait until she is eating and her biochemistry has normalised. Stop IV insulin and then immediately give basal subcutaneous insulin->b]]
[[Wait until she is eating. As long as her glucose is <12mmol/L, her biochemistry does not need to be within reference range. Ensure basal subcutaneous insulin is on board before stopping IV insulin ->c]]
[[Wait until she is eating. As long as her glucose is <12mmol/L, her biochemistry does not need to be within reference range. Stop IV insulin and then immediately give basal subcutaneous insulin->d]]
<<nobr>>
<<audio "alarm" stop>>
<<audio "backgroundhospital" volume 0.05 loop play>>
<<if $FBC>> <<set $goodblood += 1>> <</if>>
<<if $UE>> <<set $goodblood += 1>> <</if>>
<<if $labglucose>> <<set $goodblood += 1>> <</if>>
<<if $Insulin>> <<set $badblood += 1>> <</if>>
<<if $CRP>> <<set $goodblood += 1>> <</if>>
<<if $VBG>> <<set $goodblood += 1>> <</if>>
<<if $ABG>> <<set $badblood += 1>> <</if>>
<<if $LFT>> <<set $goodblood += 1>> <</if>>
<</nobr>>
<<nobr>>
<<set $triad = ($polydipsia + $polyuria + $polyphagia + $nocturia + $weight)>> <<set $total = ($triad + $pointofcare + $goodblood - $badblood + $rightdiagnosis + $anionquestion + $firstbag + $thrombo + $initialinsulin + $threehours + $subcut + $kreplacement)>>
<</nobr>>
\_\_''Score breakdown''\_\_
\*Total score for important symptoms = <<print $triad>> out of 5
\*Total score for point of care tests = <<print $pointofcare>> out of 3
\*Total score for correct bloods = <<print $goodblood>> out of 6
\*Total score ''deducted'' for unnecessary bloods = <<print $badblood>> out of 2
\*Total score for diagnosis = <<print $rightdiagnosis>> out of 2
\*Total score for anion gap = <<print $anionquestion>> out of 1
\*Total score for initial fluids = <<print $firstbag>> out of 1
\*Total score for initial insulin infusion = <<print $initialinsulin>> out of 1
\*Total score for whether to replace potassium = <<print $kreplacement>> out of 1
\*Total score for thromboprophylaxis = <<print $thrombo>> out of 1
\*Total score for managing falling glucose = <<print $threehours>> out of 1
\*Total score for converting to S/C insulin = <<print $subcut>> out of 1
\*Overall score - <<print $total>> / 22
Continue to [[awards]]...
<<audio "backgroundhospital" stop>>
<<audio "sugar" volume 0.5 loop play>><<set $subcut = 1>> Correct Dr <<print$doctor>>! The consultant on the ward, Dr Hanssen, reinforces the fact that individuals with type 1 diabetes must always have long-acting basal insulin in their system. IV insulin has a very short half-life and should only be stopped once subcutaneous basal insulin has been given. Patients should also be eating and drinking, and have a normal biochemistry (ketones and bicarbonate within reference ranges).
Let's see how Brenda is now...
<video src="videos/muchbetter.mp4" width="640" height="480" controls></video>
Brenda will be seeing the <span class="greentext">diabetes team</span> soon, who will have a conversation with her about [[sick day rules]]...
Not quite Dr <<print$doctor>>! The consultant on the ward, Dr Hanssen, reinforces the fact that individuals with type 1 diabetes must always have long-acting basal insulin in their system. IV insulin has a very short half-life and should only be stopped once subcutaneous basal insulin has been given. Patients should also be eating and drinking, and have a normal biochemistry (ketones and bicarbonate within reference ranges).
Let's see how Brenda is now...
<video src="videos/muchbetter.mp4" width="640" height="480" controls></video>
Brenda will be seeing the <span class="greentext">diabetes team</span> soon, who will have a conversation with her about [[sick day rules]]...Not quite Dr <<print$doctor>>! The consultant on the ward, Dr Hanssen, reinforces the fact that individuals with type 1 diabetes must always have long-acting basal insulin in their system. IV insulin has a very short half-life and should only be stopped once subcutaneous basal insulin has been given. Patients should also be eating and drinking, and have a normal biochemistry (ketones and bicarbonate within reference ranges).
Let's see how Brenda is now...
<video src="videos/muchbetter.mp4" width="640" height="480" controls></video>
Brenda will be seeing the <span class="greentext">diabetes team</span> soon, who will have a conversation with her about [[sick day rules]]...Not quite Dr <<print$doctor>>! The consultant on the ward, Dr Hanssen, reinforces the fact that individuals with type 1 diabetes must always have long-acting basal insulin in their system. IV insulin has a very short half-life and should only be stopped once subcutaneous basal insulin has been given. Patients should also be eating and drinking, and have a normal biochemistry (ketones and bicarbonate within reference ranges).
Let's see how Brenda is now...
<video src="videos/muchbetter.mp4" width="640" height="480" controls></video>
Brenda will be seeing the <span class="greentext">diabetes team</span> soon, who will have a conversation with her about [[sick day rules]]...H+ - 57 nmol/L (35–45)
Bicarbonate - 11 mmol/L (21–29)
O2 - 7.8kPa (normal for venous blood gas)
CO2 - 2.1kPa (low for venous blood gas)
Na+ - 146 mmol/L (133–144)
K+ - 5.1 mmol/L (3.5-5.2)
Cl- - 101 mmol/L (95–107)
Lactate 2.4 mmol/L (0.6–1.8)
Glucose - 26 mmol/L (4.0–6.0)
Dr <<print$doctor>>, have you arrived at a [[diagnosis]]?<<if $triad gt 2>> <img src="pictures/trophy.png" width="60" height="80" alt="trophy"> Congratulations Dr <<print $doctor>>, you've earned the ''Art of Medicine Award'' for a thorough history. History makes up about 90% of the diagnosis doesn't it? We'll know who to call when our capillary glucose machine is broken!<</if>> <<if $triad lt 3>> <img src="pictures/notrophy.png" width="60" height="80" alt="trophy"> Try playing through again to unlock this award. <</if>>
<<if $thrombo eq 1>> <img src="pictures/trophy.png" width="60" height="80" alt="trophy"> Conratulations Dr <<print $doctor>>, you've earned the ''Triangle Award''! What is the triangle award you ask? Well it's really a triad - Virchow's to be precise. You never forget DVT prophylaxis, do you?! I look forward to seeing your multiple rounds of DVT prophylaxis audit work. <</if>> <<if $thrombo eq 0>> <img src="pictures/notrophy.png" width="60" height="80" alt="trophy"> Try playing through again to unlock this award. <</if>>
<<if $goodblood gt 3 and $badblood lt 1>> <img src="pictures/trophy.png" width="60" height="80" alt="trophy"> Congratulations Dr <<print $doctor>>, you've earned the ''Saviour of the NHS Award''. Why? You saved NHS some precious £££ by not ordering lots of unnecessary tests! <<else>> <img src="pictures/notrophy.png" width="60" height="80" alt="trophy"> Try playing through again to unlock this award. <</if>>
Thanks for playing the game!
[[Restart]]
Disclaimer: This was a work of fiction, first created in 2018. Names are products of the author's imagination. Any resemblance to actual persons is entirely coincidental-ish.
Illness can be categorised as minor and severe:
\*<span class="greentext">Minor</span> - Normal or raised glucose, no ketones or trace ketones on urinalysis, capillary ketones of <1.5mmol/L. This is usually due to a minor viral illness or a minor injury.
\*<span class="redtext">Severe</span> - Raised glucose, ketones on urinalysis, capillary ketones of >1.5mmol/L. Pneumonia, for example, might lead to these conditions and be classified as a severe illness.
Dr Hanssen explains to Brenda that hormones, such as cortisol, are released during periods of illness. These raise the blood glucose level and increase insulin requirements. They could therefore lead to an episode of <span class="redtext">diabetic ketoacidosis</span>. It is important for Brenda to realise that, even if she is eating and drinking less than she usually would, she may still require more insulin than normal. Regular checking of blood glucose and ketones is essential - this should be performed every 4 hours, or every 2 hours if ketones are present. It is important that Brenda ensures that she has an adequate supply of in-date equipment in her house for periods of illness.
When she is ill, Brenda should ensure that she drinks 100ml-200ml of sugar-free liquid per hour to prevent her from becoming dehydrated. If she is unable to eat, she should also drink some carbohydrate-containing fluids such as fruit juice or milk (as required).
Insulin dose adjustment can be difficult - especially during illness. Diabetes Specialist Nurses may be able to help Brenda with this. Brenda may also take part in a <span class="greentext">DAFNE (Dose Adjustment For Normal Eating)</span> course in the future. This helps individuals to learn how to live as normal a life as possible with diabetes and enables them to become experts in their condition.
Now that Brenda's case has been managed appropriately, you are given some feedback on [[your performance]].Welcome to the <span class="greentext"> Acute Care Virtual Patient Simulation - Scenario 2</span>!
This was developed by Dr Nat Quail, under the close supervision of Dr James Boyle\*. We would like to acknowledge the generous grant received from the Learning and Teaching Development Fund, which enabled us to pay for actors and equipment.
We hope that this game will enable you to learn more about the management of specific conditions and acutely ill patients in a fun\*\* and safe manner.
[[Instructions]]
So it's his fault if something doesn't work right\*
May not be fun\*\*Just a few quick notes before you start:
\*If the question is multi-choice, make sure you finalise your answers before ticking the box. Some multi-choice questions require more than one answer.
\*For most questions you will be scored on how you answer. If you pick more choices than are necessary (for example, requesting unnecessary blood tests) then you may lose points.
\*Click on the coloured links within passages to continue
\*''Watch out for your web-browser trying to autocorrect free-text answers''
[[Introduction]] <<script>>
state.restart();
<<endscript>>
