# Supplementary material for: Twine virtual patient games as an online resource for undergraduate diabetes acute care education
Source: BMC Med Educ. 2023 Jun 7;23:417. doi: 10.1186/s12909-023-04231-2 (PMC10244842; doi:10.1186/s12909-023-04231-2)
Supplement: Supplementary file 3 — Supplementary Material 3: Virtual Patient Game 3 [file 12909_2023_4231_MOESM3_ESM.html]

Virtual Patient Three


JavaScript must be enabled to play.

Browser lacks capabilities required to play.

Upgrade or switch to another browser.

Loading…

 Dr <<print$doctor>>, this is Mr Dukes, an elderly gentleman who has been brought in from home by ambulance after his daughter found him in a confused and breathless state. The paramedics have established that he has type 2 diabetes - the capillary glucose reading simply reads "HI". Nurse Boyle starts setting up for observations while you chat to Mr Dukes. You begin by asking his name and date of birth:
<video src="videos/namedob3.mp4" width="640" height="480" controls></video>
His date of birth is correct. You want to further assess how orientated he is - what other questions would you ask to complete a simple AMT4 assessment ''(choose 3)''?
What year is this? <<checkbox "$year" false true unchecked>>
What is the name of current monarch? <<checkbox "$monarch" false true unchecked>>
What time is it (to the nearest hour)? <<checkbox "$time" false true unchecked>>
Where are you? <<checkbox "$place" false true unchecked>>
When did World War I begin? <<checkbox "$wwi" false true unchecked>>
What age are you? <<checkbox "$age" false true unchecked>>
[[Let's see]]
<<set $history1 =0>>
<<set $history2 =0>>
<<set $history3 =0>>
<<set $history4 =0>>
<<if $history1 eq "0" and $history2 eq "0" and $history3 eq "0" and $history4 eq "0">> <<if $year>> <video src="videos/2007.mp4" width="640" height="480" controls> </video>
Well done, you asked him what year it is.<<set $amt4 += 1>> <</if>>
<<if $age>><video src="videos/age.mp4" width="640" height="480" controls></video> <<set $amt4 += 1>>
Well done, you asked him his age. <</if>>
<<if $place>> <video src="videos/whereareyou.mp4" width="640" height="480" controls></video>
Well done, you asked him where he is at the moment. <<set $amt4 += 1>> <</if>>
The AMT4 is a quick screening tool for cognitive impairment, which consists of asking the patient their date of birth, their age, what year it is, and where they are. It forms part of the 4AT assessment for delirium and this would be carried out in Mr Dukes' case, due to his age and presentation. <</if>>
Do you want to ask Mr Dukes:
<<actions
[[About medications]]
[[PMH]]
[[Any pain?]]
[[When did the breathlessness begin?]]>>
<video src="videos/medication.mp4" width="640" height="480" controls></video>
<<set $history1 =1>>
<<if $history1 eq 1 and $history2 eq 1 and $history3 eq 1 and $history4 eq 1>> [[Continue History->Cut to the chase]] <</if>>
<<if $history1 neq 1 or $history2 neq 1 or $history3 neq 1 or $history4 neq 1>> [[Continue History->Let's see]] <</if>><video src="videos/pastmedicalhistory.mp4" width="640" height="480" controls></video>
<<set $history2 =1>>
<<if $history1 eq 1 and $history2 eq 1 and $history3 eq 1 and $history4 eq 1>> [[Continue History->Cut to the chase]] <</if>>
<<if $history1 neq 1 or $history2 neq 1 or $history3 neq 1 or $history4 neq 1>> [[Continue History->Let's see]] <</if>><video src="videos/needwater.mp4" width="640" height="480" controls></video>
<<set $history3 =1>>
<<if $history1 eq 1 and $history2 eq 1 and $history3 eq 1 and $history4 eq 1>> [[Continue History->Cut to the chase]] <</if>>
<<if $history1 neq 1 or $history2 neq 1 or $history3 neq 1 or $history4 neq 1>> [[Continue History->Let's see]] <</if>>It is difficult to tell whether there is an abnormality on percussion, so turn up your volume and have a listen!
His oxygen saturations are 93% - he is on a 15L trauma mask but hasn't been keeping his mask on until now. His respiratory rate is 18 breaths per minute.
This is what he has coughed up:
<img src="pictures/mucus.jpg" width="400" height="400" alt="mucus">
Here was your first list of differentials for his breathlessness:
$differentials1
$differentials2
$differentials3
Have they changed? Enter your new differentials below.
<<textbox "$differentials4" "">>
<<textbox "$differentials5" "">>
<<textbox "$differentials6" "">>
You ask Nurse Boyle to send a sputum sample for culture and sensitivity.
[[Progress]]...
<<audio "backgroundhospital" stop>>
<<audio "breathsounds" volume 2 loop play>>What \_\_first line\_\_ tests might you want to perform/organise to further assess his \_\_respiratory system\_\_ specifically? (1 blood test, 1 imaging test)
<<textbox "$test1" "blood test">>
<<textbox "$test2" "imaging test">>
You [[continue your A-E assessment...->C]]
<<audio "backgroundhospital" volume 0.05 loop play>>
<<audio "breathsounds" stop>>Mr Dukes' blood pressure is 93/63 mmHg, his capillary refill peripherally is 4 seconds, and his pulse is regular at 96 BPM. His mucous membranes are dry and he has not passed any urine today. Turn the volume up and have a listen to his heart!
You arrange a 12 lead ECG and catheterisation to monitor urine output.
You also mannage to cannulate him. What bloods do you want and why? Remember to tick the box and give a reason - try not to order any unnecessary tests!
<<checkbox "$FBC" false true unchecked>> FBC <<textbox "$FBCreason" "reason?">>
<<checkbox "$UE" false true unchecked>> U&Es <<textbox "$UEreason" "reason?">>
<<checkbox "$LFT" false true unchecked>> LFTs <<textbox "$LFTreason" "reason?">>
<<checkbox "$Bprofile" false true unchecked>> Bone profile <<textbox "$Bprofilereason" "reason?">>
<<checkbox "$labglucose" false true unchecked>> Formal glucose <<textbox "$labglucosereason" "reason?">>
<<checkbox "$DDimer" false true unchecked>> D-Dimer <<textbox "$DDimerreason" "reason?">>
<<checkbox "$Insulin" false true unchecked>> Insulin <<textbox "$Insulinreason" "reason?">>
<<checkbox "$magnesium" false true unchecked>> Magnesium <<textbox "$magnesiumreason" "reason?">>
<<checkbox "$bloodcultures" false true unchecked>> Blood cultures <<textbox "$bloodculturesreason" "reason?">>
<<checkbox "$CRP" false true unchecked>> CRP <<textbox "$CRPreason" "reason?">>
<<checkbox "$HBA1C" false true unchecked>> HbA1c <<textbox "$HBA1Creason" "reason?">>
[[Send off samples]].
<<audio "backgroundhospital" stop>>
<<audio "heartsounds" volume 3 loop play>>
<<audio "heartsounds" stop>> <<audio "backgroundhospital" volume 0.05 loop play>> Your senior, Dr Jandhu, tells you exactly what bloods she wants and why:
<span class="greentext"> \_\_Bloods she wants\_\_ </span>
<<timed 1s>> "I want a full blood count to check the haemoglobin and white cell count."
<<if $FBC>>Your reason was: <span class="greentext"> \_\_ $FBCreason \_\_</span><</if>>
<<next>> “CRP - ?infection."
<<if $CRP>>Your reason was: <span class="greentext"> \_\_ $CRPreason \_\_</span><</if>>
<<next>> "U&Es - for electrolytes and renal function."
<<if $UE>>Your reason was: <span class="greentext"> \_\_ $UEreason \_\_</span><</if>>
<<next>> "LFTs may have been useful as a baseline in this acute setting if we plan on starting any treatment (for example, antibiotics)."
<<if $LFT>>Your reason was: <span class="greentext"> \_\_ $LFTreason \_\_</span><</if>>
<<next>> "Glucose - point of care glucose only detects up to a certain value."
<<if $labglucose>>Your reason was: <span class="greentext"> \_\_ $labglucosereason \_\_</span><</if>>
<<next>> "Although the patient is not pyrexial, blood cultures will be useful as he is clearly unwell and there looks to be an infection contributing to this."
<<if $bloodcultures>>Your reason was: <span class="greentext"> \_\_ $bloodculturesreason \_\_</span><</if>>
<<next>> "An HbA1c might be useful to send to tell us about long-term diabetes control."
<<if $HBA1C>>Your reason was: <span class="greentext"> \_\_ $HBA1Creason \_\_</span><</if>>
<<next>> "It would be useful to check a magnesium level, given the profound dehydration."
<<if $magnesium>>Your reason was: <span class="greentext"> \_\_ $magnesiumreason \_\_</span><</if>>
<<next>> "It would be useful to check a bone profile, given the profound dehydration."
<<if $Bprofile>>Your reason was: <span class="greentext"> \_\_ $Bprofilereason \_\_</span><</if>>
<span class="redtext"> \_\_Bloods she does not want\_\_ </span>
<<next>> "Insulin levels are not routinely checked in hospitals - we might check C-peptide to differentiate between type 1 and 2 diabetes but it would certianly not be an admission investigation. The patient does not have chest pain, syncope, or haemoptysis and has likely diagnoses that do not include a PE and so a D-dimer should not be ordered at this stage due to its low specificity."
<<next>> [[Continue->C2]] <</timed>>
<<if $FBC>> <<set $goodbloodanswers += 1>> <</if>>
<<if $magnesium>> <<set $goodbloodanswers += 1>> <</if>>
<<if $Bprofile>> <<set $goodbloodanswers += 1>> <</if>>
<<if $HBA1C>> <<set $goodbloodanswers += 1>> <</if>>
<<if $UE>> <<set $goodbloodanswers += 1>> <</if>>
<<if $CRP>> <<set $goodbloodanswers += 1>> <</if>>
<<if $labglucose>> <<set $goodbloodanswers += 1>> <</if>>
<<if $bloodcultures>> <<set $goodbloodanswers += 1>> <</if>>
<<if $LFT>> <<set $goodbloodanswers += 1>> <</if>>
<<if $DDimer>> <<set $badbloodanswers += 1>> <</if>>
<<if $Insulin>> <<set $badbloodanswers += 1>> <</if>><<set $abg2 = 0>>Mr Dukes' temperature is 37.2'C, his glucose still reads as 'HI', his GCS is 14 (confused) and his pupils are equal and reactive to light. You ensure a culture of his sputum has been sent away.
Under D, you also want to assess the patient's medication for any reversible causes of their presentation. Mr Dukes' repeat prescription was in his jacket:
<img src="pictures/prescription.jpg" width="400" height="600" alt="prescription">
[[Continue...->E]]
<<set $test2 to $test2.toLowerCase()>> <<set $test2 to $test2.trim()>> <<if $test2 .includes ("xray") or $test2 .includes ("x-ray") or $test2 .includes ("cxr") or $test2 .includes ("c-xr")>> <<else>> The radiographers are here to perform a chest x ray...did you request this? <<set $CXRtotal -=1>><</if>>
<<set $chestxray = 0>> <<set $ecg = 0>> <<set $test1 to $test1.toLowerCase()>> <<set $test1 to $test1.trim()>> <<if $test1 .includes ("abg") or $test1 .includes ("arterial") or $abg2 eq 1>>\_\_Selected ABG results\_\_
''Taken shortly after the patient was put on 15L of oxygen, although he keeps removing the mask to talk''
\*H+ - 32 nmol/L (35–45)
\*PaO2 - 16 kPa (11.3–12.6)
\*PaCO2 - 2.8 kPa (4.7–6.0)
\*HCO3 - 22 mmol/L (21–29)
\*Lactate - 1.2 mmol/L (0.5–1.6)
\*Glucose - >33 mmol/L (4-6)
\*Na+ - 139 nmol/L (133-144)
\*K+ - 4.8 mmol/L (3.5-5.1)
Nurse Boyle has obtained a urine dipstick. This is negative for haemoglobin, ketones, leukocytes, and nitrites. It is 4+ for glucose.
<<if $respiratory neq "wrong" and $respiratory neq "correct">>How would you describe the acid-base balance of this blood gas?
Primary respiratory alkalosis <<radiobutton "$respiratory" "correct">>
Primary metabolic alkalosis <<radiobutton "$respiratory" "wrong">>
Primary respiratory acidosis <<radiobutton "$respiratory" "wrong">>
Primary metabolic acidosis <<radiobutton "$respiratory" "wrong">>
[[Check->Available]] <</if>>
<<if $respiratory eq "wrong" or $respiratory eq "correct">> <<if $respiratory eq "wrong">> Incorrect. The H+ result shows an alkalosis (a decrease in H+ ions and therefore and increase in pH). The PaCO2 is reduced due to an increased respiratory rate and so this is a primary respiratory alkalosis. The bicarbonate is normal, showing there is not yet any metabolic compensation. Since Mr Dukes' lack of insulin is relative rather than absolute - as in DKA - he will likely produce little or no ketones and so this will not have an effect on the acid-base balance. Hypoxaemia was likely the cause of the Mr Dukes' increased respiratory rate. The O2 is still low considering he is on 15L of oxygen, although he does keep taking his mask off. <</if>> <<if $respiratory eq "correct">> Correct. The H+ result shows an alkalosis (a decrease in H+ ions and therefore and increase in pH). The PaCO2 is reduced due to an increased respiratory rate and so this is a primary respiratory alkalosis. The bicarbonate is normal, showing that metabolic compensation has yet to take place. Since Mr Dukes' lack of insulin is relative rather than absolute - as in DKA - he will likely produce little or no ketones and so this will not have an effect on the acid-base balance. Hypoxaemia was likely the cause of the Mr Dukes' increased respiratory rate. The O2 is still low considering he is on 15L of oxygen, although he does keep taking his mask off. <<set $ABGinterpretation += 1>><</if>>
Mr Dukes sats are now 96% - you aim to keep him in the range of 94-98% since there is no evidence of CO2 retention. You will titrate down the oxygen therapy as able.
[[View Mr Dukes' ECG]]
[[View Mr Dukes' Chest X Ray]] <</if>> <<else>>
Dr Jandhu demands to know why you have not yet sent away an arterial blood gas - you quickly obtain one and ask Nurse Boyle to run the sample. <<set $ABGtotal -=1>> <<set $abg2 = 1>>[[Wait for results->Available]] <</if>><img src="pictures/ecg.jpg" width="600" height="400" alt="ecg">
Which of the \_\_three\_\_ findings below are present on this ECG?
Poor R wave progression <<checkbox "$heart1" false true unchecked>>
Left axis deviation <<checkbox "$heart2" false true unchecked>>
T wave inversion in the inferior leads <<checkbox "$heart3" false true unchecked>>
Ventricular premature beat <<checkbox "$heart4" false true unchecked>>
Left bundle branch block <<checkbox "$heart5" false true unchecked>>
What does this ECG, in combination with Mr Dukes' presentation and medication, most likely suggest about his cardiovascular history?
Previous MI <<radiobutton "$previousmi" "correct">>
Paroxysmal AF <<radiobutton "$previousmi" "wrong">>
Aortic Stenosis <<radiobutton "$previousmi" "wrong">>
Acute coronorary event <<radiobutton "$previousmi" "wrong">>
[[Consult with Dr Jandhu]]<img src="pictures/xray.jpg" width="400" height="400" alt="xray">
Here was your latest list of differential causes for Mr Dukes' breathlessness -
$differentials4
$differentials5
$differentials6
What is the diagnosis?
<<textbox "$bdiagnosis" "">>
[[Let's check]]<img src="pictures/ecg.jpg" width="600" height="400" alt="ecg">
\_\_Abnormalities\_\_
\*<<if $heart1>> <<set $ECGtotal +=1>> Correct! The normal ECG has a predominant S wave in V1 (negative deflection), with the R wave progressing to become more dominant at around V3/V4 (positive deflection). If the R wave does not progress as normal then this can indicate an old anterior MI.<<else>>You missed poor R wave progression. The normal ECG has a predominant S wave in V1 (negative deflection), with the R wave progressing to become more dominant at V3/V4 (positive deflection). If the R wave does not progress as quickly then this can indicate an old anterior MI.<</if>>
<img src="pictures/rprogression.jpg" width="600" height="200" alt="rprogression">
\*<<if $heart2>> You incorrectly chose left axis deviation - there is no axis deviation in this ECG (the QRS is predominantly positive in leads I and II).<<else>> <<set $ECGtotal +=1>> Correct! There is no axis deviation in this ECG (the QRS is predominantly positive in leads I and II). <</if>>
<img src="pictures/axis.jpg" width="600" height="200" alt="axis">
\*<<if $heart3>> <<set $ECGtotal +=1>> Well done! There is indeed T wave inversion in the inferior leads (II, III, aVF). These changes do not necessarily represent an acute occlusive coronorary event, especially in the context of hypoxia and with the patient lacking any chest pain. It would be useful, however, to check that this is present on previous ECGs as diabetes can mask the signs and symptoms of myocardial infarction. <<else>> You missed that there was indeed T wave inversion in the inferior leads (II, III, aVF). These changes do not necessarily represent an acute occlusive coronorary event, especially in the context of hypoxia and with the patient lacking any chest pain. It would, however, be useful to ensure that this was present on previous ECGs as diabetes can mask the signs and symptoms of myocardial infarction. <</if>>
\*<<if $heart4>> <<set $ECGtotal +=1>> Correct! A ventricular premature beat is present. This is an abnormal beat, which has a wide QRS, and is not associated with a P wave. It can be a variant of normal, or can be caused by ischaemia or left ventricular dysfunction. <<else>>You missed the ventricular premature beat. This is an abnormal beat, which has a wide QRS, and is not associated with a P wave. It can be a variant of normal, or can be caused by ischaemia or left ventricular dysfunction. <</if>>
\*<<if $heart5>> There is no left bundle branch block - the QRS is narrow, not broad (apart from the ventricular premature beat).<<else>> <<set $ECGtotal +=1>> Well done for not choosing left bundle branch block - the QRS is narrow, not broad (apart from the ventricular premature beat). <</if>>
\_\_What do these suggest\_\_
\*<<if $previousmi eq "correct">> <<set $previousmianswer +=1>> Well done! Mr Dukes is on medication that would be started post-MI (dual antiplatelet therapy, an ace-inhibitor, and a beta-blocker). He has presented without chest pain and so the poor R wave progression may indicate old ischaemia. There may be an element of Type 2 MI given the initial hypoxia. To be safe, you would check these were longstanding changes against the patient's medical history and old ECGs. <</if>> <<if $previousmi neq "correct">> Mr Dukes is on medication that would be started post-MI (dual antiplatelet therapy, an ace-inhibitor, and a beta-blocker). He has presented without chest pain and so the poor R wave progression may indicate old ischaemia. There may be an element of Type 2 MI given the initial hypoxia. To be safe, you would check these were longstanding changes against the patients online medical history or old ECGs. <</if>>
<<set $ecg =1>>
<<if $chestxray eq 0>> [[View Mr Dukes' Chest X Ray]] <</if>>
<<if $chestxray eq 1>> [[Continue]] <</if>>
<<if $ECGtotal eq "5" and $previousmi eq "correct" >> <<set $jpl = 1>> <</if>><<set $bdiagnosis to $bdiagnosis.toLowerCase()>> <<set $bdiagnosis to $bdiagnosis.trim()>> <<if $bdiagnosis .includes ("pneumonia") or $bdiagnosis .includes ("cap")>> Correct! <<set $pneumoniaanswer += 1>><<else>> The correct diagnosis is pneumonia. <</if>>
<<set $chestxray =1>>
<<if $ecg eq 0>> [[View Mr Dukes' ECG]] <</if>>
<<if $ecg eq 1>> [[Continue]] <</if>><video src="videos/cuttothechase.mp4" width="640" height="480" controls></video>
You try to ask Mr Dukes more about his presentation, but you can't get any more history. Mr Dukes' airway is obviously patent with no worrying noises and so you begin to assess his [[B]]...Dr Jandhu would like you to prescribe some oral empirical antibiotics for Mr Dukes' pneumonia and so you consult the local protocol. It is assumed that this is a typical pneumonia. It first asks you to calculate his CURB-65, a marker of severity and prognosis. Although this might be artificially worsened by his concurrent hyperglycaemic illness, Dr Jandhu advises you to use this score as it is best to be on the safe side.
Mr Dukes' respiratory rate was initially 24 breaths per minute with the ambulance crew. His heart rate is 97 BPM and his blood pressure is 96/78mmHg. Dr Jandhu advises you to assume the urea will be higher than 7mmol/L, due to Mr Dukes' profound dehydration on top of the pneumonia.
What is Mr Dukes' CURB-65 score?
0 <<radiobutton "$curb" "wrong">>
1 <<radiobutton "$curb" "wrong">>
2 <<radiobutton "$curb" "wrong">>
3 <<radiobutton "$curb" "correct">>
4 <<radiobutton "$curb" "wrong">>
5 <<radiobutton "$curb" "wrong">>
[[Continue->curb65]]Based on the fact that Mr Dukes appears severely dehydrated and has a capillary glucose reading of 'HI', what is the likely diagnosis causing this part of his clinical presentation?
<<textbox "$diagnosis" "">>
Is this likely to account for Mr Dukes' breathing problems?
Yes <<radiobutton "$hhs" "wrong">>
No <<radiobutton "$hhs" "correct">>
What fluid do you prescribe?
1L NaCl (0.9%) over 4 hours <<radiobutton "$fluid" "wrong">>
1L NaCl (0.9%) over 1 hour <<radiobutton "$fluid" "correct">>
1L NaCl (0.45%) over 4 hours <<radiobutton "$fluid" "wrong">>
1L NaCL (0.45%) over 1 hour <<radiobutton "$fluid" "wrong">>
[[Continue...->Chalktalk]]<<set $diagnosis to $diagnosis.toLowerCase()>> <<set $diagnosis to $diagnosis.trim()>> <<if $diagnosis .includes ("hhs") or $diagnosis .includes ("hyperosmol") or $diagnosis .includes ("honc") or $diagnosis .includes ("hyper-osm")>> Well done - the diagnosis is likely to be one of hyperosmolar hyperglycaemic state (formerly hyperosmolar nonketotic coma).<<set $diagnosisanswer +=1>><<else>> The diagnosis is likely to be one of hyperosmolar hyperglycaemic state (formerly hyperosmolar nonketotic coma). <</if>> <<if $hhs eq "correct">> <<set $otherpresentation += 1>><</if>>
This normally does not produce ketosis and so you would not expect Mr Dukes to be breathless from trying to compensate for a metabolic acidosis, as happens in DKA. Here is a short video detailing key differences between HHS and DKA:
<video src="videos/dkatwinehhs.mp4" width="640" height="480" controls></video>
<<if $fluid eq "correct">> You prescribed the correct fluid, well done Dr $doctor!<<set $initialfluids += 1>><</if>> <<if $fluid eq "wrong">> Dr Jandhu informs you that you prescribe 1L NaCl (0.9%) over 1 hour.<</if>> The main treatment in HHS is fluid replacement. This is started fairly quickly, with 1l of NaCl (0.9%) given over 1 hour, and then the rate is reduced with subsequent fluid replacement.
\*Give the 1st litre over 1 hour
\*2nd litre over 2 hours
\*3rd litre over 4 hours
\*4th litre over 6 hours
\*5th litre over 8 hours
In the abscence of significant ketonaemia, current Greater Glasgow & Clyde guidlines (2021) suggest against giving insulin until fluid resuscitation alone fails to bring levels down. Other local protocols may suggest starting a low dose insulin infusion, but it is important that hyperglycaemia is \_\_not\_\_ aggressively treated with insulin to prevent vascular collapse.
Dr Jandhu decides not to give any insulin at this point. You [[continue your assessment...->D]]<<if $prescribe eq "wrong">> You go to write the medication in the kardex and realise that you've not checked for drug allergies or potential interactions.<</if>>You bring up Mr Dukes' emergency care summary and see that he is <span class="redtext"> penicillin allergic - causing anaphylaxis </span>. You also check drug interactions using the BNF and see that clarithromycin is contraindicated with statin and ticagrelor use because of its inhibition of cytochrome P450. In complex cases like these it would always be useful and safe to get a second opinion from your senior and you do this before filling out the kardex. You are advised to prescribe levofloxacin and so you do this.
You also document Mr Dukes' penicillin allergy on his kardex and in his notes. <<if $prescribe eq "correct">> <<set $checkallergy += 1>>''Dr Jandhu congratulates you on your safe prescribing''.<<set $walters = 1>><</if>>
[[Some of Mr Dukes' bloods are back->Ask the formula first]]
\_\_Selected results\_\_ <<if $osmole eq 0>> <<set $potassium to random(2)>> <<set $sodium to random(2)>> <</if>> <<set $osmole += 1>>
\*K+ <<if $potassium eq 0>>3.8 mmol/L<</if>><<if $potassium eq 1>>4.7 mmol/L<</if>><<if $potassium eq 2>>5.2 mmol/L<</if>> (3.5-4.9)
\*Na+ - <<if $sodium eq 0>>150 mmol/L<</if>><<if $sodium eq 1>>152 mmol/L<</if>><<if $sodium eq 2>>154 mmol/L<</if>> (137-144)
\*Cl- 101 mmol/L (95–107)
\*eGFR - 43 ml/min/1.73m^2 (result 6 months previously - 57 ml/min/1.73m^2)
\*Urea - 14 mmol/L (2.5-7.0)
\*Creatinine - 151 umol/L (60–110)
\*Glucose - 38 mmol/L (3-6)
\*LFTs - Within reference range
Dr Jandhu asks you to calculate Mr Dukes' osmolality (potassium is sometimes included in this calculation - on this occasion please do not include it).
<<if $osmole >1>> ''(2xSodium + Urea + Glucose)'' <</if>>
<<textbox "$osmolality" "enter number only">> mOsmol/kg
[[Does Dr Jandhu agree]]? <<if $osmole eq 1>> or [[Ask the formula first]] <</if>> <<if $osmole eq 2>> <<set $osmolalitytotal -= 1>> <</if>>
You have checked Mr Dukes' repeat prescription against his emergency care summary and all medications seem current and correct.
<img src="pictures/prescription.jpg" width="400" height="600" alt="prescription">
Which medications would you like to \_\_withhold\_\_ during this acute phase?
You use the BNF to help inform you decisions, taking into account why the patient is on each medication, and whether the dose they are on is acceptable/ potentially harmful: https://bnf.nice.org.uk/
Withhold aspirin <<checkbox "$aspirin" false true unchecked>>
Withhold atenolol <<checkbox "$atenolol" false true unchecked>>
Withhold atorvastatin <<checkbox "$atorvastatin" false true unchecked>>
Withhold gabapentin <<checkbox "$gabapentin" false true unchecked>>
Withhold Humulin M3 <<checkbox "$humulin" false true unchecked>>
Withhold metformin <<checkbox "$metformin" false true unchecked>>
Withhold pantoprazole <<checkbox "$pantoprazole" false true unchecked>>
Withhold ramipril <<checkbox "$ramipril" false true unchecked>>
Withhold ticagrelor <<checkbox "$ticagrelor" false true unchecked>>
Let's see [[if Dr Jandhu agrees]]...<span class="greentext"> \_\_Drugs to prescribe\_\_ </span>
\*Ticagrelor - On balance it would be better to continue antiplatelet medication to prevent a vascular event and there are no major contraindications. It would, however, not be completely wrong to withhold it on admission given the fact that the patient will likely struggle to take tablets with this acute illness.
<span class="yellowtext">\_\_Drugs you may want to prescribe, withhold, or reduce the dose of\_\_ </span>
\*Aspirin - This small dose of aspirin will likely not potentiate Mr Dukes' AKI, and there would be benefit in him continuing it acutely to reduce risk of a cardiovascular event. However, it would not be unreasonable to withhold it acutely due to concerns around renal function.
\*Atorvastatin - It will be of little benefit to Mr Dukes' long-term cardiovascular risk to continue his statin during his acute admission. There is some data that high potency statins may increase the risk of AKI in hospitalised patients, though this is not certain. It may also interact with antibiotics prescribed during admission. However, there is an argument to leave it on the drug prescription so that it is not missed on discharge and only suspend it if interacting medications are commenced.
\*Gabapentin - Gabapentin accumulates during an AKI and so the dose should be monitored if continuing.
\*Pantoprazole - We do not know if Mr Dukes is at high risk of a GI bleed, but he is on dual antiplatelet therapy, so it is likely acceptable to continue this. Some evidence does, however, exist that PPIs can cause electrolyte disturbance so in the setting of HHS it may also be acceptable to withhold this drug.
<span class="redtext">\_\_Drugs to withhold\_\_ </span>
\*Atenolol - Mr Dukes is fluid deplete and hypotensive and so this should be withheld.
\*Ramipril - Mr Dukes is fluid deplete, hypotensive, and has an AKI. Therefore this should be withheld.
\*Humulin M3 - Giving this insulin, which contains a short-acting component, could lead to cardiovascular collapse in the acute setting, and would be better being introduced in a controlled fashion via an IV infusion pump.
\*Metformin - Oral antidiabetic drugs are usually withheld in HHS until the patient has recovered. If metformin is continued with an AKI, it may precipitate lactic acidosis.
[[You write up the medications]]
<<if $atenolol>> <<set $medrec +=1>> <</if>>
<<if $ramipril>> <<set $medrec +=1>> <</if>>
<<if $humulin>> <<set $medrec +=1>> <</if>>
<<if $metformin>> <<set $medrec +=1>> <</if>><<if $sodium eq "0" and $osmolality eq "352">> Correct! <</if>> <<if $sodium eq "1" and $osmolality eq "356">> Correct! <</if>> <<if $sodium eq "2" and $osmolality eq "360">> Correct! <</if>> <<if $sodium eq "0" and $osmolality neq "352">> Not quite, the correct answer was 352 mOsmol/kg. <<set $osmolalitytotal -= 1>> <</if>> <<if $sodium eq "1" and $osmolality neq "356">> Not quite, the correct answer was 356 mOsmol/kg. <<set $osmolalitytotal -= 1>> <</if>> <<if $sodium eq "2" and $osmolality neq "360">> Not quite, the correct answer was 360 mOsmol/kg. <<set $osmolalitytotal -= 1>> <</if>>
Regularly calculating the osmolality, or formally measuring it with a blood test, is important in determining how concentrated the solutes are in Mr Dukes blood and therefore whether treatment is effective.
Although Mr Dukes' potassium is currently within the normal range, his total store is depleted and so this value will decrease with fluid replacement. Will you need to add potassium to the next set of fluids (bearing in mind his measured potassium was <<if $potassium eq 0>>3.8 mmol/L<</if>><<if $potassium eq 1>>4.7 mmol/L<</if>><<if $potassium eq 2>>5.2 mmol/L<</if>>)?
Yes <<radiobutton "$kfluid" "yes">>
No <<radiobutton "$kfluid" "no">>
What other interventions are important in the management of Mr Dukes over the next hours to days?
Thromboprophylaxis <<checkbox "$thrombo" false true unchecked>>
Heel protection and daily foot checks <<checkbox "$foot" false true unchecked>>
Urgent change to regular insulin regimen <<checkbox "$regimen" false true unchecked>>
Refer patient to HDU for assessment <<checkbox "$HDU" false true unchecked>>
[[Let's ask Dr Jandhu]]
\_\_Potassium\_\_
<img src="pictures/potassium.jpg" width="400" height="400" alt="potassium">
<<if $potassium eq 0 and $kfluid eq "yes">> <<set $kanswer += 1>> Correct! <</if>> <<if $potassium eq 1 and $kfluid eq "yes">> <<set $kanswer += 1>> Correct! <</if>> <<if $potassium eq 2 and $kfluid eq "no">> <<set $kanswer += 1>> Correct! <</if>> <<if $potassium eq 0 and $kfluid neq "yes">> Not quite! <</if>> <<if $potassium eq 1 and $kfluid neq "yes">> Not quite! <</if>> <<if $potassium eq 2 and $kfluid neq "no">> Not quite! <</if>>
\*If potassium is being given faster than 10mmol/hour then ecg monitoring is recommended. Interestingly, sodium ''measurement'' can rise with fluid replacement and needs to be monitored.
\_\_Other management\_\_
\*Thromboprophylaxis is necessary in a patient presenting with HHS. This is due to the patient being profoundly dehydrated and having stasis of blood, as well as the pre-existing endothelial wall dysfunction caused by their diabetes.
\*Patients presenting with HHS are also at high risk of developing diabetic foot complications and so this must be closely monitored.
\*Although a change might be made to Mr Dukes' diabetic medication in the near future, it is not a priority at this moment. The pneumonia may even have precipitated Mr Dukes' poor glycaemic control and so his regime may not need changed after he has recovered.
It would be wise to make contact with HDU because Mr Dukes' satisfies at least one of the \_\_criteria below\_\_:
• ''Osmolality greater than 350 mosmol/kg''
• ''Sodium above 160 mmol/L'' - Although the measured sodium is less than 160 mmol/L in this case, the high glucose means this is measured as a lower than the true value. Corrected sodium can be calculated, taking into account the glucose concentration, and this would be over 160 mmol/L in this case.
• Venous/arterial pH below 7.1
• Hypokalaemia (less than 3.5 mmol/L) or hyperkalaemia (more than 6 mmol/L) on admission
• Glasgow Coma Scale (GCS) less than 12 or abnormal
AVPU (Alert, Voice, Pain, Unresponsive) scale
• ''Oxygen saturation below 92% on air''
• Systolic blood pressure below 90 mmHg
• Pulse over 100 or below 60 bpm
• ''Urine output less than 0.5 ml/kg/hr''
• Serum creatinine > 200 µmol/L
• Hypothermia
• Macrovascular event such as myocardial infarction or stroke
• ''Other serious co-morbidity - Mr Dukes' pneumonia.''
You therefore make a call to HDU and perform a perfect SBAR - they will be down to assess him shortly. You prescribe enoxaparin as thromboprophylaxis on Mr Dukes' kardex, before [[reconciling his regular medications->kardex]].
<<if $thrombo>> <<set $nextmanagement +=1>> <</if>>
<<if $foot>> <<set $nextmanagement +=1>> <</if>>
<<if $regimen>> <<else>> <<set $nextmanagement +=1>> <</if>>
<<if $HDU>> <<set $nextmanagement +=1>> <</if>>And with that, Dr $doctor, all you need to do is attend the handover and finish your shift!
\*Mr Dukes' U&Es will be measured regularly to decide on potassium supplementation.
\*Other routine measurements such as BP and urine output will also be closely measured.
\*His serum osmolality will regularly be calculated to ensure satisfactory treatment of his HHS, with fluid adjustments if necessary. Since plasma sodium concentration is influenced by the glucose concentration and the fluid this draws with it, it can actually rise with treatment as the glucose falls - this is not necessarily an indication for hypotonic saline. A corrected sodium can be calculated by adding 2.4mmol/L of sodium for every 5.5mmol/L of extra glucose. This calculated value should fall with treatment.
\*A fixed rate insulin infusion may be started when fluid replacement alone fails to bring blood glucose down - this will be stopped when Mr Dukes is eating normally and is able to take his normal subcutaneous insuln.
\*Early mobilisation is important to reduce the risk of thrombotic events. Replacement of phosphates and other electrolytes/vitamins may be necessary to prevent re-feeding syndrome.
\*A follow up chest x-ray will be organised 6-8 weeks post discharge, assuming satisfactory recovery from the pneumonia.
You return home with the intention of doing some reading on HHS management...
[[Several days pass]]...You bump into your freind and fellow FY2, Dr Dolittle. Mr Dukes is now under his care. He has made excellent progress in recovering from his initial presentation, but is now due to get a colonoscopy. ''Mr Dukes is going to go home and come in as an outpatient''. He would like advice on how to adjust his medication but Dr Dolittle skipped his Diabetes Acute Care Day at university and so asked for your help.
You go and see Mr Dukes:
<video src="videos/breakfastafterendoscopy.mp4" width="640" height="480" controls></video>
You ask him why he's getting the colonoscopy:
<video src="videos/needendoscopy.mp4" width="640" height="480" controls></video>
He also tells you that he's been quite constipated lately, which the doctor said might have made his confusion and diabetic control worse before his admission.
Other than giving him a helpful leaflet with clear instructions on what to do prior to his colonscopy, what should you advise he does ''on the day before''?
Drink sugar-free, clear fluid on the day before <<radiobutton "$sugar" "wrong">>
Drink sugary, clear fluid on the day before <<radiobutton "$sugar" "correct">>
You have a [[quick read]] of the information sheet first so you don't give him the wrong information.
<<if $sugar eq "correct">> <<set $endoscopyadvice +=1>> You were right! Mr Dukes should drink clear, sugary drinks on the day before to make up for the fact that he isn't eating. <</if>> <<if $sugar neq "correct">> Mr Dukes should drink clear, sugary drinks on the day before to make up for the fact that he isn't eating. <</if>>
Medication alteration before colonoscopy is fairly complex and protocols vary slightly between health boards. It is, however, important to grasp basics of needing circulating insulin, encouraging regular blood glucose measurements, and encouraging the patient to drink clear, sugary fluids.
Dr Dolittle asks how you would manage his diabetic medications on the day of the procedure (all diabetic patients should have their procedure performed in the morning). ''Assume his renal function has normalised''.
<<if $nationalguidance eq "3">><img src="pictures/colonoscopy1.jpg" width="500" height="500" alt="suitability">
<img src="pictures/colonoscopy2.jpg" width="500" height="500" alt="suitability"><</if>>
How would you alter his metformin and Humulin M3 on the morning of his colonoscopy?
Take AM metformin as normal <<radiobutton "$metformin" "correct">>
Omit AM metformin <<radiobutton "$metformin" "wrong">>
Take AM Humulin M3 as normal <<radiobutton "$humulin" "wrong">>
Take half of the AM Humulin M3 dose <<radiobutton "$humulin" "correct">>
[[Check answers with senior]] OR [[Check national guidance first->quick read]]
<<set $nationalguidance = 3>><video src="videos/cancer.mp4" width="640" height="480" controls></video>
Although Mr Dukes is scheduled for a morning operation, he will likely miss lunch as well. Dr Dolittle asks how his insulin regime should be adjusted.
First of all, should Mr Dukes be placed on a variable rate insulin infusion?
Mr Dukes should be started on a variable rate insulin infusion <<radiobutton "$sliding" "correct">>
Mr Dukes does not require a variable rate insulin infusion and should take half of his usual Humulin M3 the night before <<radiobutton "$sliding" "wrong">>
[[Next]]
<img src="pictures/suitability.jpg" width="500" height="500" alt="suitability">
<<if $sliding eq "correct">> <<set $variablerateanswer +=1>> Yes! Mr Dukes will be fasted for more than 12 hours and miss two or more meals and so should be placed on a variable rate insulin infusion. His subcutaneous insulin and oral antidiabetic drugs should be stopped. <</if>> <<if $sliding neq "correct">> Mr Dukes will be fasted for more than 12 hours and miss two or more meals and so should be placed on a variable rate insulin infusion. His subcutaneous insulin should be stopped (unless it was long-acting insulin). His oral antidiabetic drugs should also be stopped. <</if>>
Mr Dukes has his operation and is recovering remarkably well. When should his variable rate insulin infusion be stopped?
After dinner, ''with'' subcutaneous insulin administration, there is no need to check Mr Dukes' U&Es beforehand <<radiobutton "$scale" "wrong">>
After dinner, ''with'' subcutaneous insulin administration, after Mr Dukes' U&Es have been checked <<radiobutton "$scale" "wrong">>
After dinner, ''after'' subcutaneous insulin administration, there is no need to check Mr Dukes' U&Es beforehand <<radiobutton "$scale" "wrong">>
After dinner, ''after'' subcutaneous insulin administration, after Mr Dukes' U&Es have been checked <<radiobutton "$scale" "correct">>
You'd better [[double check]]...
<<if $scale eq "correct">> <<set $afteroptotal +=1>> Yes! <</if>> Mr Dukes' biochemistry should be checked beforehand in case of, for example, hyperkalaemia. IV insulin has a half-life of just over two minutes and so it is important to have subcutaneous insulin on board before 30 minutes before this is stopped. It is also important that the patient has eaten a meal and so should not become hypoglycaemic. Oral antidiabetic drugs can now also be started. Note that this is all advice for a mixed-insulin. Basal-bolus regimens follow the same principle of needing S/C insulin on board before IV infusions are stopped.
You return to check on Mr Dukes the next day:
<video src="videos/cancerallclear.mp4" width="640" height="480" controls></video>
You are confused by the fact that he has never changed clothes on any of his admissions, but neveretheless delighted to hear his news!
[[You go home to reflect on the case as a whole]]...<<set $endoanswer = ($endoscopyadvice + $e1 + $e2)>> Dr $doctor, over the course of this case you've managed to cover:
\* What HHS is, how it presents, how it differs from DKA, and the important aspects of its management. This includes learning how to calcuate osmolality and corrected sodium.
\* Key points in switching a patient back to subcutaneous insulin, and how to manage their diabetic medication for colonoscopy and surgery.
\* Safe prescribing, an important GMC outcome for graduates.
\* Using an A-E approach to identify and treat any problems as you find them in an acutely unwell patient. In real life, many patients will have more than one problem - you might have missed the pneumonia if you had simply started treating the hyperglycaemia!
\* Some clinically relevant results interpreation on ABG, ECG, and chest x ray.
<<nobr>>
<<set $final = ($amt4 + $diagnosisanswer + $otherpresentation + $initialfluids + $CXRtotal + $ABGtotal + $ECGtotal + $previousmianswer + $ABGinterpretation + $pneumoniaanswer + $curb65answer + $checkallergy + $medrec + $endoscopyadvice + $variablerateanswer + $afteroptotal + $osmolalitytotal + $nextmanagement + $kanswer + $goodbloodanswers - $badbloodanswers + $e1 + $e2)>>
<</nobr>>
\_\_Breakdown of scores\_\_
Total points for AMT 4 - $amt4 / 3
Total points for correct blood tests - $goodbloodanswers / 9
Total points ''deducted'' for unnecessary blood tests - $badbloodanswers / 2
Total points for HHS diagnosis - $diagnosisanswer / 1
Total points for knowing there was another condition to diagnose - $otherpresentation / 1
Total points for prescribing the correct fluids - $initialfluids / 1
Total points for ordering a chest X-ray - $CXRtotal / 1
Total points for performing an ABG - $ABGtotal / 1
Total points for determining the acid-base balance - $ABGinterpretation / 1
Total points for ECG interpretation - $ECGtotal / 5
Total points for noticing evidence of a previous MI - $previousmianswer / 1
Total points for diagnosing pneumonia - $pneumoniaanswer / 1
Total points for calculating the CURB-65 - $curb65answer / 1
Total points for checking for antibiotic allergies and interactions - $checkallergy / 1
Total points for calculating the osmolality - $osmolalitytotal / 2
Total points for correct K+ replacement - $kanswer / 1
Total points for next management steps - $nextmanagement / 4
Total points for medicine reconciliation - $medrec / 4
Tota points for endoscopy advice - $endoanswer / 3
Total points for pre-op insulin management - $variablerateanswer / 1
Total points for safely converting IV to SC insulin - $afteroptotal / 1
Total = <<print $final>>/ 43
[[Finally, let's see if you've earned any awards!]]You finish your A-E examantion by performing an abdominal examanation and examining Mr Dukes from head to toe methodically. You find nothing of note.
[[Carry on...->Available]]You are an FY2 working in a busy medical receiving unit. It's so busy in fact that your name has been forgotten...
Remind me again, Doctor... <<textbox "$doctor" "Surname">>
[[Continue->Mr Dukes]]
<<audio "backgroundhospital" volume 0.05 loop play>><img src="pictures/curb.jpg" width="400" height="400" alt="curb">
<<if $curb eq "correct">> <<set $curb65answer +=1>> Well done - Mr Dukes' CURB-65 score is 3. <</if>> <<if $curb eq "wrong">> Not quite - Mr Dukes' CURB-65 score is 3. <</if>>
Most protocols also advise that a severe pneumonia can be defined by a positive SIRS/qSOFA criteria.
Dr Jandhu would like you to fill out the kardex //as soon as possible//. What will you prescribe?
Clarithromycin and amoxicillin <<radiobutton "$prescribe" "wrong">>
Clarithromycin and co-amoxiclav <<radiobutton "$prescribe" "wrong">>
Levofloxacin monotherapy <<radiobutton "$prescribe" "wrong">>
Need more information <<radiobutton "$prescribe" "correct">>
[[Continue->kardex1]]<<if $boyle eq 1>><img src="pictures/boyle.jpg" width="60" height="80" alt="boyle">''You've earned the Dr James Boyle Award!'' Yes, it's an award for excellence in diabetes management from the ''most'' excellent in diabetes management. No, Dr Boyle is definitely not telling me to write this. Your prize? Meeting Dr Boyle ''in person'' at the next academic day (unless it's on Zoom lol).<</if>><<if $boyle neq 1>><img src="pictures/noprize.jpg" width="40" height="40" alt="noprize">''Try playing through again to unlock this award''.<</if>>
<<if $walters eq 1>><img src="pictures/walters.jpg" width="60" height="70" alt="walters"> ''You've earned the Professor Walters Award for safe prescribing!'' This award comes with the special prize of a signed BNF from Professor Walters, the champion of safe prescribing. That's what we would say if we had any BNFs to give away.<</if>><<if $walters neq 1>><img src="pictures/noprize.jpg" width="40" height="40" alt="noprize">''Try playing through again to unlock this award''.<</if>>
<<if $otherpresentation eq 1>><img src="pictures/hickam.jpg" width="60" height="80" alt="prize 3"> ''You've earned the Hickam's Dictum award! Remember, the patient can have as many diagnoses as they please.<</if>><<if $otherpresentation neq 1>><img src="pictures/noprize.jpg" width="40" height="40" alt="noprize">''Try playing through again to unlock this award''.<</if>>
Thanks for playing the game!
[[Restart]]
<<audio "backgroundhospital" stop>>
<<audio "sugar" loop play>>You ask if his breathlessness is new or longstanding...
<video src="videos/breathless.mp4" width="640" height="480" controls></video>
He won't elaborate on this answer...it certainly doesn't sound like this was a sudden onset of breathlessness so this might help narrow down your differentials.
What are your differentials for his \_\_breathlessness\_\_?
<<textbox "$differentials1" "">>
<<textbox "$differentials2" "">>
<<textbox "$differentials3" "">>
<<set $history4 =1>>
<<if $history1 eq 1 and $history2 eq 1 and $history3 eq 1 and $history4 eq 1>> [[Continue History->Cut to the chase]] <</if>>
<<if $history1 neq 1 or $history2 neq 1 or $history3 neq 1 or $history4 neq 1>> [[Continue History->Let's see]] <</if>>
<<cacheaudio "breathsounds" "music/breathsounds2.mp3">>
<<cacheaudio "heartsounds" "music/heartsounds2.mp3">>
<<cacheaudio "backgroundhospital" "music/backgroundhospital2.mp3">>
<<cacheaudio "sugar" "music/sugar2.mp3">>
<<set $walters = 0>>
<<set $boyle = 0>>
<<set $jpl = 0>>
<<set $goodbloodanswers = 0>>
<<set $badbloodanswers = 0>>
<<set $amt4 = 0>>
<<set $diagnosisanswer = 0>>
<<set $otherpresentation = 0>>
<<set $initialfluids = 0>>
<<set $CXRtotal = 1>>
<<set $ABGtotal = 1>>
<<set $ECGtotal = 0>>
<<set $previousmianswer = 0>>
<<set $ABGinterpretation = 0>>
<<set $pneumoniaanswer = 0>>
<<set $curb65answer = 0>>
<<set $checkallergy = 0>>
<<set $medrec = 0>>
<<set $endoscopyadvice = 0>>
<<set $variablerateanswer = 0>>
<<set $afteroptotal = 0>>
<<set $osmolalitytotal = 2>>
<<set $nextmanagement = 0>>
<<set $kanswer = 0>>
<<set $e1 = 0>>
<<set $e2 = 0>>
<<set $e3 = 0>>
<<set $osmole = 0>>
<<if $metformin eq "correct">> Correct, Dr $doctor, you would give him his AM metformin on the day as normal. <<set $e1 = 1>><</if>> <<if $metformin neq "correct">> Not quite, Dr $doctor, you would give him his AM metformin on the day as normal.<</if>>
<<if $humulin eq "correct">> Correct, Dr $doctor, you would halve his AM dose of Humulin M3 on the day. <<set $e2 = 1>> <</if>> <<if $humulin neq "correct">> Not quite, Dr $doctor, you would halve his AM dose of Humulin M3 on the day of the procedure.<</if>>
You wish Mr Dukes the best of luck, but learn he is an inpatient again [[several weeks later]]...Welcome to the <span class="greentext"> Acute Care Virtual Patient Simulation - Scenario 3</span>!
This was developed by Dr Nat Quail under the close supervision of Dr James Boyle\*. We would like to acknowledge the generous grant received from the Learning and Teaching Development Fund, which enabled us to pay for actors and equipment.
We hope that this game will enable you to learn more about the management of specific conditions and acutely ill patients in a fun\*\* and safe manner.
[[Instructions]]
So it's his fault if anything goes worng\*
May not be fun\*\*Just a few quick notes before you start:
\*If the question is multi-choice, make sure you finalise your answers before ticking the box. Some multi-choice questions require more than one answer.
\*For most questions you will be scored on how you answer. If you pick more choices than are necessary (for example, requesting unnecessary blood tests) then you may lose points.
\*Click on the coloured links within passages to continue
\*''Watch out for your web-browser trying to autocorrect free-text answers''
[[Introduction]]
<<set $now to new Date()>>
<<set $start = $now>>
<<script>>
state.restart();
<<endscript>>
