# Supplementary material for: Twine virtual patient games as an online resource for undergraduate diabetes acute care education
Source: BMC Med Educ. 2023 Jun 7;23:417. doi: 10.1186/s12909-023-04231-2 (PMC10244842; doi:10.1186/s12909-023-04231-2)
Supplement: Supplementary file 4 — Supplementary Material 4: Multiple Choice Questions and Confidence Questionnaires [file 12909_2023_4231_MOESM4_ESM.docx]

| EvaSys | **Diabetes Acute Care Day Formative Examination of Knowledge And Confidence** | 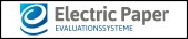 |
| --- | --- | --- |
| University of Glasgow Or J. Boyle  Undergraduate Medical School Formative Examination of Knowledge and Confidence | | |



# STUDENT NAME



- 1. **REGISTRATION NUMBER**




 ^



^

 ^



^

 ^



^

 ^



^

 ^



^

 ^



^

 ^



^







# CONSENT

I consent to taking part in the study 'The use of serious gaming in the flipped classroom approach to teaching diabetes acute care to medical students' and agree to my data being used as described in Participant Information Sheet V3.0 - please mark box on right
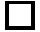



A 59 year old man with Type 2 diabetes treated with Humulin M3 twice daily attends the surgical outpatient clinic. You are arranging for him to attend for an outpatient colonoscopy on a morning list

# What is the most appropriate immediate management?



Advise him to take his usual dose of insulin on the morning



Admit the day before procedure for a variable rate insulin infusion from midnight



Commence on a variable rate insulin infusion on the morning

Advise him to take 75% of his usual insulin on the morning



Advise him to take 50% of his usual dose of insulin on the morning



# After the procedure the man asks you to review his blood sugar diary before he goes home


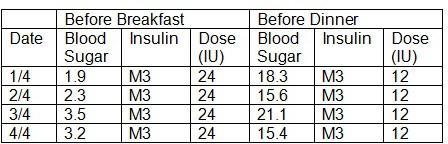


- 1. **What is the most appropriate advice?** Stop breakfast M3 and increase dinner M3 dose



Reduce breakfast M3 dose and continue dinner M3 dose



Reduce breakfast M3 and increase dinner M3 doses

Stop breakfast M3 dose and reduce dinner M3 dose

Continue breakfast M3 dose and reduce dinner M3 dose




A 65-year-old man with Type 1 diabetes is admitted and has emergency surgery managed and you are asked to prescribe a variable rate insulin infusion. His insulin regimen is normally Novomix 30 twice daily.

# Investigations:

Haemoglobin A1c **76** mmol/mol (20-42)

serum sodium **135** mmol/L (137-144)

serum potassium **4.5** mmol/L (3.5-4.9)

serum urea **6** mmol/L (2.5-7.0)

serum creatinine **67** µmol/L (60-110)

estimated glomerular filtration rate (MDRD) **>60** mL/min (>60) serum glucose **12** mmol/l (3.5-5.5)

serum bicarbonate **25** mmol/L (20-28)

# What is the most appropriate prescription (insulin syringe + 5 hourly fluid infusion)



100 units IV actrapid in 50mil dextrose syringe + IV 5% dextrose



50 units IV actrapid in 50mls saline syringe + IV 5% dextrose



50 units IV actrapid in 50mls dextrose + IV 0.9% saline and 20mmol KCL

100 units IV actrapid in 50mls saline syringe + IV 5% dextrose



50 units IV actrapid in 50mls saline + IV 5% dextrose and 20mmol KCL



# Postoperative recovery is swift. His insulin regimen is normally Novomix 30 twice daily. He had breakfast four hours ago.

- 1. **What is the most appropriate management?**

Give usual s/c insulin dose now and stop intravenous insulin immediately

Give usual s/c insulin dose now and stop intravenous insulin 30 minutes later

Give usual s/c insulin dose with lunch and stop intravenous insulin immediately

Give usual s/c insulin dose with evening meal, stop intravenous insulin 30minutes later

Give usual s/c insulin dose with lunch, stop intravenous insulin 30minutes later

4. **QUESTION 3**

A 28 year old woman is referred from A&E with a short history of polydipsia, polyuria and weight loss.

On examination she looks well.

# Investigations:

Haemoglobin A1c **106** mmol/mol (20-42)

serum sodium **133** mmol/L (137-144)

serum potassium **3.5** mmol/L (3.5-4.9)

serum urea **7.0** mmol/L (2.5-7.0)

serum creatinine **71** µmol/L (60-110)

estimated glomerular filtration rate (MDRD) **>60** mL/min (>60) serum glucose **14** mmol/l (3.5-5.5)

serum bicarbonate **22** mmol/L (20-28)

capillary ketones **3.2** mmol/l (<0.6)

urinalysis**: 4+ glucose, 1+ ketones**

- 1. **What is the most likely diagnosis?** Maturity onset Diabetes of the Young

Diabetes Ketoacidosis Hyperosmolar Hyperglycaemia State

Diabetes Ketonaemia Type 2 diabetes

# What is the most appropriate treatment?

Gliclazide Diabetes Ketoacidosis Pathway Variable rate insulin infusion

Hyperosmolar Hyperglycaemia State Guideline

Subcutaneous insulin

An 89 year old man is referred from A&E. He had a fall. He has a history of Type 2 diabetes managed by Gliclazide 80mg od and Metformin 1g bd. On examination he is unconscious.

# Investigations:

Haemoglobin A1c **40** mmol/mol (20-42) serum sodium **135** mmol/L (137-144)

serum potassium **4.5** mmol/L (3.5-4.9)

serum urea **16** mmol/L (2.5-7.0)

serum creatinine **204** µmol/L (60-110)

estimated glomerular filtration rate (MDRD) **18** mL/min (>60) serum glucose **1.9** mmol/l (3.5-5.5)

serum bicarbonate **24** mmol/L (20-28)

# What is the most appropriate immediate management?

15-20 grams fast acting carbohydrate (100mls Lucozade)

2 biscuits

100mls of 50% V glucose 200mls of V 10% V glucose

2mg Glucagon M

# What is the most appropriate ongoing management?

Stop metformin and continue gliclazide

Reduce gliclazide and reduce metformin

Stop metformin and stop gliclazide

Stop gliclazide and reduce metformin

Stop gliclazide and continue metformin

6. **QUESTION 5**

A 50 year old man is referred from A&E having become unwell over a few days. On examination he appears very dehydrated.

# Investigations:

Haemoglobin A1c **52** mmol/mol (20-42) serum sodium **155** mmol/L (137-144)

serum potassium **3.6** mmol/L (3.5-4.9)

serum urea **19** mmol/L (2.5-7.0)

serum creatinine **120** µmol/L (60-110)

estimated glomerular filtration rate (MDRD) **>60** mL/min (>60) serum glucose **45** mmol/l (3.5-5.5)

serum bicarbonate **18** mmol/L (20-28)

capillary ketones **0.5** mmol/l (<0.6)

urinalysis: **4+ glucose**

# What is the most likely diagnosis?

Pancreatic pathology Diabetes Ketoacidosis Diabetes Ketonaemia

Hyperosmolar Hyperglycaemia State

Type 1 diabetes

# What is the most appropriate immediate management?

Gliclazide Diabetes Ketoacidosis Pathway Variable rate insulin infusion

Hyperosmolar Hyperglycaemia State Guideline

Subcutaneous insulin

# PLEASE TURN OVER TO QUICKLY COMPLETE CONFIDENCE ASSESSMENT

In this part of the assessment the confidence of respondents will be assessed. 0 means you are 'not confident' and 100 means you are fully confident in most cases. Please mark the box to indicate your level of confidence for each of the following 10 questions.

- 1. **Making a diagnosis of diabetes?**
  2. **Diagnosing and managing hypoglycaemia in hospital?**
  3. **Diagnosing and managing DKA in hospital?**
  4. **Diagnosing and managing HHS in hospital?**
  5. **Interpreting capillary blood glucose result charts in hospital?**
  6. **Managing patients on SC insulin therapy in hospital?**
  7. **Managing patients on IV insulin in hospital?**
  8. **Managing patients on oral hypoglycaemic agents in hospital?**
  9. **Prescribing IV fluids for patients with diabetes?**
  10. **Altering diabetes therapy prior to surgery/other procedures?**

File name: Supplementary Material 4

File format: .docx

Title of data: Multiple Choice Questions and Confidence Questionnaires

Description of data: Multiple Choice Questions and Confidence Questionnaires.
