# Supplementary material for: Twine virtual patient games as an online resource for undergraduate diabetes acute care education
Source: BMC Med Educ. 2023 Jun 7;23:417. doi: 10.1186/s12909-023-04231-2 (PMC10244842; doi:10.1186/s12909-023-04231-2)
Supplement: Supplementary file 5 — Supplementary Material 5: Resources Questionnaires [file 12909_2023_4231_MOESM5_ESM.docx]

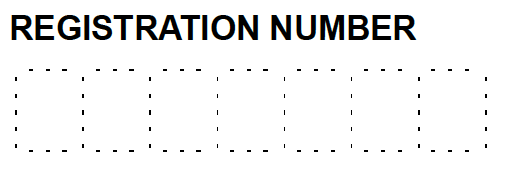


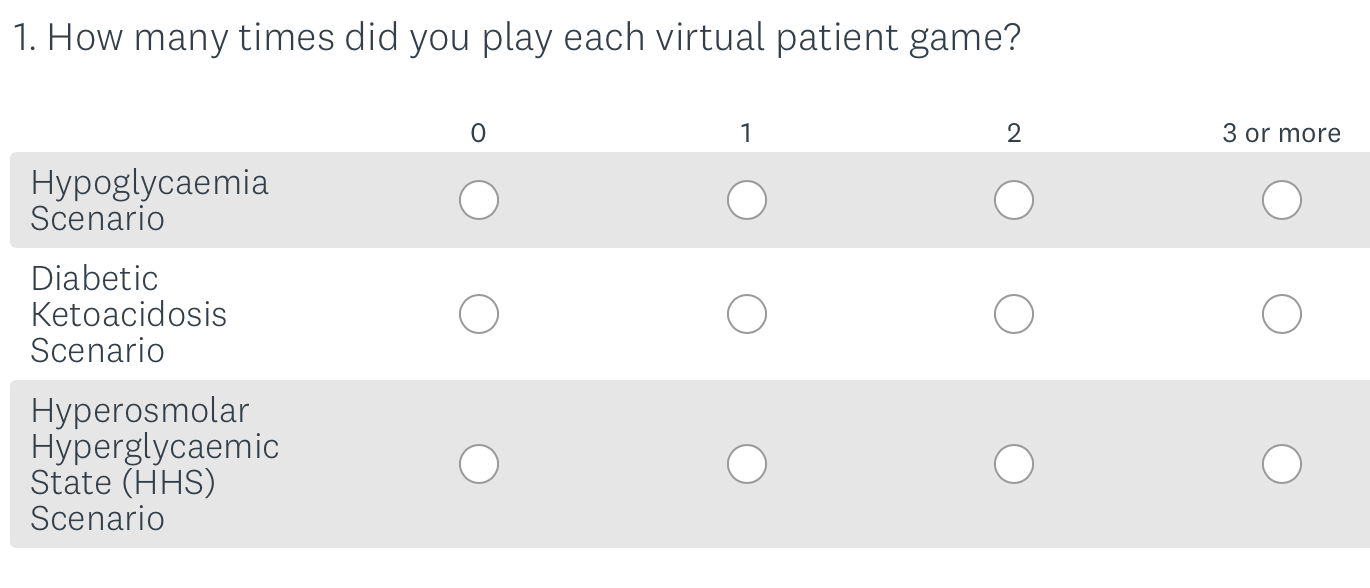


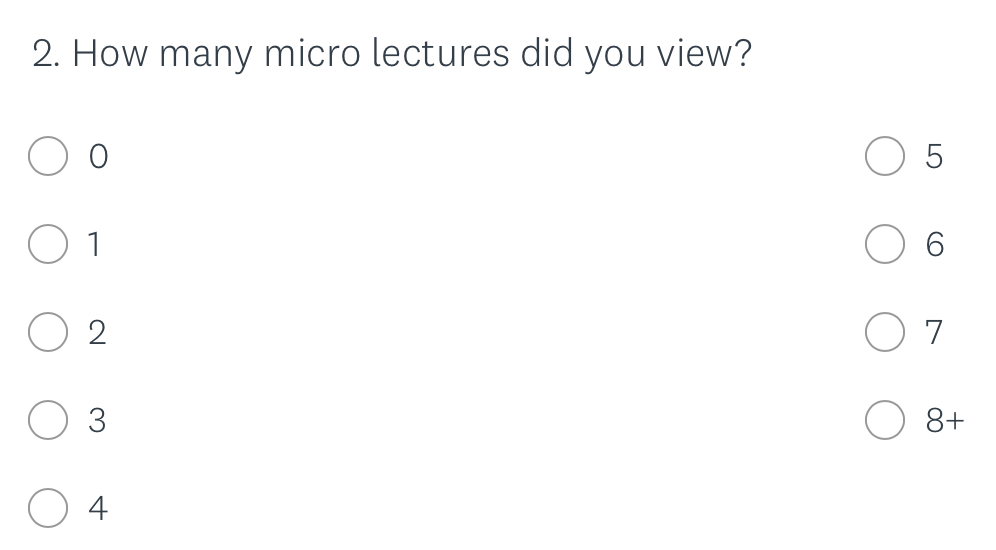

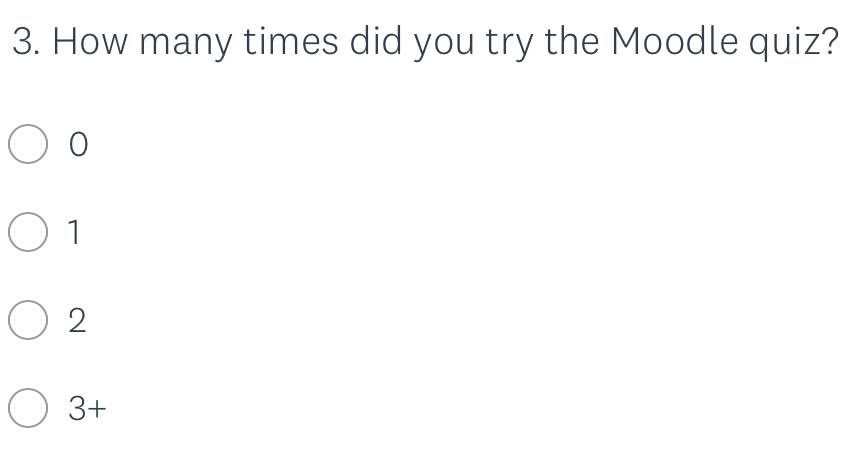

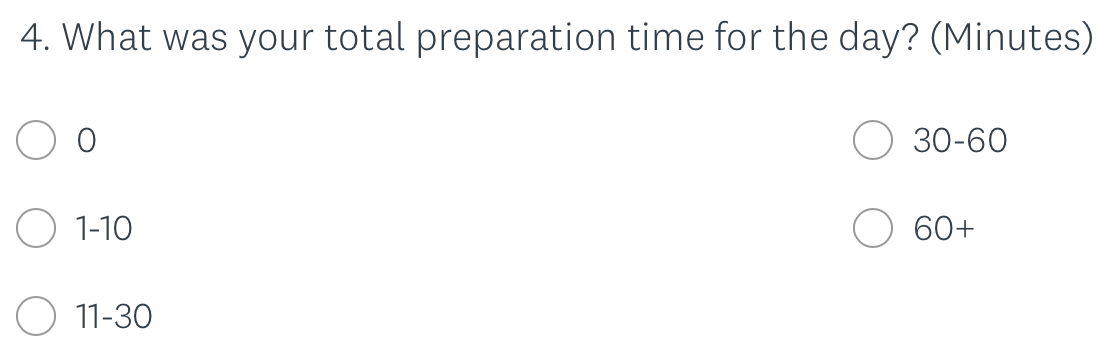


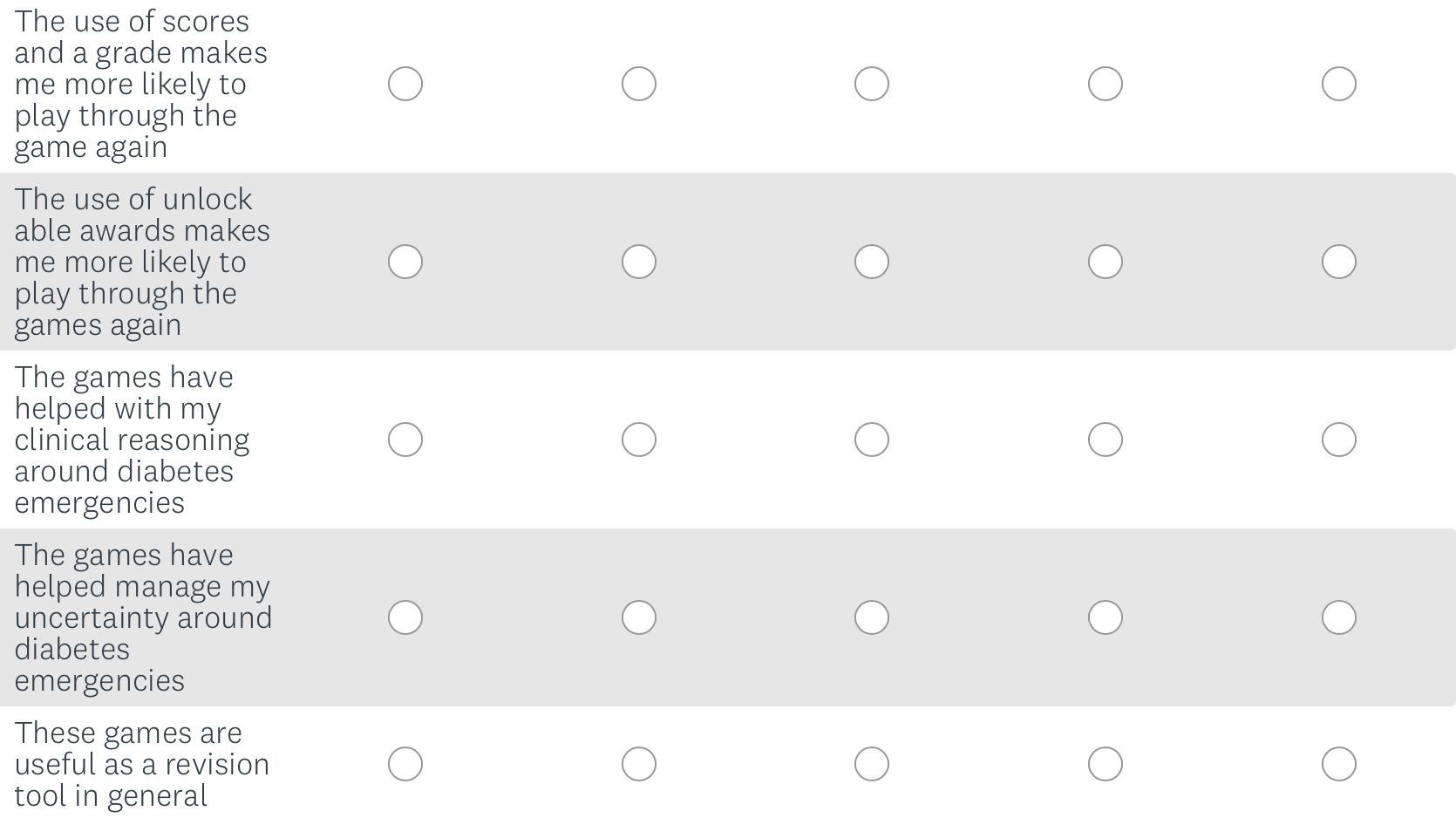

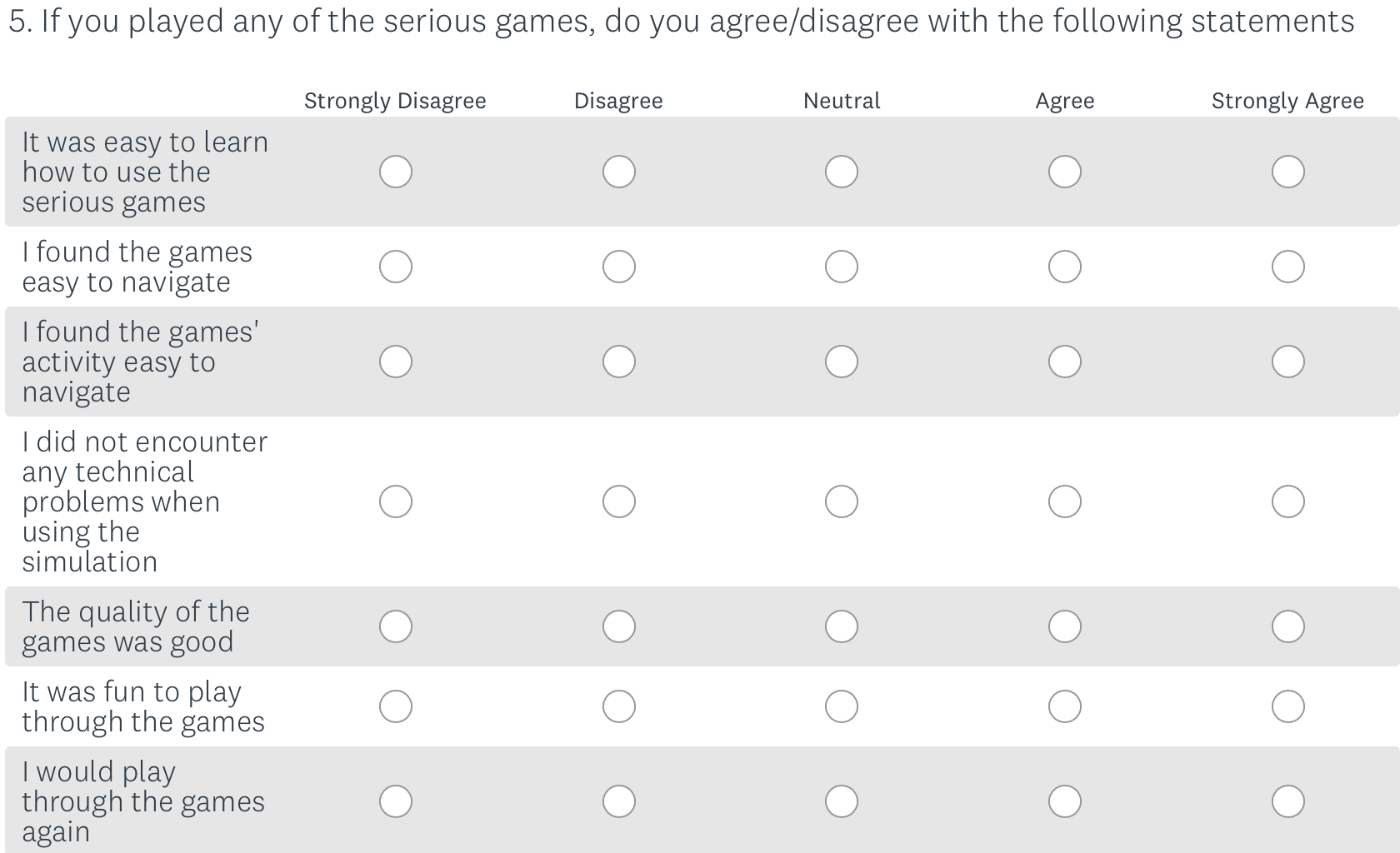


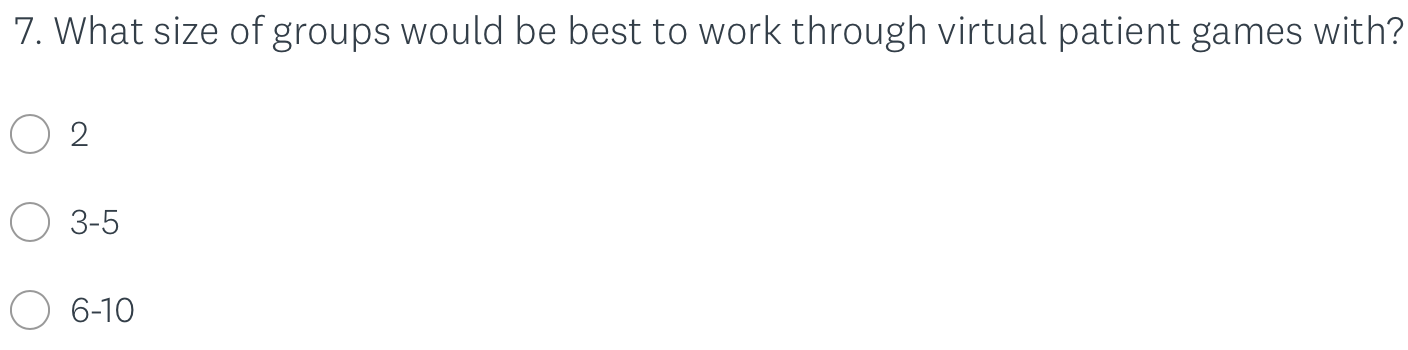

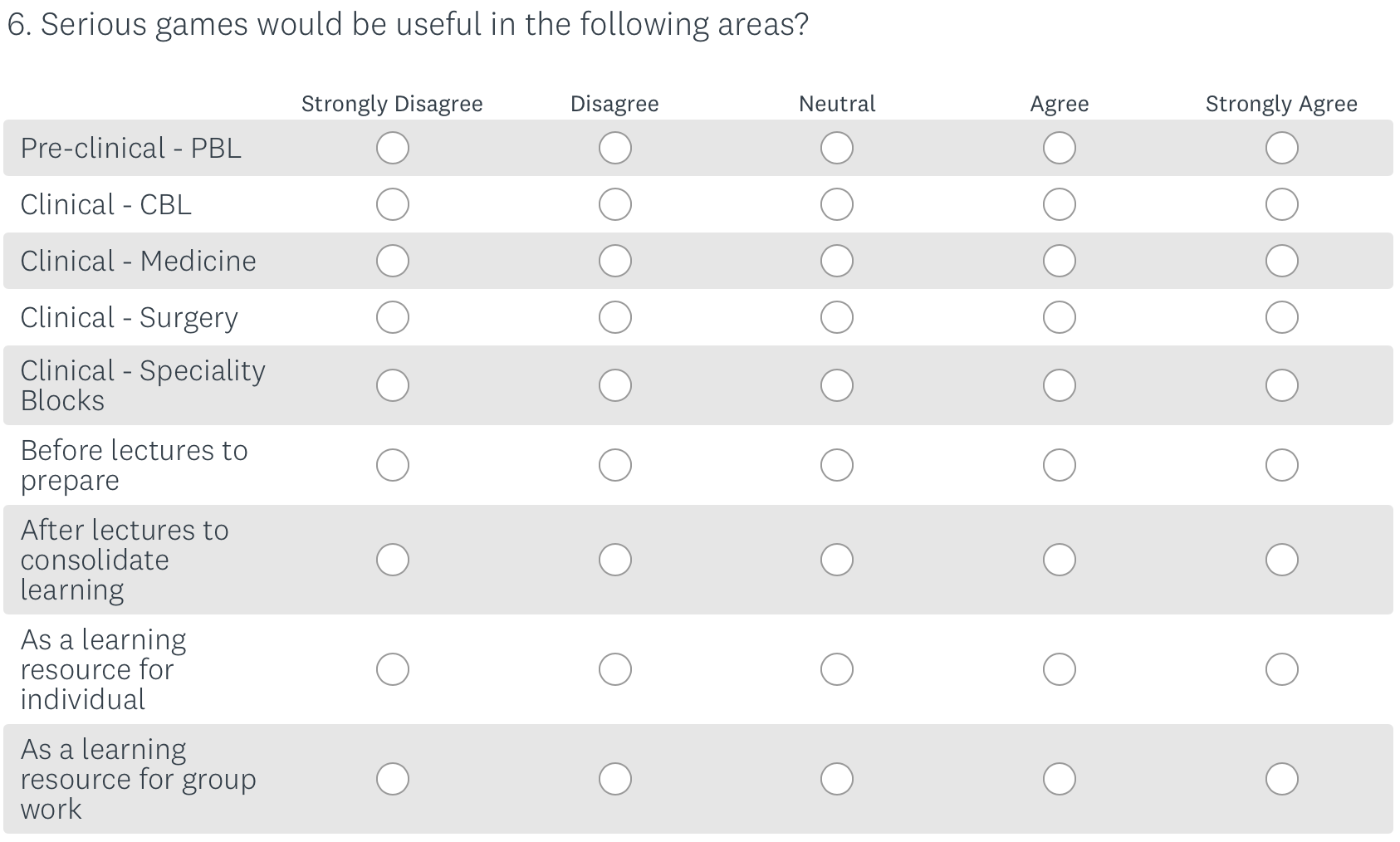


File name: Supplementary Material 5

File format: .docx

Title of data: Online Resources Questionnaires

Description of data: Online Resources Questionnaires.
