# Supplementary material for: Twine virtual patient games as an online resource for undergraduate diabetes acute care education
Source: BMC Med Educ. 2023 Jun 7;23:417. doi: 10.1186/s12909-023-04231-2 (PMC10244842; doi:10.1186/s12909-023-04231-2)
Supplement: Supplementary file 6 — Supplementary Material 6: Virtual Patient Blueprint Game [file 12909_2023_4231_MOESM6_ESM.zip › 12909_2023_4231_MOESM6_ESM/index.html]

Example Game BMC


JavaScript must be enabled to play.

Browser lacks capabilities required to play.

Upgrade or switch to another browser.

Loading…

 Welcome to the Twine virtual patient COVID-19 game!
This was developed by Dr Nathaniel Quail as a blueprint for virtual patient games. Please see the supporting document.
The clinical content in this scenario is based on current practice (October 2021) in Scotland. The purpose of this game is to act as a blueprint rather than provide any clinical teaching.
Make sure your audio is on - you should hear music in the next passage.
[[Introduction]]
You are a Doctor working in a busy GP referral department at a Glasgow hospital ... so busy in fact that your name has been forgotten ... remind me again Doctor...<<textbox "$surname" "Surname">>
[[Continue->Mr Quail]]
<<audio "background" volume 0.4 loop play>><<nobr>>
<<set $storyprogress = 1>>
<<audio "background" stop>>
<<if $timer eq "0">>
<<set $start to new Date()>>
Mr Quail, a (very youthful) 54-year-old journalist, has presented with shortness of breath. He says his partner tested positive for coronavirus last week and is concerned he now has it. Mr Quail's past medical history includes asthma (never required hospital admission), hypertension, type 2 diabetes, and osteoarthritis. He has not been vaccinated against COVID-19. His initial observations are available to view on the sidebar.
<</if>>
<</nobr>>
What specific questions would you like to ask regarding potential symptoms of COVID-19?
Keep coming back to this page to ask about as many relevant symptoms as you can think of.''
<<textbox "$symptom" "">>
[[Ask about symptom]]
<<set $symptomcheck = 0>><<nobr>>
<<set $timer = 1>>
<<set $symptom to $symptom.toLowerCase()>>
<<set $symptom to $symptom.trim()>>
<<if $symptom .includes("cough") or $symptom .includes("phlegm") or $symptom .includes("mucous") or $symptom .includes("mucus") or $symptom .includes("spit") or $symptom .includes("coryz") or $symptom .includes("nose")>>
<video src="videos/cough.mp4" width="640" height="480" controls> </video>
<<set $cough = 1>>
<<set $symptomcheck = 1>>
<</if>>
<<if $symptom .includes("sob") or $symptom .includes("short") or $symptom .includes("breath")>>
<video src="videos/sob.mp4" width="640" height="480" controls> </video>
<<set $sob = 1>>
<<set $symptomcheck = 1>>
<</if>>
<<if $symptom .includes("blood") or $symptom .includes("haem")>>
<video src="videos/haemoptysis.mp4" width="640" height="480" controls> </video>
<<set $haemoptysis = 1>>
<<set $symptomcheck = 1>>
<</if>>
<<if $symptom .includes("fever") or $symptom .includes("pyrex") or $symptom .includes("tempera") or $symptom .includes("rigor") or $symptom .includes("shiver")>>
<video src="videos/pyrexia.mp4" width="640" height="480" controls> </video> <<set $pyrexia = 1>>
<<set $symptomcheck = 1>>
<</if>>
<<if $symptom .includes("taste") or $symptom .includes("smell") or $symptom .includes ("anosmia") or $symptom .includes("ageusia")>>
<video src="videos/taste.mp4" width="640" height="480" controls> </video>
<<set $taste = 1>>
<<set $symptomcheck = 1>> <</if>>
<<if $symptom .includes ("vomit") or $symptom .includes ("naus") or $symptom .includes ("sick") or $symptom .includes ("appeti")>><video src="videos/nandv.mp4" width="640" height="480" controls> </video>
<<set $vomit = 1>>
<<set $symptomcheck = 1>>
<</if>>
<<if $symptom .includes ("diar") or $symptom .includes ("constipation") or $symptom .includes ("stool") or $symptom .includes ("bowel") or $symptom .includes ("defecat") or $symptom .includes ("toilet")>> <video src="videos/diarrhoea.mp4" width="640" height="480" controls> </video>
<<set $diarrhoea = 1>>
<<set $symptomcheck = 1>>
<</if>>
<<if $symptom .includes ("myalg") or $symptom .includes ("muscle") or $symptom .includes ("weak")>>
<video src="videos/myalgia.mp4" width="640" height="480" controls> </video> <<set $myalgia = 1>>
<<set $symptomcheck = 1>>
<</if>>
<<if $symptom .includes ("throat") or $symptom .includes ("swallow")>><video src="videos/throat.mp4" width="640" height="480" controls> </video>
<<set $sorethroat = 1>>
<<set $symptomcheck = 1>>
<</if>>
<<if $symptom .includes ("pain") or $symptom .includes ("pleuri") or $symptom .includes ("chest")>><video src="videos/chestpain.mp4" width="640" height="480" controls> </video>
<<set $chestpain = 1>>
<<set $symptomcheck = 1>>
<</if>>
<<if $symptom .includes ("head")>><video src="videos/headache.mp4" width="640" height="480" controls> </video>
<<set $headache = 1>>
<<set $symptomcheck = 1>>
<</if>>
<<if $symptom .includes ("dog") or $symptom .includes ("pet") or $symptom .includes ("animal")>>
<video src="videos/dog.mp4" width="640" height="480" controls> </video>
<<set $symptomcheck = 1>>
<</if>>
<<if $symptomcheck eq "0">> Mr Quail hasn't experienced anything like that, try asking some more questions Dr $surname.<</if>>
<</nobr>>
Remember, Dr $surname, to ask Mr Quail about as many relevant symptoms as you can think of.
[[Ask something else->Mr Quail]]
<<nobr>>
<<set $end to new Date()>>
<<set $duration = (($end-$start)/1000)>>
<<if ($duration > 20) or (($cough + $sob + $haemoptysis + $pyrexia + $taste + $vomit + $diarrhoea + $myalgia + $sorethroat + $chestpain + $headache) > 3 )>>
If you are ready to continue, let's [[examine Mr Quail]]
<</if>>
<</nobr>><<set $storyprogress = 1>>
You perform a thorough A-E assessment. Let's focus on what you find on auscultation of the lung fields. Click on different points of the image to examine the breath sounds.
<<nobr>>
<audio id="vesicular">
<source src="audio/vesicular.mp3" type="audio/mpeg" />
</audio>
<audio id="covid">
<source src="audio/covid.mp3" type="audio/mpeg" />
</audio>
<map name="lungs" id="lungs">
<area target="" coords="192,104,136,141,94,200,80,250,394,260,373,186,344,143,295,109,240,98" shape="poly"
onClick="SugarCube.Dialog.setup('Upper Zones');SugarCube.Dialog.wiki('That sounds normal');SugarCube.Dialog.open();document.getElementById('vesicular').play() "/>
<area target="" coords="73,252,70,392,122,397,194,384,218,323,218,255" shape="poly"
onClick="SugarCube.Dialog.setup('Mid+Lower Zones');SugarCube.Dialog.wiki('That does not sound normal...');SugarCube.Dialog.open();document.getElementById('covid').play() "/>
<area target="" coords="260,257,269,374,338,393,394,380,404,314,395,261" shape="poly" onClick="SugarCube.Dialog.setup('Mid+Lower Zones');SugarCube.Dialog.wiki('That does not sound normal...');SugarCube.Dialog.open();document.getElementById('covid').play() "/>
</map>
<div class="resizable imageMapObserve" style="width: 600px;">
<img usemap="#lungs" alt="lungs" <img src="images/lungs.png">
</div>
<</nobr>>
[[Continue->bloods]]<<set $timer = 0>>
<<set $symptomcheck = 0>>
<<set $sob = 0>>
<<set $cough = 0>>
<<set $pyrexia = 0>>
<<set $taste = 0>>
<<set $vomit = 0>>
<<set $diarrhoea = 0>>
<<set $myalgia = 0>>
<<set $sorethroat = 0>>
<<set $chestpain = 0>>
<<set $headache = 0>>
<<set $haemoptysis = 0>>
<<set $storyprogress = 0>>
<<set $ecg = 0>>
<<set $cxr = 0>>
<<set $medrec = 0>>
<<set $D0 = 0>>
<<set $D1 = 0>>
<<set $D2a = 0>>
<<set $D2b = 0>>
<<set $H0 = 0>>
<<set $H1 = 0>>
<<set $H2 = 0>>
<<set $O0 = 0>>
<<set $O1 = 0>>
<<set $A = 0>>
<<cacheaudio "background" "audio/background.mp3">>
<video src="videos/ending.mp4" width="640" height="480" controls> </video>
<<click "See Credits">><<replace "#credits">>
Video actors - Nathaniel (and Loki) Quail. Kindly filmed by Ailsa Quail. We claim no form of copyright over these.
----------------------------------------------------------------
Background music - Audio by "Hans Müller-Kray", retrieved from https://imslp.org/wiki/Trumpet\_Concerto\_in\_E-flat\_major%2C\_Hob.VIIe:1\_(Haydn%2C\_Joseph) on 20/12/2021. CC: https://creativecommons.org/licenses/by-sa/4.0/
----------------------------------------------------------------
Chest X Ray - Image by "User Hellerhoff", retrieved from https://commons.wikimedia.org/wiki/Category:X-rays\_of\_COVID-19#/media/File:Covid-19-Pneumonie\_gering\_ausgepraegt\_50M\_-\_CR\_pa\_-\_001.jpg on 20/12/2021. CC: https://creativecommons.org/licenses/by-sa/4.0/
----------------------------------------------------------------
Crackles - Audio by "James Heilman", retrieved from https://commons.wikimedia.org/wiki/File:Crackles\_pneumoniaO.ogg on 20/12/2021. CC: https://creativecommons.org/licenses/by-sa/3.0/deed.en
----------------------------------------------------------------
CSS - Modified from Leon's stylesheet - https://twinery.org/forum/discussion/743/leons-stylesheets)
----------------------------------------------------------------
ECG - Image by "Ewingdo", retrieved from: https://commons.wikimedia.org/wiki/Category:ECG\_of\_sinus\_tachycardia#/media/File:ECG\_Sinus\_Tachycardia\_132\_bpm.jpg on 10/03/2023. CC: http://creativecommons.org/licenses/by-sa/4.0/
----------------------------------------------------------------
Image map generated via https://www.image-map.net/
Code modified from https://qjzhvmqlzvoo5lqnrvuhmg.on.drv.tw/UInv/Sample\_Code.html#Clicking%20Parts%20of%20Images (License: MIT, David J. Bradshaw, modified for Twine by HiEv)
----------------------------------------------------------------
Lungs - Image by "Khalid Qaf", retrieved from https://www.flickr.com/photos/dr-kh-qalam/51349530641/in/photolist-2mezLtc-2mezLsf on 20/12/2021. CC: https://creativecommons.org/licenses/by/2.0/
----------------------------------------------------------------
No Trophy - https://commons.wikimedia.org/wiki/Category:Trophy\_icons#/media/File:Cup\_of\_Silver.svg on 20/12/2021. Image is in the public domain.
----------------------------------------------------------------
Trophy - Image by Wikimedia, retrieved from:
Image by Wikimedia, retrieved from: https://commons.wikimedia.org/wiki/Category:Trophy\_icons#/media/File:Cup\_of\_Gold.svg on 20/12/2021. Image is in the public domain.
----------------------------------------------------------------
Vesicular breath sounds - Audio by "TheSimTech", retrieved from http://www.thesimtech.org/audio on 20/12/2021. CC: https://creativecommons.org/licenses/by-sa/4.0/
----------------------------------------------------------------
<</replace>><</click>> <div id="credits"></div>
[[Restart]]
<<audio "background" stop>>As you await a chest X-ray and a point of care COVID test, Nurse Boyle has kindly taken some bloods in every colour tube you could possibly need. Reassuringly, Nurse Boyle has also kindly performed a venous blood gas. This shows pH, bicarbonate, and lactate to be within reference range.
What would you like Nurse Boyle to send to the lab and why? Try not to request unnecessary tests.
Ammonia <<checkbox "$Ammonia" false true unchecked>> <<textbox "$AmmoniaReason" "reason?">>
Amylase <<checkbox "$Amylase" false true unchecked>> <<textbox "$AmylaseReason" "reason?">>
Coagulation screen <<checkbox "$Coag" false true unchecked>> <<textbox "$CoagReason" "reason?">>
Cortisol <<checkbox "$Cortisol" false true unchecked>> <<textbox "$CortisolReason" "reason?">>
CRP <<checkbox "$CRP" false true unchecked>> <<textbox "$CRPReason" "reason?">>
D-dimer <<checkbox "$Ddimer" false true unchecked>> <<textbox "$DdimerReason" "reason?">>
FBC <<checkbox "$FBC" false true unchecked>> <<textbox "$FBCreason" "reason?">>
Glucose <<checkbox "$Glucose" false true unchecked>> <<textbox "$GlucoseReason" "reason?">>
LFTs <<checkbox "$LFT" false true unchecked>> <<textbox "$LFTReason" "reason?">>
Troponin <<checkbox "$Troponin" false true unchecked>> <<textbox "$TroponinReason" "reason?">>
U&Es <<checkbox "$UE" false true unchecked>> <<textbox "$UEReason" "reason?">>
[[You double check with your senior before requesting the bloods->senior check]]
<<nobr>>
<<if $storyprogress eq "1">> <<click "View Observations">><<replace "#obs">>[img[images/NEWS1.png]]<</replace>><</click>> <div id="obs"></div> <</if>>
<<if $storyprogress eq "2">> <<click "View Observations">><<replace "#obs2">>[img[images/NEWS2.png]]<</replace>><</click>> <div id="obs2"></div> <</if>>
<</nobr>><<set $storyprogress = 2>>
The point of care COVID-19 test is <<timed 1s>>.<<next>>.<<next>>.<<next>> ''positive.''<</timed>>
<<timed 7s>> Nurse Boyle has re-checked Mr Quail's observations as he looks more short of breath on returning to the department (available to view in the sidebar).
Mr Quail asks if there is any treatment you are able to give him Dr $surname?
What would you like to start?
Aciclovir <<radiobutton "$dexamethasone" "incorrect">>
Dexamethasone <<radiobutton "$dexamethasone" "correct">>
Ivermectin <<radiobutton "$dexamethasone" "incorrect">>
Miltefosine <<radiobutton "$dexamethasone" "incorrect">>
Pentamidine <<radiobutton "$dexamethasone" "incorrect">>
[[Prescribe]]
<</timed>><<if $medrec eq "0">>
<<if $dexamethasone eq "correct">> Well done, Dr $surname! As per RECOVERY Trial data, dexamethasone shows a mortality benefit for patients with COVID-19 and an oxygen requirement. <</if>>
<<if $dexamethasone neq "correct">> Unfortunately, Dr $surname, the only treatment from the list that has shown any mortality benefit as of October 2021 (RECOVERY Trial) is dexamethasone.<</if>>
<</if>>
<<nobr>>
<<if $medrec eq "0">>
<<set $diabetes to random(2)>>
<<set $hypertension to random(2)>>
<<set $OA to random(1)>>
<</if>>
<</nobr>>
Some blood results are now available. Of note, CRP is elevated at 88mg/L (<10). Creatinine is elevated at 167umol/L (60-110), eGFR 42ml/min/1.73m2 (>60).
Neutrophils, LFTs, haemoglobin, coagulation screen, electrolytes, and glucose are within reference range.
Now that we have a diagnosis and initial blood results are available, you decide what to do about Mr Quail's regular medications for admission.
You clarify with Mr Quail and his electronic health record that he usually takes the following medications. Please click on any medications you wish to continue. Any medications not selected will be withheld.
<<nobr>>
<<if $diabetes eq "0">>
''Metformin 1g BD'' |Continue <<radiobutton "$D0" "continue">>| |Withhold <<radiobutton "$D0" "withhold">>| <</if>>
<<if $diabetes eq "1">>
''Empagliflozin 25mg OD'' |Continue <<radiobutton "$D1" "continue">>| |Withhold <<radiobutton "$D1" "withhold">>| <</if>>
<<if $diabetes eq "2">>''Metformin 1g BD'' |Continue <<radiobutton "$D2a" "continue">>| |Withhold <<radiobutton "$D2a" "withhold">>|
''Empagliflozin 25mg OD'' |Continue <<radiobutton "$D2b" "continue">>| |Withhold <<radiobutton "$D2b" "withhold">>| <</if>>
<</nobr>>
<<nobr>>
<<if $hypertension eq "0">>
''Ramipril 10mg OD'' |Continue <<radiobutton "$H0" "continue">>| |Withhold <<radiobutton "$H0" "withhold">>| <</if>>
<<if $hypertension eq "1">>
''Amlodipine 5mg OD'' |Continue <<radiobutton "$H1" "continue">>| |Withhold <<radiobutton "$H1" "withhold">>| <</if>>
<<if $hypertension eq "2">>
''Bendroflumethiazide 2.5mg OD'' |Continue <<radiobutton "$H2" "continue">>| |Withhold <<radiobutton "$H2" "withhold">>| <</if>>
<</nobr>>
<<nobr>>
<<if $OA eq "0">>
''Paracetamol 1g PRN'' |Continue <<radiobutton "$O0" "continue">>| |Withhold <<radiobutton "$O0" "withhold">>| <</if>>
<<if $OA eq "1">>
''Ibuprofen 400mg PRN'' |Continue <<radiobutton "$O1" "continue">>| |Withhold <<radiobutton "$O1" "withhold">>| <</if>>
<</nobr>>
''Salbutamol 100 micrograms inhaled PRN'' |Continue <<radiobutton "$A" "continue">>| |Withhold <<radiobutton "$A" "withhold">>|
[[Confirm choices ->pharmacist]]\_\_ <span class="greentext"> Bloods your senior wanted: </span> \_\_
"A coagulation screen is useful as a baseline before starting prophylactic anticoagulation." <<if $Coag>>Your reason was: <span class="greentext"> \_\_ $CoagReason \_\_</span><</if>>
“A CRP would be a useful indicator of inflammation/infection. If this is COVID-19, CRP may help indicate when to prescribe some immunosuppressive drugs."
<<if $CRP>>Your reason was: <span class="greentext"> \_\_ $CRPReason \_\_</span><</if>>
"A full blood count would tell us if there is a lymphopenia, which would be fit with COVID-19. A neutrophilia may suggest a bacterial infection. Eosinophilia may suggest there is an asthmatic component. Haemoglobin would be useful to ensure the shortness of breath is not related to anaemia. A baseline platelet count is also needed before starting any prophylactic anticoagulation."
<<if $FBC>>Your reason was: <span class="greentext"> \_\_ $FBCreason \_\_</span><</if>>
"A glucose is useful to have as a baseline as we may want to start drugs that cause hyperglycaemia. We already know that Mr Quail has T2DM."
<<if $Glucose>>Your reason was: <span class="greentext"> \_\_ $GlucoseReason \_\_</span><</if>>
"Liver function tests are always useful to take as a baseline as many drugs we use in hospital can cause these to become abnormal. We also know that elevated AST and ALT can be associated with COVID-19 and some drugs to treat this may be contraindicated if these are too high."
<<if $LFT>>Your reason was: <span class="greentext"> \_\_ $LFTReason \_\_</span><</if>>
"Finally, I would check U&Es to ensure kidney function is unimpaired and electrolytes are within reference range, especially in the context of poor oral intake."
<<if $UE>>Your reason was: <span class="greentext"> \_\_ $UEReason \_\_</span><</if>>
\_\_ <span class="yellowtext"> Bloods your senior wouldn't request at the moment but wouldn't be unreasonable to send: </span> \_\_
"This patient presents with shortness of breath so it wouldn't be unreasonable to consider a pulmonary embolism. We know D-dimer may be also associated with COVID-19 prognosis. However, Mr Quail does not have any pleuritic chest pain at present and has had no haemoptysis. It might be worth performing a D-dimer if the chest X ray does not have changes that explain Mr Quail's symptoms."
<<if $Ddimer>>Your reason was: <span class="yellowtext"> \_\_ $DdimerReason \_\_</span><</if>>
"Again, troponin may be associated with COVID-19 prognosis, and we know myocarditis can result from COVID-19. However, the patient has no chest pain. If the ECG is abnormal then I would consider sending this investigation."
<<if $Troponin>>Your reason was: <span class="yellowtext"> \_\_ $TroponinReason \_\_</span><</if>>
\_\_ <span class="redtext"> Bloods your senior would not send: </span> \_\_
"There is no suggestion of encephalopathy - an ammonia level is not indicated."
<<if $Ammonia>>Your reason was: <span class="redtext"> \_\_ $AmmoniaReason \_\_</span><</if>>
"Mr Quail has no abdominal pain and therefore an amylase in not indicated."
<<if $Amylase>>Your reason was: <span class="redtext"> \_\_ $AmylaseReason \_\_</span><</if>>
"There are no strong features of adrenal insufficiency and so a cortisol is not indicated."
<<if $Cortisol>>Your reason was: <span class="redtext"> \_\_ $CortisolReason \_\_</span> <</if>>
While you wait for the bloods to come back, the chest X ray and ECG are ready to view:
[[View CXR]]
[[View ECG]]<<nobr>>
<<if ($diabetes eq "0") and ($D0 eq "continue")>> Sorry Dr $surname, as there is an AKI and oral intake is still poor, it would be safer to ''withhold the metformin'' for the time being. <</if>>
<<if ($diabetes eq "0") and ($D0 eq "withhold")>> Well done Dr $surname! As there is an AKI and oral intake is still poor, it would be safer to ''withhold the metformin'' for the time being. <</if>>
<<if ($diabetes eq "1") and ($D1 eq "continue")>> Sorry Dr $surname, as there is an AKI and oral intake is still poor, it would be safer to ''withhold the empagliflozin'' for the time being. <</if>>
<<if ($diabetes eq "1") and ($D1 eq "withhold")>> Well done Dr $surname! As there is an AKI and oral intake is still poor, it would be safer to ''withhold the empagliflozin'' for the time being. <</if>>
<<if $diabetes eq "2" and $D2a eq "continue">> Sorry Dr $surname, as there is an AKI and oral intake is still poor, it would be safer to ''withhold the metformin'' for the time being. <</if>>
<<if $diabetes eq "2" and $D2a eq "withhold">> Well done Dr $surname! As there is an AKI and oral intake is still poor, it would be safer to ''withhold the metformin'' for the time being. <</if>>
<<if $diabetes eq "2" and $D2b eq "continue">> Sorry Dr $surname, as there is an AKI and oral intake is still poor, it would be safer to ''withhold the empagliflozin'' for the time being. <</if>>
<<if $diabetes eq "2" and $D2b eq "withhold">> Well done Dr $surname! As there is an AKI and oral intake is still poor, it would be safer to ''withhold the empagliflozin'' for the time being. <</if>>
<</nobr>>
<<nobr>>
<<if $hypertension eq "0" and $H0 eq "continue">> Sorry Dr $surname, as there is an AKI and oral intake is still poor, it would be safer to ''withhold the ramipril'' for the time being. <</if>>
<<if $hypertension eq "0" and $H0 eq "withhold">> Well done Dr $surname! As there is an AKI and oral intake is still poor, it would be safer to ''withhold the ramipril'' for the time being. <</if>>
<<if $hypertension eq "1">> Whether to continue the amlodipine is more debatable, Dr $surname. As the patient is not hypotensive and COVID does not really tend to cause haemodynamic instability, it would not be unreasonable to ''continue the amlodipine''. This would not influence renal function. Conversely, you could argue that it would be safer to ''withhold the amlodipine'' given poor oral intake and the fact that the patient is not currently hypertensive. <</if>>
<<if $hypertension eq "2" and $H2 eq "continue">> Sorry Dr $surname, as there is an AKI and oral intake is still poor, it would be safer to ''withhold the bendroflumethiazide'' for the time being. <</if>>
<<if $hypertension eq "2" and $H2 eq "withhold">> Well done Dr $surname! As there is an AKI and oral intake is still poor, it would be safer to ''withhold the bendroflumethiazide'' for the time being. <</if>>
<</nobr>>
<<nobr>>
<<if $OA eq "0" and $O0 eq "continue">> Well done Dr $surname. ''There is no need to withhold the paracetamol'' as it is not nephrotoxic. The patient is of a reasonable weight and there is no evidence of liver pathology. It would be useful to continue the paracetamol for known joint pain and any discomfort caused by pyrexia/myalgia as a result of COVID. <</if>>
<<if $OA eq "0" and $O0 eq "withhold">> ''There is no need to withhold the paracetamol'' Dr $surname. Paracetamol is not nephrotoxic. The patient is of a reasonable weight and there is no evidence of liver pathology. It would be useful to continue the paracetamol for known joint pain and any discomfort caused by pyrexia/myalgia as a result of COVID. <</if>>
<<if $OA eq "1" and $O1 eq "continue">> Sorry Dr $surname, as there is an AKI and oral intake is still poor, it would be safer to ''withhold the ibuprofen'' for the time being. <</if>>
<<if $OA eq "1" and $O1 eq "withhold">> Well done Dr $surname! As there is an AKI and oral intake is still poor, it would be safer to ''withhold the ibuprofen'' for the time being. <</if>>
<</nobr>>
<<nobr>>
<<if $A eq "continue">> Well done Dr $surname! ''There is no reason to withhold a regular salbutamol inhaler'' from this patient. <</if>>
<<if $A eq "withhold">> Sorry Dr $surname, ''there is no reason to withhold a regular salbutamol inhaler'' from this patient. <</if>>
<</nobr>>
As Mr Quail now has an oxygen requirement, and no overt evidence of bacterial infection, your senior suggests introducing another medication. Which of the following classes of medication would be most appropriate?
Interleukin-1 receptor agonist <<radiobutton "$il" "incorrect">>
Interleukin-3 receptor antagonist <<radiobutton "$il" "incorrect">>
Interleukin-4 receptor antagonist <<radiobutton "$il" "incorrect">>
Interleukin-6 receptor antagonist <<radiobutton "$il" "correct">>
Interleukin-13 receptor agonist <<radiobutton "$il" "incorrect">>
[[Check with the local COVID-19 treatment algorithm->IL-6]]Mr Quail's chest X-ray has been performed. After several minutes of trying to remember your password, you eventually bring up the image:
<img src="images/cxr.png" width="500" height="500" alt="cxr">
<<nobr>>
<<set $cxr = 1>>
<<if $ecg eq "0">>[[View ECG]]
<<else>>
[[The point of care COVID test is ready->COVID]]
<</if>>
<</nobr>><img src="images/ecg.png" width="800" height="500" alt="ecg">
<<nobr>>
<<set $ecg = 1>>
<<if $cxr eq "0">>[[View CXR]]
<<else>>
[[The point of care COVID test is ready->COVID]]
<</if>>
<</nobr>><<nobr>>
<<set $medrec = 1>>
<<if (($D0 eq "continue" or $D0 eq "withhold") or ($D1 eq "continue" or $D1 eq "withhold") or (($D2a eq "continue" or $D2a eq "withhold") and ($D2b eq "continue" or $D2b eq "withhold")))
and
(($H0 eq "continue" or $H0 eq "withhold") or ($H1 eq "continue" or $H1 eq "withhold") or ($H2 eq "continue" or $H2 eq "withhold"))
and
(($O0 eq "continue" or $O0 eq "withhold") or ($O1 eq "continue" or $O1 eq "withhold"))
and
($A eq "continue" or $A eq "withhold")>>
[[A friendly pharmacist double-checks your plan with you...->medrec]]
<<else>>
Please decide what to do with ''every'' medication, Dr $surname. [[Return to medicines reconciliation->Prescribe]]
<</if>>
<</nobr>><<if $il eq "correct">> Well done Dr $surname! Tocilizumab and Sarilumab - interleukin-6 receptor antagonists - can both be used to attenuate the inflammatory response associated with severe COVID-19 disease (correct as of October 2021). <<else>> Unfortunately, your senior disagrees with your choice, Dr $surname! Tocilizumab and Sarilumab - interleukin-6 receptor antagonists - can both be used to attenuate the inflammatory response associated with severe COVID-19 disease (correct as of October 2021) <</if>>
You've done all you can for Mr Quail now and it's time to finish your shift. Your consultant thanks you for your effort and is even kind enough to give you some formal feedback for your portfolio!
[[Let's see how you did!]]<<if ($cough + $sob + $haemoptysis + $pyrexia + $taste + $vomit + $diarrhoea + $myalgia + $sorethroat + $chestpain + $headache) > 3>><img src="images/trophy.png" width="60" height="80" alt="trophy">''You've earned the "If You Know Your History" award!'' You asked enough questions in the history to show you played the game properly! Your password for collecting your medal is ''"IfYouKnow"''.<<else>><img src="images/notrophy.png" width="60" height="80" alt="notrophy">''Try playing through again to unlock this award''.<</if>>
<<if ($Cortisol or $Amylase or $Ammonia)>> <img src="images/notrophy.png" width="60" height="80" alt="notrophy"> ''Try playing through again to unlock this award''. <<else>> <img src="images/trophy.png" width="60" height="80" alt="trophy">''You've earned the "NHS Saviour" award!'' Thank you for not requesting unnecessary blood tests - you've saved money that can be used to benefit patients elsewhere! Your password for collecting your medal is ''"Saviour"''.<</if>>
<<if ($dexamethasone eq "correct" and $il eq "correct")>><img src="images/trophy.png" width="60" height="80" alt="jpl"> ''You've earned the "Management Master" award!'' You answered every question on COVID-19 management correctly. Your password for collecting your medal is ''"Master"''.<<else>><img src="images/notrophy.png" width="60" height="80" alt="noprize">''Try playing through again to unlock this award''.<</if>>
Thanks for playing the game!
You can either [[Restart the game->Restart]] or check out the [[credits]] and see how Mr Quail got on...
<<audio "background" volume 0.4 loop play>><<script>>
state.restart();
<<endscript>>
