# Supplementary figures and images for: Twine virtual patient games as an online resource for undergraduate diabetes acute care education
Source: BMC Med Educ. 2023 Jun 7;23:417. doi: 10.1186/s12909-023-04231-2 (PMC10244842; doi:10.1186/s12909-023-04231-2)

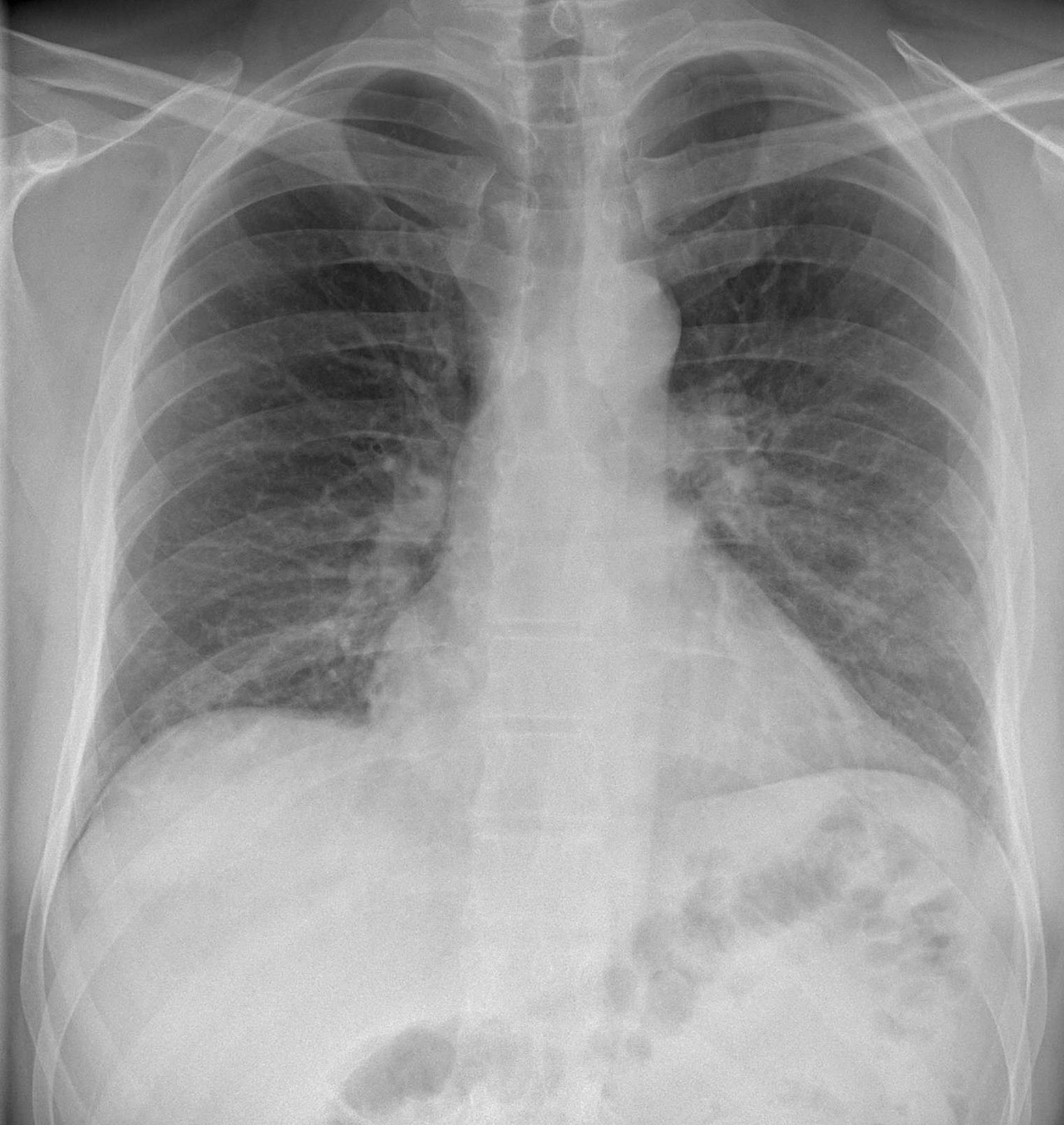

Supplement: Supplementary file 6 — Supplementary Material 6: Virtual Patient Blueprint Game [file 12909_2023_4231_MOESM6_ESM.zip › 12909_2023_4231_MOESM6_ESM/images/cxr.png]

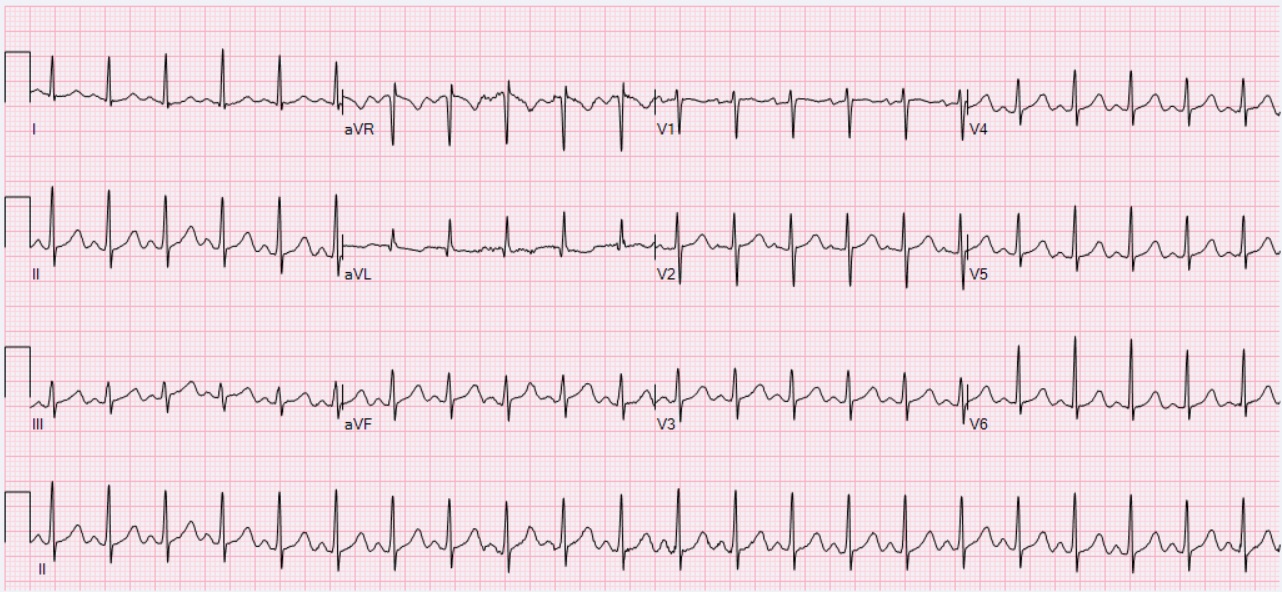

Supplement: Supplementary file 6 — Supplementary Material 6: Virtual Patient Blueprint Game [file 12909_2023_4231_MOESM6_ESM.zip › 12909_2023_4231_MOESM6_ESM/images/ecg.png]

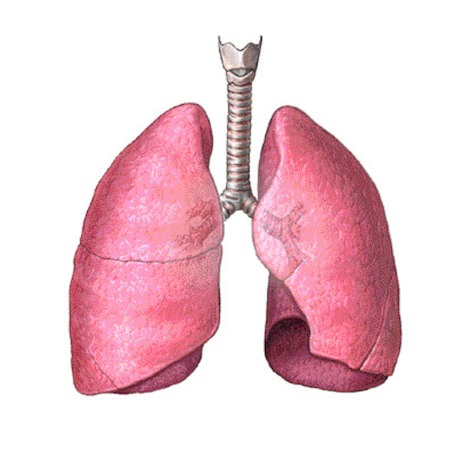

Supplement: Supplementary file 6 — Supplementary Material 6: Virtual Patient Blueprint Game [file 12909_2023_4231_MOESM6_ESM.zip › 12909_2023_4231_MOESM6_ESM/images/lungs.png]

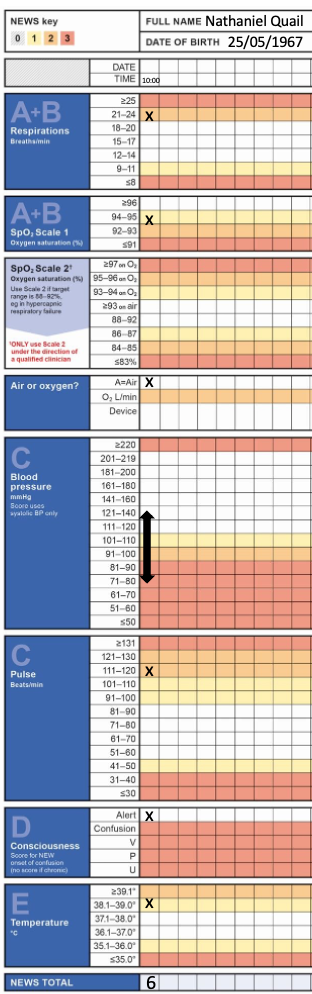

Supplement: Supplementary file 6 — Supplementary Material 6: Virtual Patient Blueprint Game [file 12909_2023_4231_MOESM6_ESM.zip › 12909_2023_4231_MOESM6_ESM/images/NEWS1.png]

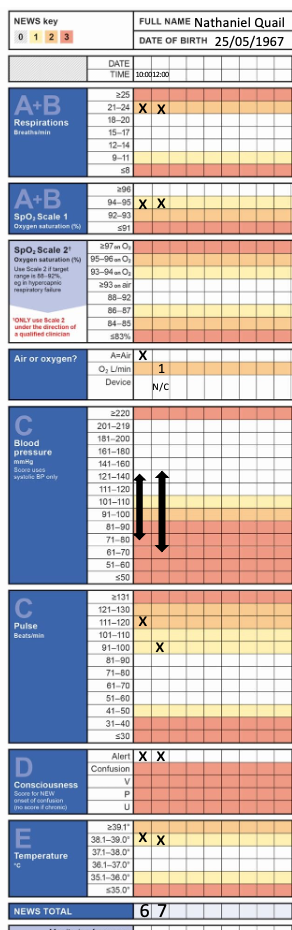

Supplement: Supplementary file 6 — Supplementary Material 6: Virtual Patient Blueprint Game [file 12909_2023_4231_MOESM6_ESM.zip › 12909_2023_4231_MOESM6_ESM/images/NEWS2.png]

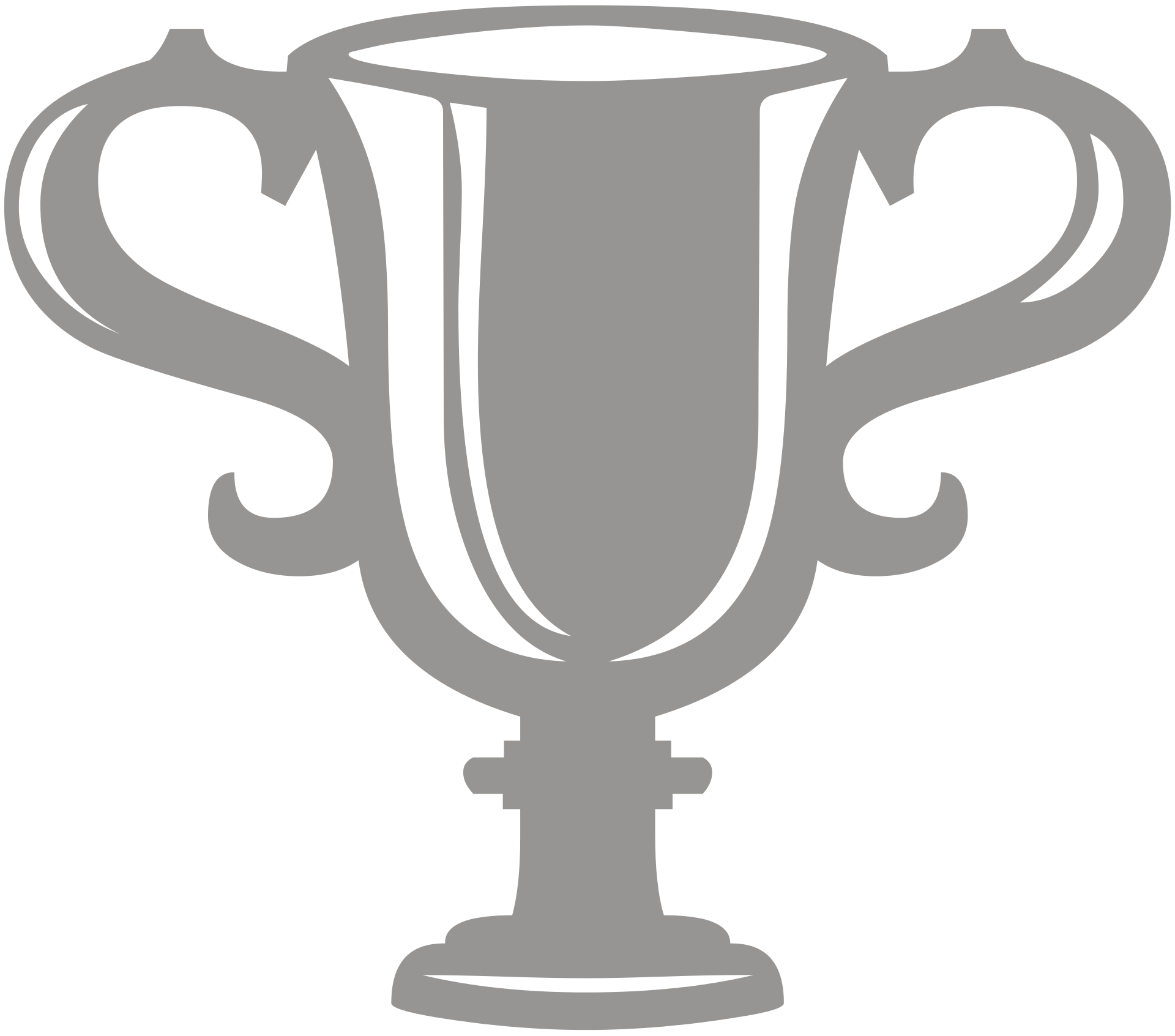

Supplement: Supplementary file 6 — Supplementary Material 6: Virtual Patient Blueprint Game [file 12909_2023_4231_MOESM6_ESM.zip › 12909_2023_4231_MOESM6_ESM/images/notrophy.png]

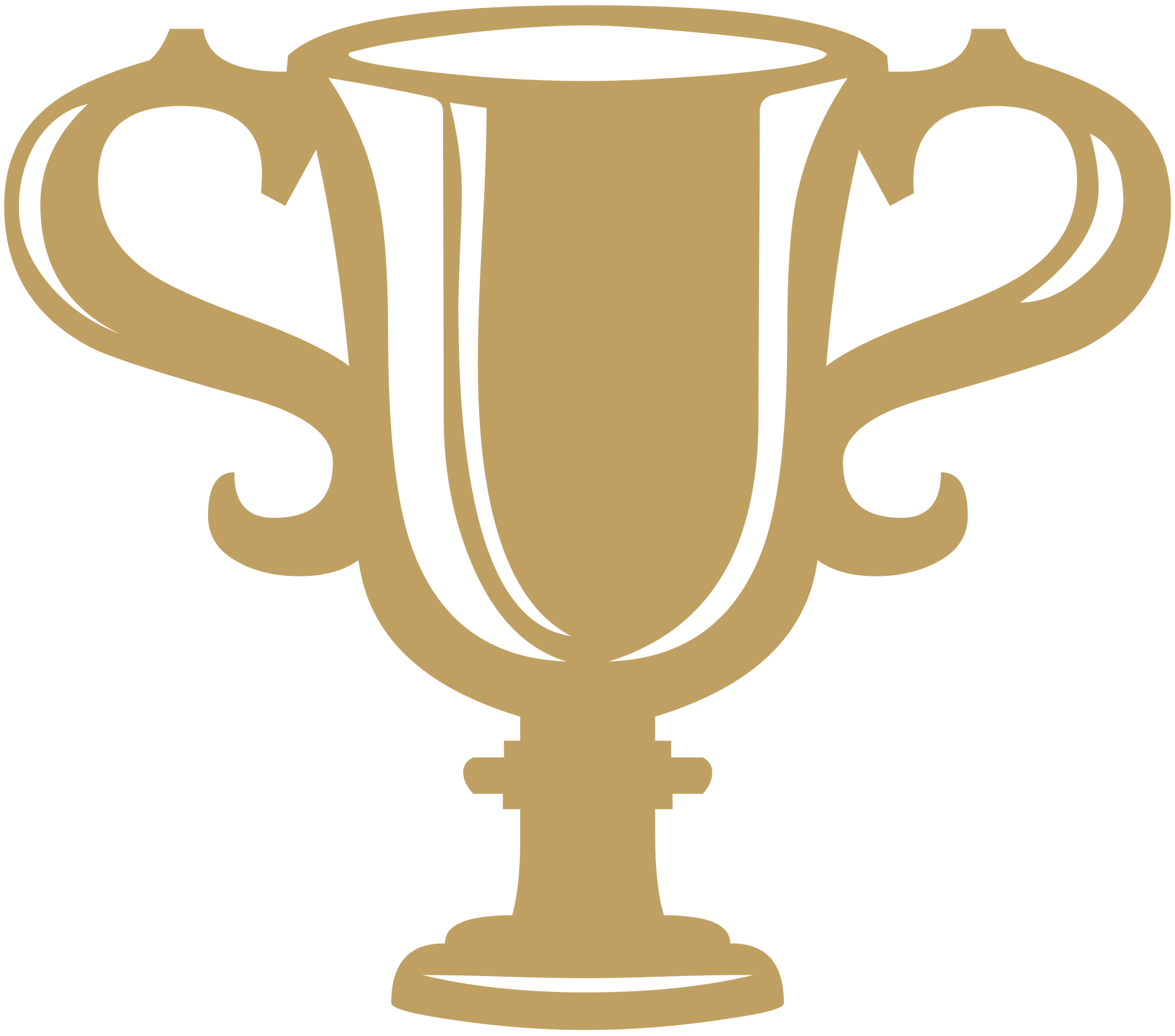

Supplement: Supplementary file 6 — Supplementary Material 6: Virtual Patient Blueprint Game [file 12909_2023_4231_MOESM6_ESM.zip › 12909_2023_4231_MOESM6_ESM/images/trophy.png]
