# Supplementary material for: Twine virtual patient games as an online resource for undergraduate diabetes acute care education
Source: BMC Med Educ. 2023 Jun 7;23:417. doi: 10.1186/s12909-023-04231-2 (PMC10244842; doi:10.1186/s12909-023-04231-2)
Supplement: Supplementary file 7 — Supplementary Material 7: Virtual Patient Blueprint Instructions [file 12909_2023_4231_MOESM7_ESM.docx]

File name: Additional file 7

File format: .docx

Title of data: Virtual Patient Blueprint Instructions

Description of data: The instructions for the Virtual Patient Blueprint Game. This will detail how the components of Additional File 6 should be assembled).

**Contents**

**Introduction - 2**

**Passages - 2**

**Introduction to Variables - 2**

**Naming the Player - 3**

**Loading Pictures, Videos, and Audio - 3**

**Free-Text History Taking - 5**

**Single Best Answer Questions - 9**

**Random Number Generation (RNG) - 9**

**Sidebar - 10**

**Cascading Style Sheets (CSS)- 11**

**Example Scenario Explained - 15**

**Introduction**

For this “how to” guide, we will be using the SugarCube story format. The documentation – available [here](http://www.motoslave.net/sugarcube/2/docs/#introduction) – provides information on the various aspects of the story format. It is a very good reference document, though it may appear daunting for any educator with little coding background. This document essentially serves as a beginner’s guide to creating medical virtual patient (VP) games using the SugarCube story format on Twine. A blueprint of a basic scenario is also provided. This guide is written with advice from the authors of the paper – who themselves had no background in coding prior to this project – and so easier or more sophisticated methods of presenting various aspects of the VP game may well be possible.

A special thanks to the Twine community, through the documentation above and Q&A forums, who helped us shape our VP games and therefore also help write this “how to” guide.

**Passages**

Twine stories are composed of linked passages. Each passage can link to one or more passages by enclosing the titles of the respective passages in “[[ ]]”. For example, the following input will show the player a link to the passage entitled “Examine the patient”:

*[[Examine the patient]]*

To keep things tidy, we can also display a link to the next passage under a different heading using “-->”. For example, in the following example the player would see a link that says “continue” to take them to the passage “Examine the patient”:

*[[Continue-->Examine the patient]]*

**Introduction to Variables**

Story variables are the building blocks of the gamification process, allowing the author to store and manipulate values throughout the game. They are created using a “$” symbol, followed by the name of the variable (no numerals directly after the $). For example, $1 would not work, however $variable1 or $v1 would. You can set variables to different values:

<<set $variableone = 0>>

<<set $variabletwo = 3>>

It is important to ensure that all the numerical variables have a value of 0 at the start of the game by creating a special passage called StoryInit and setting them all to 0.

Each time a player progresses in the game or answers a question, we can alter variables to track their progress. This can be used to assign a total score at the end of the game or provide achievements/rewards for specific actions. Variables are also utilised in the free-text history taking section.

The *<<if>>* function is a very important tool when using variables. This function essentially instructs the game to perform an action if certain criteria are met. For example:

*<<if $variableone eq “1”>> [[Continue->Section 2]] <</if>>*

In the above example, if $variableone has a value of 1 then the player will see an option to continue to the next passage.

**Naming the Player**

Use *<<textbox "$surname" "name">>* to generate a textbox where the player can enter their name. After this, you can use *Dr <<print$surname>>* to address the player at any point.


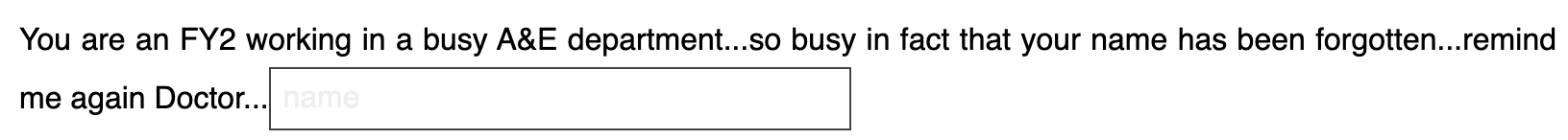


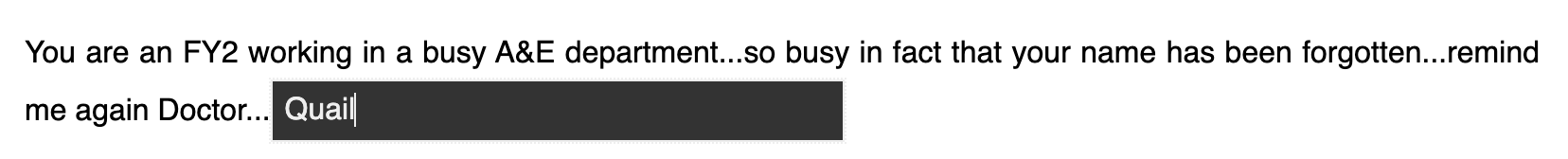


**Loading Images, Videos, and Audio**

First, ensure that you have a folder on your computer/laptop that contains the game (we will call it “example”). Within the “example”, create folders under the headings of “images”, “videos” and “audio”. Whenever you save your Twine game, make sure and save it to the “example” folder. We can set media to appear only if certain conditions are met using the <<if>> function. This will be explored in greater detail later.

**Images**

Say we had a picture of a trophy for an in-game reward. We would save it in the “images” folder and call it “trophy.png”. It could then be displayed in the passage using the following input:

*<img src="images/trophy.png" width="60" height="80" alt="trophy">*

The width and height can be changes – I found this was a good size for a small trophy. The “alt” displays the word “trophy” if there is an error in the image being displayed.

**Note: to test whether images, videos, or music work, you will need to export your Twine game into the “example” folder using the “Publish to File” option.**

There are ways to make media appear on the Test mode using JavaScript code, but this will not be explored here.

**Videos**

As before, save the video (e.g. “history.mp4”) in the “videos” folder. It can be displayed in the passage using the following input:

*<video src="videos/history.mp4" width="640" height="480" controls></video>*

Again, the video size can be adjusted by changing the width and height settings.

**Audio**

Loading music follows a similar process the other media above. As before, save the audio (e.g., “breathsounds.mp3”) in the “audio” folder. This time, however, we generally make use of a standalone passage called StoryInit (we create this ourselves as with any other new passage). This is used to load music for use within the rest of the game. In the StoryInit passage, put the following for each audio track you want to load (the audio we are loading in this example is “breathsounds.mp3”):

*<<cacheaudio "breathsounds" "audio/breathsounds.mp3">>*

Then, in the passage you want the breath sounds to appear:

*<<audio "breathsounds" volume 0.05 loop play>>*

With the volume being adjusted by changing the number 0.05, and the audio being looped by including “loop”, as above.

In the passage you want the audio to stop:

*<<audio "breathsounds" stop>>*

**Free-Text history Taking**

This is a complex area of Twine coding, and a further example and explanation will be given with the example game.

Set up the first passage like this:

*Ask about as many symptoms as you can think of.*

*<<textbox "$symptom" "">>*

*[[Ask about symptom]]*

*<<set $symptomcheck = 0>>*

At the start of the next passage, it is important to add the following:

*<<set $symptom to $symptom.toLowerCase()>>
<<set $symptom to $symptom.trim()>>*

This will ensure all spaces and capital letters are taken out of the player’s answer, allowing you to search for key words or phrases

You now need to think of synonyms and common misspellings for correct answers. A good way to ensure correct answers are picked up is to look for a short section of a word, which therefore limits any spelling mistakes the player can make. For example, instead of looking for “polydipsia”, you could look for “polyd”. This ensures the player is awarded the mark if they spell “polydipsia” incorrectly as “polydypsia”. Essentially, you can make it as strict as you want but the more lenient the marking then the more likely the player will be rewarded for a wrong answer.

See this example for 2 correct answers, which also consider lay synonyms for the medical terms:

*<<nobr>>*

*<<if $symptom .includes("polyd") or $symptom .includes("thirst") or $symptom .includes("drink")>><video src="videos/polydipsia.mp4" width="640" height="480" controls> </video>*

*<<set $polydipsia = 1>>*

*<<set $symptomcheck = 1>><</if>>*

*<<if $symptom .includes("polyph") or $symptom .includes("hung") or $symptom .includes("eat") or $symptom .includes("appet")>><video src="videos/polyphagia.mp4" width="640" height="480" controls> </video> <<set $polyphagia = 1>> <<set $symptomcheck = 1>> <</if>>*

*<</nobr>>*

The command *<<nobr>>* essentially removes whitespace created by lines of code when the player views the passage.

We set a variable associated with each correct answer to 1 (e.g., $polydipsia) so that this can be considered for end of game trophies. Notice that we set each variable to a value of 1 rather than adding 1 to a total score variable. This prevents the player from simply asking the same question multiple times to enhance their score.

Note that we set the variable *$symptomcheck* to 1 if any correct answer is given. We set this variable to 0 at the end of the previous passage in order to play an error message if no keyword from the history is mentioned:

*<<if $symptomcheck eq "0">> Brenda isn’t too sure about this symptom, Dr $doctor.<</if>>*

The whole passage might look like this:

*<<nobr>>*

*<<set $symptom to $symptom.toLowerCase()>>*

*<<set $symptom to $symptom.trim()>>*

*<<if $symptom .includes("polyd") or $symptom .includes("thirst") or $symptom .includes("drink")>><video src="videos/polydipsia.mp4" width="640" height="480" controls> </video>*

*<<set $polydipsia = 1>>*

*<<set $symptomcheck = 1>><</if>>*

*<<if $symptom .includes("polyph") or $symptom .includes("hung") or $symptom .includes("eat") or $symptom .includes("appet")>><video src="videos/polyphagia.mp4" width="640" height="480" controls> </video> <<set $polyphagia = 1>> <<set $symptomcheck = 1>> <</if>>*

*<<if $symptom .includes("polyur") or $symptom .includes("frequen") or $symptom .includes ("toilet")>><video src="videos/polyuria.mp4" width="640" height="480" controls> </video> <<set $polyuria = 1>> <<set $symptomcheck = 1>> <</if>>*

*<<if $symptomcheck eq "0">> You aren't asking the right questions Dr $doctor.<</if>>*

*<</nobr>>*

*Remember to ask Brenda as much as you can.*

*[[Ask something else->What specific symptoms do you want to ask about?]]*

*Once you have finished taking your history, Nurse Boyle has a [[question->Point of Care Tests]] for you...*

You could also ensure that players are only allowed to progress once they have asked key questions in the history by including the following ending to the above passage:

<<if ($polyphagia + $polydispia + $polyuria) eq “3”>>Once you have finished taking your history, Nurse Boyle has a [[question->Point of Care Tests]] for you...<</if>>

The above example ensures the option to progress only appears once the player has gained a total of 3 points for asking about the above three symptoms. If you want the player to get more than 1 of the 3 before continuing, you could use:

*<<if ($polyphagia + $polydispia + $polyuria) > 1 >>Once you have finished taking your history, Nurse Boyle has a [[question->Point of Care Tests]] for you...<</if>>*

Another way to use this history-taking session would be to make a progress button appear after a certain period.

Firstly, in the passage before your history taking:

Ask as much history as you would like from the patient. They will tell you if they have experienced the symptoms.

<<textbox "$symptom" "">>

[[Ask about symptom]]

<<set $symptomcheck = 0>>

**<<if $timer eq “0”>> <<set $start to new Date()>> <</if>>**

Remember to set the variable $timer to 0 along with your other story variables in the first passage of the game

The section in bold basically attaches a timestamp to the variable $start on the first viewing of the passage within the game. We then set the $timer variable to 1 in the next passage so this timestamp on variable $start does not reset when going back and forth. In the next passage we make a new timestamp attached to the variable $end and this changes each time the passage is visited. We then use the difference between the $start and $end variables to time how long the player has been taking a history for and allow the option to continue after a certain timepoint:

*<<set $symptom to $symptom.toLowerCase()>>*

*<<set $symptom to $symptom.trim()>>*

*<<if $symptom .includes("polyd") or $symptom .includes("thirst") or $symptom .includes("drink")>><video src="videos/polydipsia.mp4" width="640" height="480" controls> </video>*

*<<set $polydipsia = 1>>*

*<<set $symptomcheck = 1>><</if>>*

*…*

*<<if $symptomcheck eq "0">> You aren't asking the right questions Dr $doctor.<</if>> Remember to ask Brenda as much as you can.*

*[[Ask something else->What specific symptoms do you want to ask about?]]*

***<<set $end to new Date()>>***

***<<set $duration = (($end-$start)/1000)>>***

***<<if $duration > 300>> Nurse Boyle has a [[question->Point of Care Tests]] for you...***

***<</if>>***

The variable $duration is the time spent taking a history in seconds. In the above example, once 300 seconds (5 minutes) have passed, the option to continue with the scenario appears.

This method can also be used to time a patient deterioration if the player is taking too long to complete the scenario. You would have to use different variable names for $start $end and $duration.

You can also use an “either or” route to progression, where the option to continue is presented if a minimum history taking score is met OR a certain time has elapsed. Essentially, combine both above examples and use “or” between the two conditions:

***<<if ($duration > 300) or (($polyphagia + $polydispia + $polyuria) > 1 )>>*** *Nurse Boyle has a [[question->Point of Care Tests]] for you...****<</if>>***

**Single Best Answer Questions**

Single best answer questions can be created using the following input:

*Option 1 <<radiobutton "$answer" "wrong">>*

*Option 2 <<radiobutton "$answer" "wrong">>*

*Option 3 <<radiobutton "$answer" "correct">>*

Each option changes the variable $answer to “correct” or “wrong”. Option 3 is correct in this example. In the next passage, we can use the <<if>> function to give the player feedback:

*<<if $answer neq “correct”>> Unfortunately that is the wrong answer. The correct answer was… <</if>>*

*<<if $answer eq “correct”>> Well done, that was the correct answer! <</if>>*

We use neq (does not equal) “correct” to consider players who have simply not selected any answer before proceeding.

**Random Number Generation (RNG)**

RNG can attach a “random” value to a variable. This can be used to make each playthrough different for the player. In the example below, we change the lab value of potassium based on the computer randomly selecting a value of either 0, 1, 2, 3, or 4.

*<<set $potassium to random(4)>>*

*The potassium value is:*

*<<if $potassium is 0>>3.7 mmol/L (3.5–5.3)<</if>>*

*<<if $potassium is 1>>4.2 mmol/L (3.5–5.3)<</if>>*

*<<if $potassium is 2>>4.6 mmol/L (3.5–5.3)<</if>>*

*<<if $potassium is 3>>5.0 mmol/L (3.5–5.3)<</if>>*

*<<if $potassium is 4>>5.1 mmol/L (3.5–5.3)<</if>>*

*How do these results inform your management?*

*[[Add potassium to fluids->Potassium Added]]*

*[[Omit any additional potassium->No Potassium]]*

We can then set up the three linked passages to give the player feedback, utilising <<else>>. In this example, potassium should be added if the value is less than 5 and omitted if the value is greater than or equal to 5. Remember, we are using the $potassium variable to decide whether the player is correct – this was the RNG output and not the potassium measurement associated with it.

In the Potassium Added passage:

*<<if $potassium <3>> Well done, you were right to add potassium! <<else>> Incorrect! <</if>>*

In the No Potassium passage:

*<<if $potassium >=3>> Well done, you were right not to add potassium! <<else>> Incorrect! <</if>>*

**Sidebar**

If you don’t want the players to be able to restart the game or save their progress, the sidebar can be hidden by entering the following into the CSS (click on the story name to access - discussed in more detail later):

*#ui-bar {*

*display: none;*

*}*

You can also keep the sidebar but remove the option to go to previous passages by entering the following in the Edit Story JavaScript section:

*Config.history.controls = false;*

This is useful when we have more complicated passages as it prevents variables from being changed erroneously.

If you want to keep the sidebar, you can update the contents based on the player’s progress. For example, a picture of latest observations could be included. To do this you must create a separate passage called “StoryCaption”. Anything within this passage will appear on the sidebar.

You can update observations by changing the value of a variable e.g., $observations based on player choices/progress. For example, if a player gives IV fluids to a hypotensive patient, this could change the value of $observations from a 2 to a 3, with the value of 2 displaying a blood pressure of 80/40mmHg on the sidebar, and a value of 3 displaying a blood pressure of 100/60mmHg on the sidebar:

*<<nobr>>*

*<<if $observations is "1">>*

*<img src="images/observations1.png" width="100" height="100" alt="observations1">
<<elseif $observations is "2">>
<img src="images/observations2.png" width="100" height="100" alt="observations2">
<<elseif $observations is "3">>
<img src="images/observations3.png" width="100" height="100" alt="observations3">
<</if>>
<</nobr>>*

Again, the inclusion of <<nobr>> and <</nobr>> essentially hides the section of code contained within it when the game is played. Without including this, the sidebar will contain empty space.

**Cascading Style Sheets (CSS)**

CSS, accessible by clicking on the name of the passage, is used to style the story content. With thanks to [Leon’s stylesheets](https://twinery.org/forum/discussion/743/leons-stylesheets) on Twinery.org (a very useful website for frequently asked questions) we styled our game as shown below.

*body {*

*background-color: lightblue;*

*}*

The above changes the background colour (note the American spelling of “color” when coding) of the story. As with all the examples below, the colour can be chosen using a pre-defined named colour or using HEX codes. For example, he HEX code #008041 gives a green colour.

*#story {*

*font-size: 100%;*

*margin: 10% 0 0 0;*

*text-align: center;*

*}*

The above provides our default layout for the text within the game.

*#passages {*

*background-color: white;*

*border: 2px solid silver;*

*border-radius: 1em;*

*display: inline-block;*

*min-height: 40%;*

*margin:auto;*

*margin-bottom: 5%;*

*}*

*.passage {*

*padding: 2em;*

*color: black;*

*font-size: 100%;*

*text-align: justify;*

*}*

The above justifies the text within the passage and makes the default font black.

*a.link-internal, a.link-external {*

*color: deepskyblue;*

*}*

*a.link-internal:hover, a.link-external:hover {*

*color: #006400;*

*text-decoration: none;*

The above makes the links within the game (to the next passage) a light blue colour, which then turn dark green when the mouse hovers over them.

*@media screen and (max-width: 960px) {*

*.passage { font-size: 90%;}*

*#passages { width: 70%; }*

*}*

*@media screen and (max-width: 840px) {*

*.passage { font-size: 87.5%; }*

*#passages { width: 80%; }*

*}*

*@media screen and (max-width: 720px) {*

*.passage { font-size: 75%; }*

*#passages { width: 90%; }*

*}*

Once again, courtesy of [Leon’s stylesheets](https://twinery.org/forum/discussion/743/leons-stylesheets), this important piece of code above adapts the game display to fit smaller devices like mobile phones.

*.redtext {*

*color: #FF0000;*

*}*

*.greentext {*

*color: #008000;*

*}*

Finally, the CSS stylesheet can be used to store font colours for use within the game. The above is an example of a red and green font.

Then if you wanted to highlight the words “penicillin allergic” in red within a passage:

*The patient is <span class="redtext"> penicillin allergic </span>*

**Example Scenario Explained**

**Passage – StoryInit**

All our variables are set to 0. We also initialise the background audio (described above).

**Passage – StoryCaption**

We use the following input to display different observation charts based on the variable $storyprogress, which we update as the patient becomes more hypoxic. As the observation chart is large, we allow the player to click on “View Observations” to display it. It then reverts to “View Observations” when the player changes passage. *<<nobr>>* is used to remove blank space associated with the code.

*<<nobr>>*

*<<if $storyprogress eq "1">> <<click "View Observations">><<replace "#obs">>[img[images/NEWS1.png]]<</replace>><</click>> <div id="obs"></div> <</if>>*

*<<if $storyprogress eq "2">> <<click "View Observations">><<replace "#obs2">>[img[images/NEWS2.png]]<</replace>><</click>> <div id="obs2"></div> <</if>>*

*<</nobr>>*

**Passage – Introduction**

We store the player’s name using a variable:

*<<textbox "$surname" "Surname">>*

Whenever we want to address the player by name in later passages, we simply say “Dr $surname”.

We also play some audio here (described above) using:

*<<audio "background" volume 0.4 loop play>>*

**Passage – Mr Quail**

This is the first of our more complicated passages. We use this as the base to return to when asking the free-text history (also explained above). As we only want the past medical history of Mr Quail to appear on first reading, we ensure it only appears *<<if $timer eq “0”>>*.

Once the history-taking has started, the $timer variable is set as 1 and this history paragraph will no longer display. We also use the $timer variable to timestamp the first viewing of the passage (*<<set $start to new Date()>>)* when $timer eq “0”.

We have a blank textbox for the player to take the history <<textbox "$symptom" "">>. The variable $symptom now stores the question the player asks.

Finally, we *<<set $symptomcheck = 0>>.* If the player’s question loads a video in the next passage, this variable is set as 1. It ensures the next passage will have an error statement if the question does not successfully load a patient video.

**Passage – Ask about symptom**

The first thing we do is:

*<<set $timer = 1>>
<<set $symptom to $symptom.toLowerCase()>>
<<set $symptom to $symptom.trim()>>*

As mentioned, setting the $timer to 1 means that the game will know the player has visited the **Mr Quail** passage before when they return to it. It will therefore not repeat the history paragraph and the initial timestamp will not be altered.

We alter the $symptom variable as above to condense the player’s question into a single word and make it all lowercase. This means we can then search it for key letters/numbers to identify if specific symptoms are part of the enquiry.

We then load each video as below:

*<<if $symptom .includes("cough") or $symptom .includes("phle") or $symptom .includes("mucous") or $symptom .includes("mucus") or $symptom .includes("spit") or $symptom .includes("coryz") or $symptom .includes("nose")>>*

*<video src="videos/cough.mp4" width="640" height="480" controls> </video>*

*<<set $cough = 1>>*

*<<set $symptomcheck = 1>>*

*<</if>>*

If the phrase is recognised from the history, then the video will play. We then score the player for asking about cough/coryzal/mucous symptoms by setting the $cough variable to 1. It is important to consider synonyms and common misspellings (“phlegm” might be misspelled as “phlem” and therefore we load the video if “phle” is present). We do also need to consider sensitivity vs specificity of the answer and find a balance we are happy with. For example, a player asking about “phlebitis” would have the cough video played as this contains “phle”.

As mentioned, we set $symptomcheck to 1 with each correct answer. If this variable remains 0 then we know no videos have been loaded:

*<<if $symptomcheck eq "0">> Mr Quail hasn't experienced anything like that, try asking some more questions Dr $surname.<</if>>*

We link back to the previous passage using *[[Ask something else->Mr Quail]]* to enable the player to ask more questions.

We would like the player to either ask about more than 3 correct symptoms or spend at least 20 seconds on this section before being allowed (but not forced) to continue. To do this, we use the following input, making use of the initial timestamp in the previous passage (the full method being described previously in this documentation):

*<<nobr>>*

*<<set $end to new Date()>>*

*<<set $duration = (($end-$start)/1000)>>*

*<<if ($duration > 20) or (($cough + $sob + $haemoptysis + $pyrexia + $taste + $vomit + $diarrhoea + $myalgia + $sorethroat + $chestpain + $headache) > 3 )>>*

*If you are ready to continue, let's [[examine Mr Quail]]*

*<</if>>*

*<</nobr>>*

**Passage – examine Mr Quail**

This passage makes use of interactive maps to let the player hear breath sounds from various areas of the lungs. First, we map out the coordinates of the image using something like [image map](https://www.image-map.net/).

We can then link an action to a click within these coordinates. In this case we play audio and display a textbox. Loading audio in this way requires the use of JavaScript within Twine. Therefore, instead of using “StoryInit”, we load our audio in a slightly different way within this passage:

*<audio id="vesicular">*

*<source src="audio/vesicular.mp3" type="audio/mpeg" />*

*</audio>*

*<audio id="covid">*

*<source src="audio/covid.mp3" type="audio/mpeg" />*

*</audio>*

We then use the following input to load the map, with the coordinates created using [image map](https://www.image-map.net/):

*<map name="lungs" id="lungs">*

*<area target="" coords="192,104,136,141,94,200,80,250,394,260,373,186,344,143,295,109,240,98" shape="poly" onClick="SugarCube.Dialog.setup('Upper Zones');SugarCube.Dialog.wiki('That sounds normal');SugarCube.Dialog.open();document.getElementById('vesicular').play() "/>*

*<area target="" coords="73,252,70,392,122,397,194,384,218,323,218,255" shape="poly" onClick="SugarCube.Dialog.setup('Mid+Lower Zones');SugarCube.Dialog.wiki('That does not sound normal...');SugarCube.Dialog.open();document.getElementById('covid').play() "/>*

*<area target="" coords="260,257,269,374,338,393,394,380,404,314,395,261" shape="poly" onClick="SugarCube.Dialog.setup('Mid+Lower Zones');SugarCube.Dialog.wiki('That does not sound normal...');SugarCube.Dialog.open();document.getElementById('covid').play() "/>*

*</map>*

*<div class="resizable imageMapObserve" style="width: 600px;">*

*<img usemap="#lungs" alt="lungs" <img src="images/lungs.png">*

*</div>*

Adapting code found [here](https://qjzhvmqlzvoo5lqnrvuhmg.on.drv.tw/UInv/Sample_Code.html#Main%20Menu) (MIT license) allows us to specify the image size in the above code.

Note that it is more complicated to introduce JavaScript that makes previous audio stop when new areas are clicked. We therefore use very short audio clips for these such as a single breath sound or murmur. You can also set up an image that links to other passages by clicking certain areas. This can be useful if you have an image of a ward and want the player to decide the next patient to see. The data-passage attribute would be used in this case (see guidance [here](http://www.motoslave.net/sugarcube/2/docs/#markup-html-attribute)).

**Passage – bloods**

This passage asks the player to choose appropriate blood tests. Rather than simply select tests and continue, we can ask the player to justify their option using a textbox:

*Ammonia <<checkbox "$Ammonia" false true unchecked>> <<textbox "$AmmoniaReason" "reason?">>*

In the next passage we show what we deem to be the correct reason and compare it to the player’s reason (if selected). In a similar fashion to the passages involving free-text history taking, we could look for key words in the explanations to mark them as correct or incorrect. We have not included this in our example as it would be quite bulky given the amount of blood tests we have offered, and the concept has already been explored above.

**Passage – senior check**

As above, we now explain the correct answers to the player, using the <<if $x>> to determine whether the player has asked for the investigation and display their reason if they have. We also utilise colours for each heading:

*__ <span class="greentext"> Bloods your senior wanted: </span> __*

Remember to define the colours in the CSS as shown previously. Note the “__“ at either side underlines the heading.

**Passages – view CXR and view ECG**

These passages are included to show how branching can be used to give players a choice of the most important investigations to view, before converging back to the main story pathway. We use the following code in each passage ($cxr or $ecg depending on the passage) to let the game know when both investigations have been viewed so it can proceed with the rest of the story:

*<<nobr>>*

*<<set $cxr = 1>>*

*<<if $ecg eq "0">>[[View ECG]]*

*<<else>>*

*[[The point of care COVID test is ready->COVID]]*

*<</if>>*

*<</nobr>>*

**Passage – COVID**

The first thing we do in this passage is *<<set $storyprogress = 2>>* to update the observations on the sidebar.

We then show an example of using the *<<timed>>* commands to imitate speech/draw suspense.

Finally, we have a simple multiple-choice question with the *<<radiobutton>>* command, which allows the player to select only one option per category.

**Passage – Prescribe**

This is the beginning of a complex set of passages to illustrate RNG in the context of medicines reconciliation. As we will return to this passage, we need to ensure information regarding the correct answer to the previous question is only displayed once. We also need to ensure that the RNG only happens once. In a similar fashion to before, we use the variable $medrec for this purpose. This will eq “0” on the first visit to the passage and is then set as eq “1” in the next passage. We then use this to only display the answer to the previous question and only perform the random number generation once when $medrec eq “0”. The RNG section is coded as follows:

*<<nobr>>*

*<<if $medrec eq "0">>*

*<<set $diabetes to random(2)>>*

*<<set $hypertension to random(2)>>*

*<<set $OA to random(1)>>*

*<</if>>*

*<</nobr>>*

This gives us 3 possible random states for $diabetes (0, 1, and 2), 3 for $hypertension, and 2 for $OA.

We then provide one or more different medications depending on the state of each variable using the <<if>> function:

*<<if $diabetes eq "2">>''Metformin 1g BD'' |Continue <<radiobutton "$D2a" "continue">>| |Withhold <<radiobutton "$D2a" "withhold">>|*

*''Empagliflozin 25mg OD'' |Continue <<radiobutton "$D2b" "continue">>| |Withhold <<radiobutton "$D2b" "withhold">>| <</if>>*

In the example above, when $diabetes eq “2” we give the player 2 separate medications to think about. We need to ensure that the variable associated with the <<radiobutton>> is different with each medication on a single randomised variable. This ensures the player can select an option of “withhold” or “continue” for each medication.

**Passage – pharmacist**

As it would make feedback difficult if neither “continue” or “withhold” is selected for all medications, this passage acts as a check that the player has selected an answer for every medication. The *<<if>>* command for this looks quite complicated but is fairly self-explanatory in that it ensures an option has been selected no matter what combination of medications is given. It gets more complicated as we associate more medications with a single variable. For example, to ensure that both options would be selected if $diabetes is randomised to 2 (as above), we must include the following within the <<if>> command:

*<<if>>…(($D2a eq "continue" or $D2a eq "withhold") and ($D2b eq "continue" or $D2b eq "withhold"))…<</if>>*

This section may take a bit of trial and error to make sure brackets are in the correct places.

If all the *<<if>>* criteria are met, then the player’s answer is evaluated in the next passage. If not, the player is returned to the previous passage to answer completely. As we now make $medrec eq “1”, the medications will not be randomised again, and the same list will appear.

**Passage – medrec**

Once the player has set every medication to “continue” or “withhold”, this passage gives feedback on each decision. It is simply a matter of copy and pasting the explanation for the medications attached to each randomised variable, including a “correct” message when the player appropriately continued or withheld the medication or an “incorrect” message when this is not the case. For example:

*<<if ($diabetes eq "0") and ($D0 eq "continue")>> Sorry Dr $surname, as there is an AKI and oral intake is still poor, it would be safer to ''withhold the metformin'' for the time being. <</if>>*

*<<if ($diabetes eq "0") and ($D0 eq "withhold")>> Well done Dr $surname! As there is an AKI and oral intake is still poor, it would be safer to ''withhold the metformin'' for the time being. <</if>>*

The passage finishes with another simple single best answer question using the *<<radiobutton>>* function.

**Passage – IL-6**

This simply corrects the player’s answer to the previous question and finishes the scenario.

**Passage - Let's see how you did!**

This is the award passage at the end of the game. If the player satisfies the criteria for the award then the trophy is displayed, and a congratulations message accompanies this. You can also include a code +/- html link to a website for the player to collect a prize or award associated with their permanent profile. For example, you could create a single “Fill in the Blank” H5P activity on the learning platform Moodle that asks for the award password. This could then be used to award a Moodle Badge to the player’s permanent profile when the activity is completed with the correct password.

If the award criteria are not satisfied, a blank trophy picture appears with a generic message asking the player to play through the game again to try and unlock the trophy.

The image associated with the outcome is included using *<img src="images/trophy.png" width="60" height="80" alt="trophy">*. We found that this size was reasonable.

In our example, we give awards for:

1 – Asking about more than three relevant symptoms when taking the free-text history. This is simply performed by adding the sum of the variables (each is set to “1” when the relevant symptom is mentioned) and awarding the trophy if the sum is > 3.

2 – Not taking unnecessary blood tests. This is trickier as we are awarding the trophy for answers that have not been selected. We therefore first specify that if any of the relevant boxes have been ticked ($Cortisol or $Amylase or $Ammonia) then the trophy should not be given. We can then use the *<<else>>* command to specify that the trophy should be given if this is not the case.

3 – Getting both single best answer questions correct. We simply specify that the award is given if both *<<radiobutton>>* variables eq “correct”. If we wanted to award the trophy for getting a minimum number of answers correct, we could increase a $total variable with every correct answer. To do this, we would use the command *<<if $answer1 eq “correct”>> <<set $total += 1>> <</if>>* after every single best answer question. The trophy would then be awarded if the total score is above a threshold, as with the first trophy.

Audio is loaded as before.

Finally, the option to restart the game is given (see the final passage below).

**Passage – Restart**

To restart the game and set all variables back to their original state, we link the player to a final passage containing only the following script:

*<<script>>*

*state.restart();*

*<<endscript>>*

**You’re ready to make your own game!**Feel free to copy and paste any sections of our blueprint into your own game – that’s what it’s there for!
