# Supplementary material for: Twine virtual patient games as an online resource for undergraduate diabetes acute care education
Source: BMC Med Educ. 2023 Jun 7;23:417. doi: 10.1186/s12909-023-04231-2 (PMC10244842; doi:10.1186/s12909-023-04231-2)
Supplement: Supplementary file 8 — Supplementary Material 8: Creative Commons/permissions for Twine Virtual Patient Games [file 12909_2023_4231_MOESM8_ESM.docx]

**Creative Commons/permissions for Twine Virtual Patient Games as an Online Resource for Undergraduate Diabetes Acute Care Education**

Please note that these files, as well as index.html, are all part of additional file 6.

To ensure the example game works correctly, file names must not be changed.

The file index.html should be placed in a folder. Within this folder, 3 other folders should be created. All .mp3 files should be added to a folder named “audio”. All .png files should be added to a folder named “images”. All .mp4 files should be added to a folder named “videos”.

**Permissions**

----------------------------------------------------------------

background.mp3 - Background music - Audio by "Hans Müller-Kray", retrieved from https://imslp.org/wiki/Trumpet_Concerto_in_E-flat_major%2C_Hob.VIIe:1_(Haydn%2C_Joseph) on 20/12/2021. CC: <https://creativecommons.org/licenses/by-sa/4.0/>

----------------------------------------------------------------

cxr.png - Chest X Ray - Image by "User Hellerhoff", retrieved from https://commons.wikimedia.org/wiki/Category:X-rays_of_COVID-19#/media/File:Covid-19-Pneumonie_gering_ausgepraegt_50M_-_CR_pa_-_001.jpg on 20/12/2021. CC: <https://creativecommons.org/licenses/by-sa/4.0/>

----------------------------------------------------------------

covid.mp3 - Crackles - Audio by "James Heilman", retrieved from https://commons.wikimedia.org/wiki/File:Crackles_pneumoniaO.ogg on 20/12/2021. CC: <https://creativecommons.org/licenses/by-sa/3.0/deed.en>

---------------------------------------------------------------

ecg.png - ECG - Image by "Ewingdo", retrieved from: https://commons.wikimedia.org/wiki/Category:ECG_of_sinus_tachycardia#/media/File:ECG_Sinus_Tachycardia_132_bpm.jpg on 10/03/2023. CC: <http://creativecommons.org/licenses/by-sa/4.0/>

----------------------------------------------------------------

lungs.png - Lungs - Image by "Khalid Qaf", retrieved from https://www.flickr.com/photos/dr-kh-qalam/51349530641/in/photolist-2mezLtc-2mezLsf on 20/12/2021. CC: <https://creativecommons.org/licenses/by/2.0/>

----------------------------------------------------------------

notrophy.png - No Trophy - https://commons.wikimedia.org/wiki/Category:Trophy_icons#/media/File:Cup_of_Silver.svg on 20/12/2021. <https://creativecommons.org/publicdomain/zero/1.0/deed.en>

---------------------------------------------------------------

trophy.png - Trophy - Image by Wikimedia, retrieved from:

Image by Wikimedia, retrieved from: https://commons.wikimedia.org/wiki/Category:Trophy_icons#/media/File:Cup_of_Gold.svg on 20/12/2021. <https://creativecommons.org/publicdomain/zero/1.0/deed.en>

----------------------------------------------------------------

vesicular.mp3 - Vesicular breath sounds - Audio by "TheSimTech", retrieved from http://www.thesimtech.org/audio on 20/12/2021. CC: <https://creativecommons.org/licenses/by-sa/4.0/>

----------------------------------------------------------------

All videos within additional file 6:

1. chestpain.mp4

2. throat.mp4

3. myalgia.mp4

4. headache.mp4

5. taste.mp4

6. sob.mp4

7. haemoptysis.mp4

8. nandv.mp4

9. pyrexia.mp4

10. ending.mp4

11. dog.mp4

12. diarrhoea.mp4

13. cough.mp4

are the property of the author (Nathaniel Quail). I grant permission for the audio and video in these files to be published in print and digital format by Springer Nature (e-signed N Quail 10/03/2023)

----------------------------------------------------------------

NEWS1.png and NEWS2.png are the property of the author (Nathaniel Quail). I grant permission for these image files to be published in print and digital format by Springer Nature (e-signed N Quail 10/03/2023)

Top of Form

Bottom of Form

Top of Form

Bottom of Form

Top of Form

Bottom of Form

Top of Form

Bottom of Form

Top of Form

Bottom of Form

Top of Form

Bottom of Form

Top of Form

Bottom of Form

Top of Form

Bottom of Form

Top of Form

Bottom of Form

Top of Form

Bottom of Form

Top of Form

Bottom of Form
